# Glioblastoma stem cells resist cuproptosis with circadian variation of copper levels

**DOI:** 10.1172/JCI192599

**Published:** 2026-01-02

**Authors:** Fanen Yuan, Xujia Wu, Huairui Yuan, Donghai Wang, Tengfei Huang, Po Zhang, Hailong Mi, Weichi Wu, Suchet Taori, Priscilla Chan, Kenji Miki, Maged T. Ghoche, Linjie Zhao, Kalil G. Abdullah, Steve A. Kay, Qiulian Wu, Jeremy N. Rich

**Affiliations:** 1 UPMC Hillman Cancer Center, Pittsburgh, Pennsylvania, USA.; 2 Department of Neurosurgery, University of Pittsburgh School of Medicine, Pittsburgh, Pennsylvania, USA.; 3 Lineberger Comprehensive Cancer Center, University of North Carolina, Chapel Hill, North Carolina, USA.; 4 Department of Neurology, Keck School of Medicine, University of Southern California, Los Angeles, California, USA.; 5 Department of Neurosurgery, State Key Laboratory of Biotherapy, West China Hospital, Sichuan University and Collaborative Innovation Center, Chengdu, China.; 6 Department of Medicine and; 7 Department of Neurology, University of Pittsburgh, Pittsburgh, Pennsylvania, USA.; 8 Department of Neurology, University of North Carolina, Chapel Hill, North Carolina, USA.

**Keywords:** Cell biology, Neuroscience, Oncology, Brain cancer, Stem Cells

## Abstract

Cuproptosis involves accumulation of intracellular copper that triggers mitochondrial lipoylated protein aggregation and destabilization of iron–sulfur cluster proteins, leading to cell death. Pharmacologic induction of cuproptosis has been proposed as a cancer therapy. Here, we find that glioblastoma (GBM) stem cells (GSCs) displayed relative resistance to cuproptosis with circadian variation of intracellular copper levels. CRISPR screening of copper regulators under concurrent treatment with copper ionophore or clock disruption revealed dependency on ATPase copper transporting alpha (*ATP7A*). Circadian control of copper homeostasis was mediated by the core clock transcription factor, brain and muscle ARNT-like 1 (*BMAL1*). In turn, *ATP7A* promoted tumor cell growth through regulation of fatty acid desaturation. Copper levels negatively fed back into the circadian circuitry through sequestosome 1/p62–mediated lysosomal degradation of BMAL1. Targeting the circadian clock or fatty acid desaturation augmented cuproptosis antitumor effects. Crosstalk between the core circadian clock and copper sustains GSCs, reshaping fatty acid metabolism and promoting drug resistance, which may inform development of combination therapies for GBM.

## Abstract 2

Cancer stem cells resist cuproptosis with circadian variation of copper levels. Targeting circadian clock function or fatty acid desaturation sensitizes cancer stem cells to cuproptosis.

## Graphical abstract

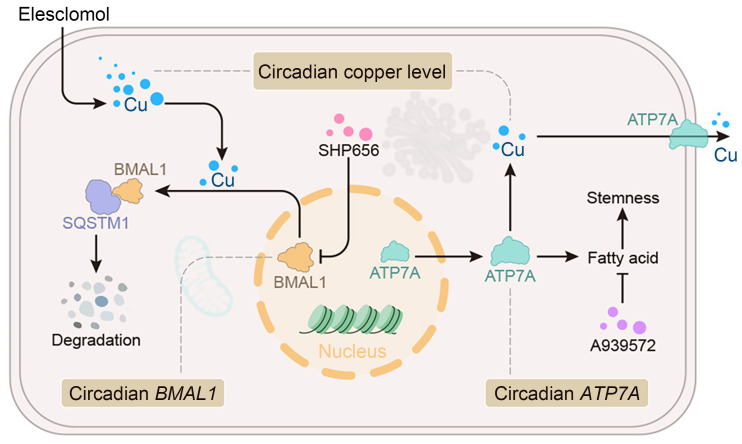

## Introduction

Glioblastoma (GBM) represents the most prevalent and malignant primary brain tumor, with a median survival under 2 years (1). One major challenge in GBM therapy is its marked heterogeneity, reflected in its original designation as glioblastoma multiforme. GBM cells form hierarchies topped by self-renewing, tumorigenic GBM stem cells (GSCs) that drive angiogenesis, invasion, and therapy resistance (2, 3). Despite their recognized importance, therapies targeting GSCs have yielded inconsistent clinical benefits.

Heavy metals have dual roles in cellular physiology. Iron and copper act as enzyme cofactors but are toxic when levels are imbalanced. Menkes disease is an X-linked disorder caused by ATPase copper transporting alpha (*ATP7A*) mutations on Xq13.3, leading to impaired copper transport. ATP7A is a transmembrane protein expressed in enterocytes, placenta, and the CNS that normally resides in the trans-Golgi network to deliver copper for cuproenzyme synthesis. When copper levels rise, ATP7A relocates to the plasma membrane to mediate efflux. Thus, metal homeostasis is tightly regulated, but copper and iron are often dysregulated in cancer. Enhanced iron uptake has been exploited for cancer imaging and therapy. We previously showed that GSCs upregulate transferrin receptors and ferritins to increase iron metabolism and promote proliferation (4). Excessive metal uptake can be detrimental, as both iron and copper trigger distinct forms of programmed cell death. Ferroptosis is an iron-dependent cell death characterized by the accumulation of toxic lipid peroxides (5). Tumor cells have high reactive oxygen species (ROS) levels, rendering them sensitive to ferroptosis. Copper also accumulates in brain tumors (6). Cuproplasia is a recently defined form of copper-dependent, regulated cell growth and proliferation, representing metalloplasia that encompasses both hyperplasia and neoplasia. (7). In contrast, cuproptosis is a copper-dependent cell death pathway in which copper binds directly to lipoylated components of the TCA cycle, causing aggregation of lipoylated proteins, loss of iron–sulfur cluster proteins, and lethal proteotoxic stress (8). Copper ionophores enhance cellular uptake of copper, inducing ROS-mediated death in tumor cells (9). Elesclomol (ES) and disulfiram (DSF), 2 common copper ionophores, enhance intracellular copper uptake, triggering cuproptosis and demonstrating promising antitumor properties in preclinical studies (10, 11). However, a randomized clinical trial combining DSF and copper with chemotherapy in patients with recurrent GBM failed to show survival advantage over chemotherapy alone (12), suggesting that GBMs have mechanisms of resistance against cuproptosis.

GBMs resist therapy through diverse mechanisms, including intratumor heterogeneity with GSCs at the apex of the cellular hierarchy. Others and we have shown that GSCs evade radiotherapy and chemotherapy through multiple molecular pathways (2, 13). As ROS maintains cancer stem cells, we hypothesized that cancer stem cells contribute to cuproptosis resistance.

GSCs exhibit distinct metabolic features compared with differentiated tumor cells and normal neural stem/progenitor cells (NSCs), including altered TCA cycle enzyme activity. GSCs preferentially depend on the circadian clock, which regulates TCA cycle function (14). The cellular clock has diverse and important roles in tumor development (15–17), and manipulation of the circadian clock has been proposed in cancer therapy (14). Given the clock’s role in metabolic regulation and the emergence of circadian-targeted therapies in cancer, linking circadian rhythm to treatment resistance may help overcome therapeutic failure in GBM. While ferroptosis and the cellular clock have been linked (18), the copper–clock connection in cancer remains unclear. Metal regulation has been extensively studied in plants, where circadian control of metal ion levels and transport contributes to diverse cellular processes, including protein synthesis, membrane function, and osmotic balance. In *Arabidopsis thaliana*, light and the circadian clock regulate copper deficiency, while elevated copper dampens nuclear clock components like GIGANTEA (19). These observations suggest that copper levels may be subject to circadian regulation. Here, we investigated the mechanisms underlying GBM resistance to therapies targeting copper homeostasis.

## Results

### Cancer stem cells are resistant to cuproptosis.

The failure of cuproptosis-inducing agents in GBM clinical trials led us to hypothesize that cancer stem cells contribute to cuproptosis resistance, given their well-documented resistance to other therapies, such as temozolomide, platinum compounds, and radiotherapy (2, 20, 21). GSC dependency on iron metabolism (4) suggested that GSCs may also display differential sensitivity to copper-dependent cell death. Therefore, we investigated the comparative cellular toxicity of a cuproptosis inducer (8), ES, within the tumor hierarchy. To confirm the copper-dependent specificity of ES, we treated tumor cells with ES in the presence of iron, copper, or zinc and measured cell proliferation. Copper supplementation (1 μM) sensitized cells to ES-induced cytotoxicity, whereas iron or zinc had no effect (Supplemental Figure 1A; supplemental material available online with this article; https://doi.org/10.1172/JCI192599DS1), supporting specificity of ES in copper homeostasis. Copper and ES cotreatment altered cuproptosis markers, decreasing FDX1 (ferredoxin 1) and LIAS (lipoic acid synthetase) and inducing DLAT (dihydrolipoamide *S*-acetyltransferase) aggregation (Supplemental Figure 1B). ES acts mainly as a pro-cuproptosis agent; thus, all experiments were performed in media supplemented with 1 μM CuCl_2_ unless noted otherwise.

**Figure 1:**
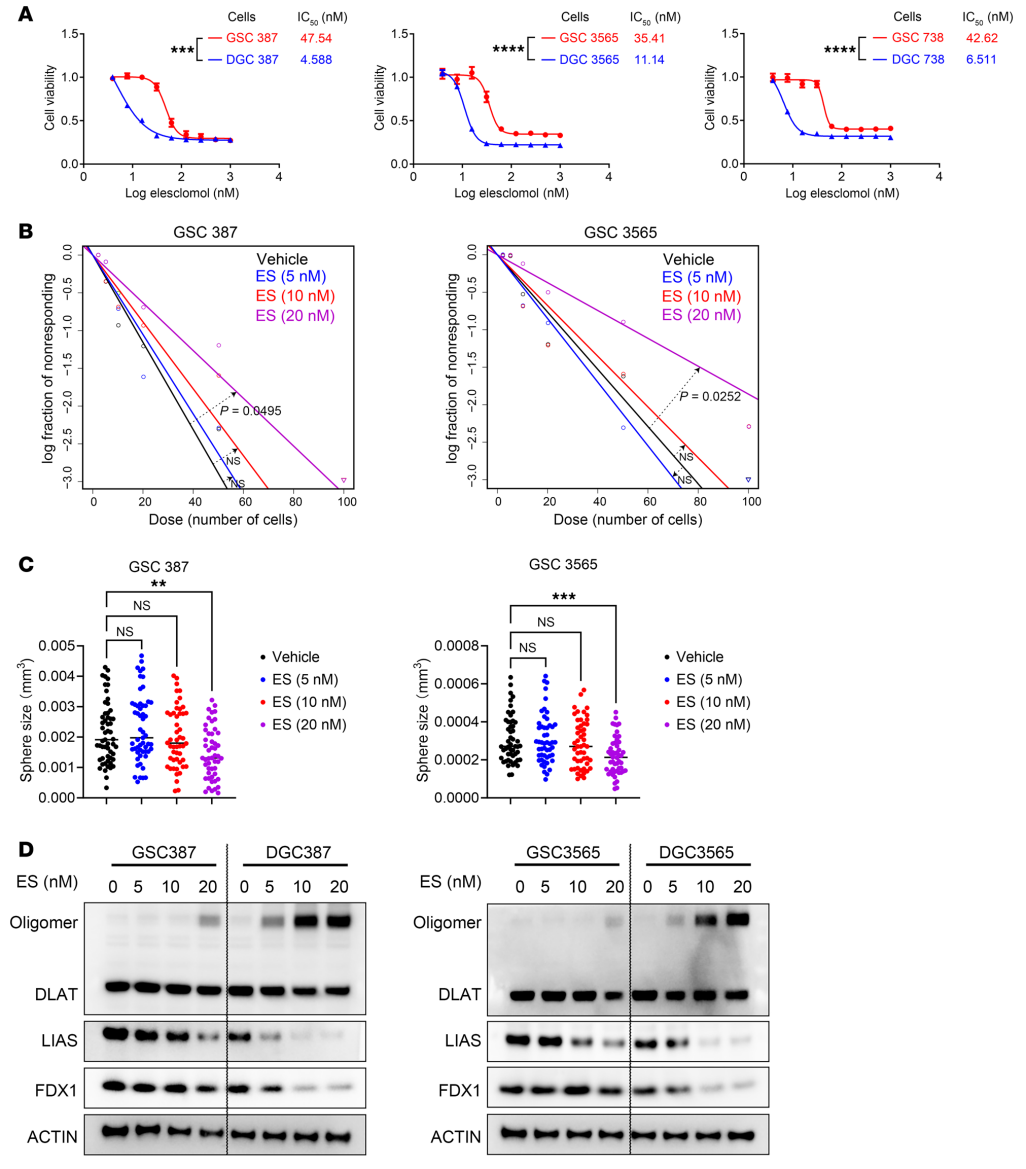
Cancer stem cells are resistant to cuproptosis. (**A**) IC_50_ values and dose–response curves of GSCs and DGCs treated with elesclomol (ES) for 48 hours in media containing 1 μM CuCl_2_. (**B**) Limiting dilution and (**C**) sphere formation assays of GSCs treated with ES (0–20 nM, 1 μM CuCl_2_). ZT, Zeitgeber time. (**D**) Immunoblots showing ES sensitivity at indicated concentrations for 48 hours. Log-transformed IC_50_ values were compared by *t* test (**A**), likelihood ratio test (**B**), and 1-way ANOVA with multiple comparisons (**C**). **P < 0.01, ***P < 0.001, ****P < 0.0001.

To test whether GSCs differ in their response to cuproptosis induction, we treated patient-derived GSCs and their matched differentiated GBM cells (DGCs) with ES in media containing 1 μM CuCl_2_. The IC_50_ values for ES in DGCs from 3 patients were low — 4.59 nM (DGC387), 11.14 nM (DGC3565), and 6.51 nM (DGC738) (Figure 1A) — suggesting potential therapeutic efficacy against bulk tumor. GSCs were less sensitive to ES-induced cuproptosis, with higher IC_50_ values (47.54, 35.41, and 42.62 nM) than DGCs (Figure 1A), suggesting intrinsic resistance that may underlie the failure of copper-based GBM therapies (12). This prompted us to investigate the mechanisms of GSC resistance to copper-induced cell death and identify strategies to overcome it.

Cancer stem cells are defined by their capacity for self-renewal, typically assessed by sphere formation. Given the relative resistance of GSCs to cuproptosis, we evaluated the effects of ES on GSC self-renewal. Extreme limiting dilution and sphere size showed that 20 nM ES modestly inhibited GSC self-renewal and proliferation, whereas lower concentrations were more effective against DGCs (Figure 1, B and C, and Supplemental Figure 1C). ES preferentially induced cuproptosis markers — DLAT oligomerization and reduced LIAS and FDX1 — in DGCs compared with GSCs, indicating differentiated cells were sensitive, whereas GSCs were resistant (Figure 1D and Supplemental Figure 1D).

### GSC copper levels oscillate with a circadian rhythm.

**Figure 2:**
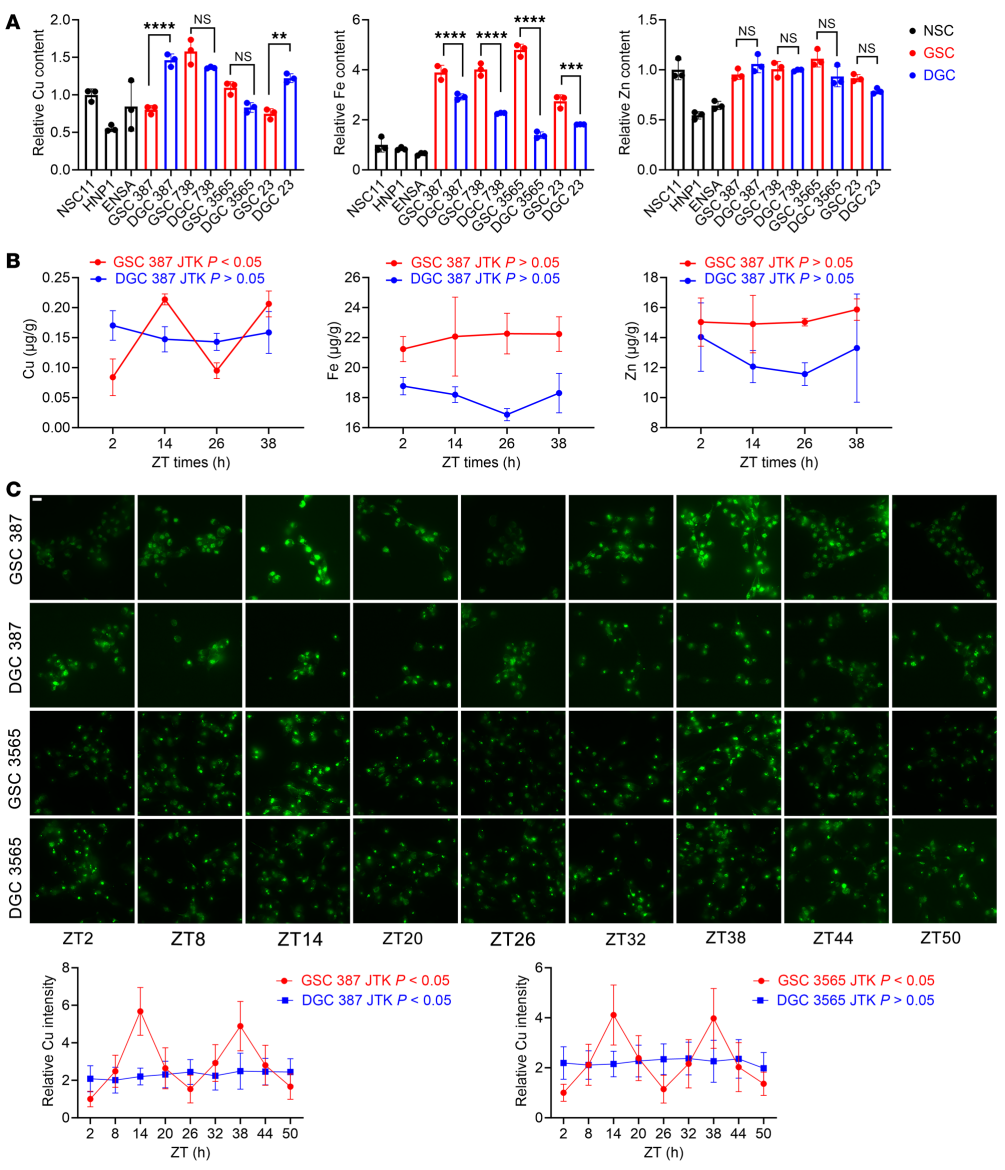
Copper levels display circadian oscillations specifically in GSCs. (**A**) ICP-MS analysis of copper, iron, and zinc in GSCs, DGCs, and NSCs. Data from 3 independent experiments are shown as mean ± SEM, normalized to NSC11. One-way ANOVA with multiple comparisons. (**B**) ICP-MS analysis of synchronized GSCs and DGCs (100 nM dexamethasone). Data (mean ± SEM, *n* = 3) analyzed for rhythmicity using the RTK (JTK_CYCLE-based) algorithm. (**C**) Live-cell copper imaging of GSC387, DGC387, GSC3565, and DGC3565 at 6-hour intervals after synchronization (100 nM dexamethasone). Relative fluorescence intensity was quantified. Scale bar: 20 μm. Rhythmicity determined by RTK; *P* < 0.05 indicates significant rhythmicity. **P < 0.01, ***P < 0.001, ****P < 0.0001.

GSCs preferentially accumulate iron (4), so we hypothesized that differential GSC sensitivity to cuproptosis may relate to altered copper regulation. We therefore quantified copper, iron, and zinc levels in GSCs, matched DGCs, and NSCs using inductively coupled plasma mass spectrometry (ICP-MS). Concordant with our prior observations (4), iron content was elevated in GSCs compared with DGCs and NSCs, whereas copper and zinc levels showed no significant differences among all cell types (Figure 2A). Building on our prior findings of circadian regulation in GSC metabolism (14), we investigated whether copper levels fluctuated temporally. We hypothesized that GSCs dynamically regulate copper with diurnal variation. Matched GSCs and DGCs were synchronized by a 2-hour dexamethasone pulse (100 nmol/L), followed by media replacement, and then metal levels were quantified by ICP-MS. Copper content displayed circadian oscillation in GSCs but not in DGCs, whereas iron and zinc showed no rhythmic variation in either cell type (Figure 2B). Copper imaging corroborated these findings (Figure 2C). For copper imaging analysis, GSC3565 exhibited rhythmicity whereas DGC3565 did not (Figure 2C). In addition, although both GSC387 and DGC387 exhibited rhythmic patterns, GSC387 showed a higher amplitude and more pronounced rhythmicity (Figure 2C).

To examine copper–circadian concordance, we analyzed gene expression data from asynchronously cultured 44 GSCs and 10 NSCs (GSE119834) (22). Using the Gene Ontology (GO) circadian rhythm signature (GOBP_CIRCADIAN_RHYTHM, GO:0007623), we found that circadian activity was elevated in GSCs relative to NSCs (Supplemental Figure 2A). Similarly, copper homeostasis (GO:0006878) activity was elevated in GSCs versus NSCs (Supplemental Figure 2B). Next, we constructed a cuproptosis gene signature from 10 genes involved in cuproptosis (*FDX1*, *LIAS*, *LIPT1*, *DLD*, *DLAT*, *PDHA1*, *PDHB*, *MTF1*, *GLS*, and *CDKN2A*) (8). In GSCs, circadian rhythm and cuproptosis signatures were negatively correlated (Supplemental Figure 2C). These results suggested that GSCs regulate copper levels in a circadian manner, likely reflecting intrinsic molecular distinctions rather than synchronized time-of-day–dependent expression.

### ATP7A promotes GSC survival from cuproptosis and clock disruption.

**Figure 3:**
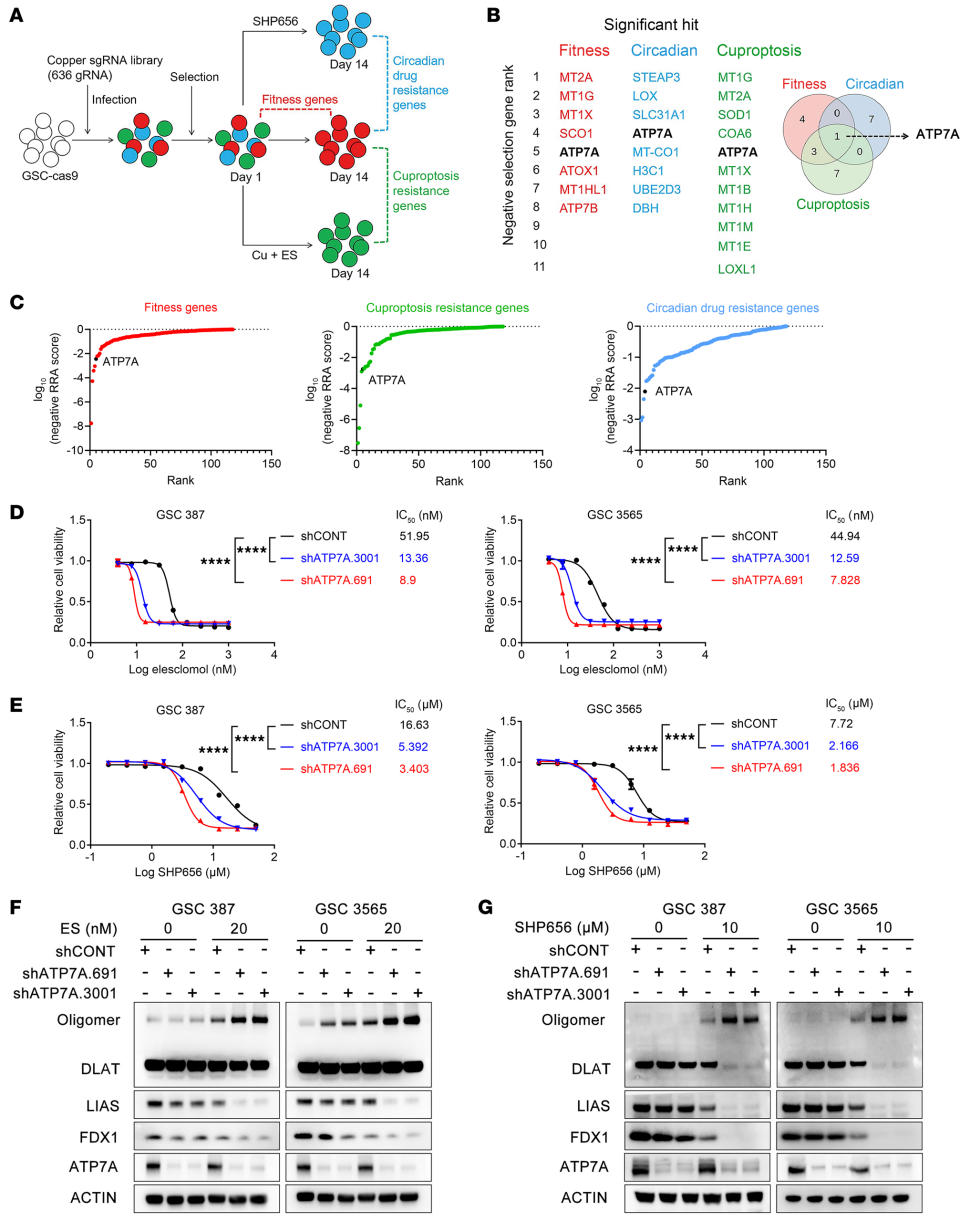
ATP7A is a GSC dependency gene to survive cuproptosis and clock disruption. (**A**) Schematic of CRISPR knockout screens identifying fitness, circadian drug resistance, and cuproptosis resistance genes. SHP656 (8.5 μM) and Cu+ES (35 nM ES + 1 μM CuCl_2_) treatments were used. (**B**) Significant negative-selection hits (*P* < 0.05, MAGeCK) shown by Robust Rank Aggregation scores; Venn diagram depicts overlapping hits. (**C**) Gene rank plot from CRISPR screens; lower values indicate higher essentiality. ATP7A highlighted in black. (**D** and **E**) IC_50_ and dose–response curves of ES and SHP656 in shCONT and ATP7A-knockdown (shATP7A.691, shATP7A.3001) GSCs treated for 48 hours (1 μM CuCl_2_). (**F** and **G**) Immunoblots showing ES (20 nM, 48 hours) or SHP656 (10 μM) sensitivity in control and ATP7A-knockdown GSCs. One-way ANOVA followed by multiple comparisons for **D** and **E**. ****P < 0.0001.

To investigate contributions of key copper modulators bridging the circadian clock and cuproptosis, we conducted CRISPR/Cas9 knockout screens in GSCs (Figure 3A) using a custom library targeting 58 copper-related genes with additional control genes in 636 guide RNAs (gRNAs) (Supplemental Table 1). We performed chemogenomic screens with ES + CuCl_2_ or the circadian inhibitor SHP656 (23) to identify genes mediating resistance to cuproptosis or clock inhibition (Figure 3A). We employed pharmacologic CRY (cryptochrome) stabilizer SHP656, an orally bioavailable KL001 derivative that selectively stabilizes CRY2 and enhances its negative feedback on brain and muscle ARNT-like 1–CLOCK–mediated (BMAL1–CLOCK–mediated) transcription (23). SHP656 inhibits the growth of GSCs in vitro and prolongs survival in GSC-engrafted mouse models without affecting DGCs or NSCs (23). The IC_50_ of ES derived from Figure 1A and SHP656 (14) were used as working concentrations. We identified 8 fitness, 8 circadian resistance, and 11 cuproptosis resistance genes (Figure 3B and Supplemental Tables 2 and 3). The only gene that intersected among these hits was *ATP7A* (Figure 3, B and C). ATP7A, a P-type copper-transporting ATPase, regulates intracellular copper homeostasis, and its mutations cause lethal infantile Menkes disease (24). In response to elevated intracellular copper levels, ATP7A translocates to the plasma membrane to facilitate copper efflux, functioning as a copper exporter (24).

To validate the role of ATP7A in resistance to cuproptosis and clock inhibition, we modulated its expression and assessed IC_50_ changes for ES and SHP656. ATP7A knockdown enhanced GSC sensitivity to both agents (Figure 3, D and E). Overexpression of ATP7A rescued the increased sensitivity to cuproptosis or disruption of clock function caused by ATP7A knockdown (Supplemental Figure 2, D and E). ATP7A knockdown increased cuproptosis markers and enhanced ES or SHP656 effects (Figure 3, F and G, and Supplemental Figure 2, F and G), also reducing GSC self-renewal in limiting-dilution and sphere assays, further sensitizing cells to ES (Supplemental Figure 3, A and B).

Based on the impact of ATP7A modulation on GSC responses to cuproptosis, we next investigated its relative expression in GSCs. Chromatin immunoprecipitation sequencing (ChIP-Seq) revealed elevated acetylated H3K27 (H3K27ac) enrichment at the ATP7A promoter in GSCs compared with DGCs and NSCs (Supplemental Figure 3C), indicating epigenetic activation of ATP7A in the tumor hierarchy. In patient samples, ATP7A was elevated in isocitrate dehydrogenase-WT (IDH-WT) and 1p/19q–non-codeleted gliomas, increased with tumor grade, and correlated with poor prognosis (Supplemental Figure 3, D–J). qPCR confirmed higher ATP7A expression in GSCs than in DGCs or NSCs (Supplemental Figure 3K). We analyzed a single-cell glioma dataset for circadian rhythm and copper homeostasis gene expression, revealing preferential enrichment of these signatures in stem-like tumor cells (Supplemental Figure 3L). Immunofluorescence staining showed cytoplasmic localization of ATP7A in both GSCs and DGCs, with no major differences in subcellular distribution; however, ATP7A expression levels were higher in GSCs than in their matched DGCs (Supplemental Figure 3M). ATP7A expression was higher in GSCs than in matched DGCs (Supplemental Figure 3M).

To assess the function of ATP7A in malignant and nonmalignant neural cells, we silenced its expression in GSCs, DGCs, and NSCs using 2 independent shRNAs. ATP7A knockdown markedly reduced GSC proliferation (Supplemental Figure 4A). DGCs also showed reduced growth, though the effect was less pronounced, while NSCs exhibited only modest inhibition — one shRNA caused a mild decrease, whereas the other had no effect. Overall, the growth-suppressive impact of ATP7A loss was most evident in GSCs (Supplemental Figure 4A). ATP7A depletion reduced GSC proliferation measured by 5-ethynyl-2′-deoxyuridine (EdU) staining (Supplemental Figure 4B), markedly inhibited GSC self-renewal (Supplemental Figure 4, C and D), and suppressed the expression of the stemness markers, SOX2 and OLIG2 (Supplemental Figure 4E). *ATP7A* correlated with *SOX2* expression in patients with IDH-WT GBM (Supplemental Figure 5A). To confirm ATP7A’s role in cuproptosis, copper depletion with tetrathiomolybdate (TM) partially reversed the effects of ATP7A knockdown, indicating tumor-suppressive action is at least partly copper dependent (Supplemental Figure 5B).

### The core clock regulates ATP7A and copper levels.

**Figure 4:**
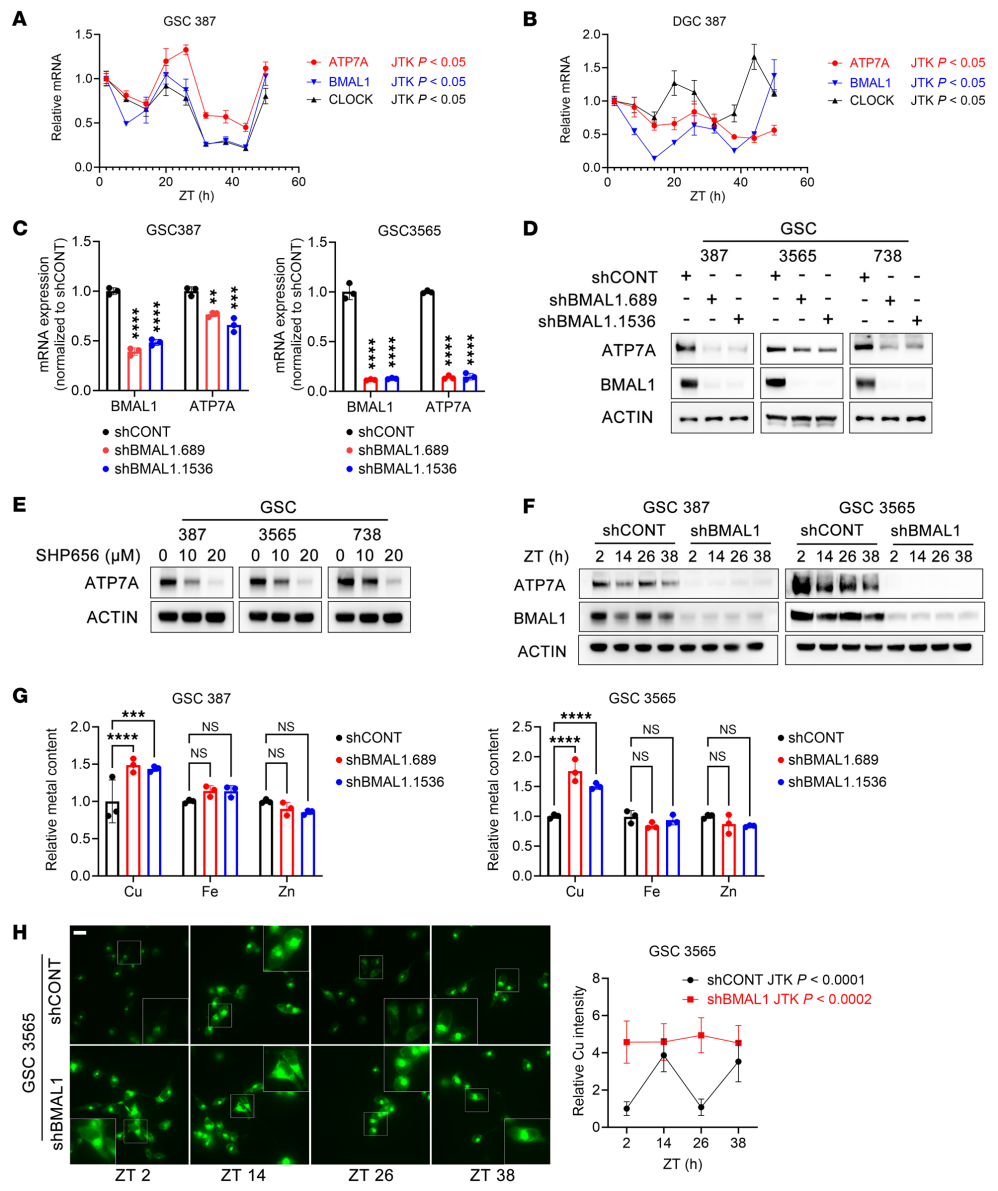
The core clock regulates ATP7A and copper levels. (**A** and **B**) qPCR analysis of ATP7A, BMAL1, and CLOCK expression at 6-hour intervals in synchronized GSC387 and DGC387 cells. sh, shRNA. (**C** and **D**) qPCR and Western blot of ATP7A expression in GSC387 and GSC3565 after BMAL1 knockdown (shBMAL1.689, shBMAL1.1536). (**E** and **F**) Western blot of ATP7A in GSCs treated with SHP656 or at 12-hour intervals following BMAL1 knockdown. (**G**) ICP-MS analysis of copper, iron, and zinc in GSC387 and GSC3565 after BMAL1 knockdown. (**H**) Live-cell copper imaging of synchronized GSC3565-shCONT and GSC3565-shBMAL1 cells at 12-hour intervals (100 nM dexamethasone). Relative fluorescence intensity was quantified. Scale bar: 20 μm. In **A**, **B**, and **H**, rhythmicity was analyzed using the JTK_CYCLE algorithm (*P* < 0.05 considered significant). One-way ANOVA with multiple comparisons was used for **C** and **G**. **P < 0.01, ***P < 0.001, ****P < 0.0001.

Given that intracellular copper levels exhibited circadian oscillation in GSCs and ATP7A regulates copper homeostasis, we next examined whether ATP7A is subject to circadian control. BMAL1, a core component of the mammalian circadian clock, forms a heterodimer with CLOCK to activate transcription of key clock genes, such as *Period* (*Per*) and *Cryptochrome* (*Cry*), via E-box promoter elements. The rhythmic expression of these genes establishes and maintains circadian regulation of diverse physiological processes, including metabolism and immune function (25). Our previous study has shown that BMAL1 plays an important role in glioma stem cells (14). We performed a circadian expression analysis of *CLOCK*, *BMAL1*, and *ATP7A* in matched GSCs and DGCs using JTK_CYCLE algorithm. Both cell types showed circadian variation, but DGC oscillations were less synchronized and consistent than those in GSCs (Figure 4, A and B). Notably, ATP7A showed stronger circadian amplitude in GSCs, indicating enhanced rhythmic regulation in the stem-like state (Figure 4, A and B). Consistently, ATP7A protein levels exhibited circadian oscillation relative to BMAL1 in GSCs but were less variable in DGCs (Supplemental Figure 5C).

Given the link between ATP7A and the transcriptional regulator BMAL1, we examined whether BMAL1 directly regulates ATP7A. ChIP-Seq revealed stronger BMAL1 binding at the ATP7A promoter in GSCs (Supplemental Figure 5D). This promoter region harbored canonical E-box motifs (CACGTG), known binding sites for the BMAL1–CLOCK heterodimer, potentially accounting for BMAL1 recruitment in GSCs. Consistently, ChIP-qPCR analysis confirmed BMAL1 occupancy at the *ATP7A* promoter in GSCs but not in DGCs (Supplemental Figure 5E). When cells are treated with a cuproptosis inducer to trigger copper-induced cell death, BMAL1 remains bound to the promoter region of *ATP7A* in GSCs but not in DGCs (Supplemental Figure 5F). BMAL1 showed rhythmic expression in both GSCs and DGCs. ATP7A rhythmicity occurred only in GSCs, likely due to stronger BMAL1 promoter binding. BMAL1 knockdown reduced ATP7A expression in GSCs but not in DGCs (Figure 4, C and D, and Supplemental Figure 6A). *ATP7A* expression positively correlated with *BMAL1* expression in patients with IDH-WT GBM in the Chinese Glioma Genome Atlas (CGGA; Supplemental Figure 6B). Although direct BMAL1 inhibitors are unavailable, compounds that suppress clock function via feedback stabilization were tested. Circadian inhibition reduced ATP7A expression in GSCs (Figure 4E and Supplemental Figure 6C). Genetic BMAL1 knockdown disrupted circadian ATP7A expression and reduced overall ATP7A expression (Figure 4F and Supplemental Figure 6D).

As BMAL1 regulates the copper exporter ATP7A, we examined its effect on copper levels. ICP-MS showed that BMAL1 knockdown in GSCs increased copper without affecting iron or zinc (Figure 4G). Live-cell imaging showed circadian oscillations of intracellular copper in control and BMAL1-deficient GSCs, though the rhythmic amplitude was markedly reduced in shBMAL1 cells (Figure 4H and Supplemental Figure 6E). This dampened oscillation was accompanied by elevated baseline copper levels (Figure 4H and Supplemental Figure 6E). ATP7A overexpression reversed copper accumulation from BMAL1 knockdown, indicating BMAL1 controls copper via ATP7A (Supplemental Figure 6F).

### Fatty acid metabolism mediates downstream effects of ATP7A.

**Figure 5:**
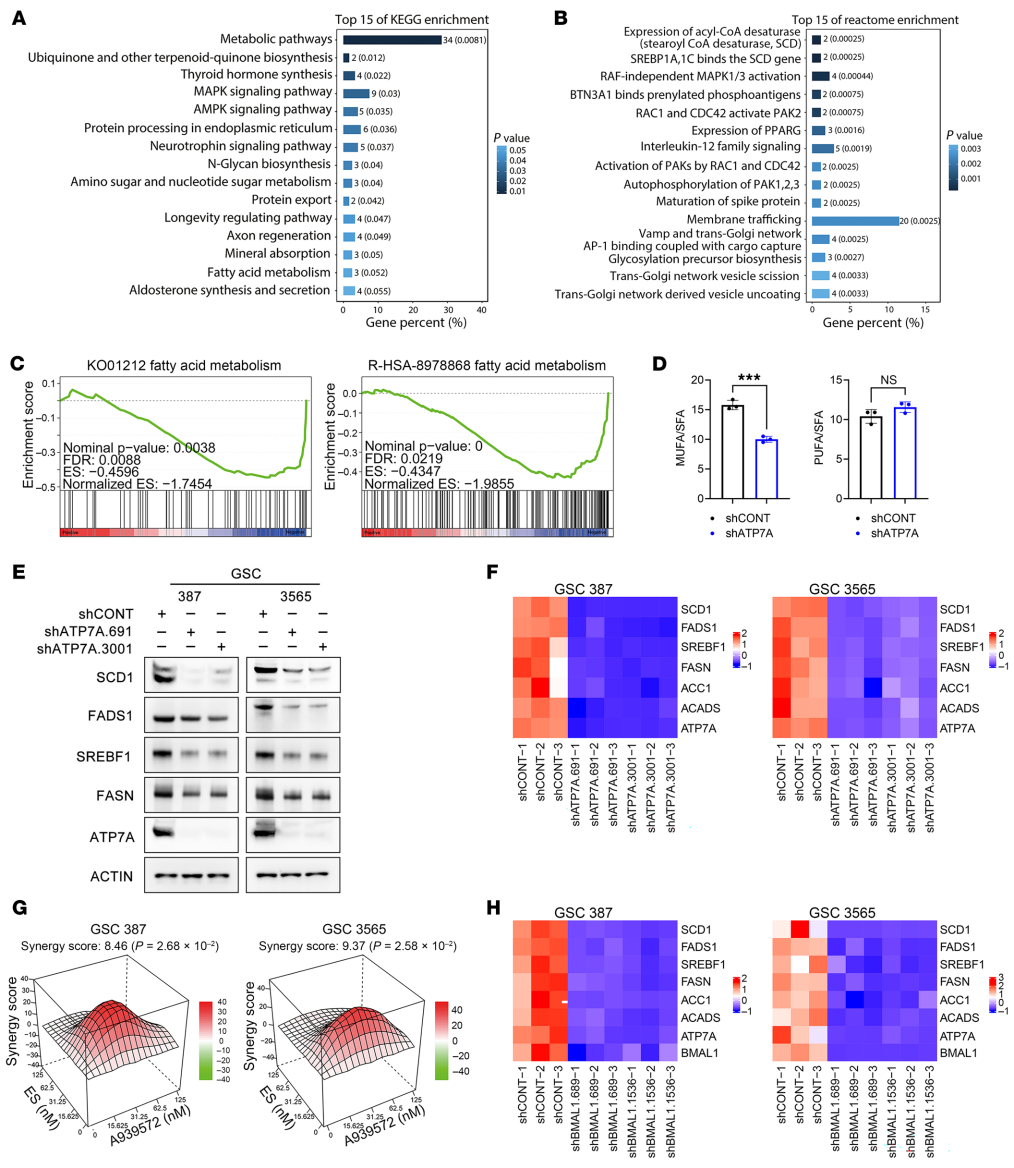
Fatty acid metabolism mediates downstream effects of ATP7A. (**A** and **B**) KEGG and Reactome enrichment analyses of differentially expressed genes (DEGs) upon ATP7A knockdown in GSC387, GSC3565, and GSC738, ranked by adjusted *P* values. (**C**) GSEA of DEGs in ATP7A-KD versus control GSCs using KEGG (K01212) and Reactome (R-HSA-8978868) signatures, showing normalized enrichment score (NES) and *P* values. (**D**) Lipidomics showing MUFA/SFA and PUFA/SFA ratios in GSC3565 after ATP7A knockdown. (**E**) Western blot of SCD1, FADS1, SREBF1, and FASN in GSCs following ATP7A knockdown. (**F**) qPCR of fatty acid–related genes (SCD1, FADS1, SREBF1, FASN, ACC1, ACADS) and ATP7A in GSC387 and GSC3565 with shATP7A.691 or shATP7A.3001. (**G**) Synergy plots (SynergyFinder) showing combined effects of SHP656 and SCD1 inhibitor A939572 in GSCs. (**H**) qPCR of fatty acid–related genes and ATP7A in GSCs after BMAL1 knockdown (shBMAL1.689 or shBMAL1.1536). ***P < 0.001.

To investigate how ATP7A promotes GSC growth, we compared RNA-Seq profiles of shATP7A- and shCONT-transduced GSCs. Kyoto Encyclopedia of Genes and Genomes (KEGG) analysis revealed changes in metabolic, mineral absorption, and fatty acid metabolism pathways (Figure 5A). Reactome analysis showed altered expression of acyl-CoA desaturase and SREBP1A/1C–stearoyl-CoA desaturase (SCD) binding pathways (Figure 5B). ATP7A knockdown positively correlated with gene sets associated with fatty acid metabolism on gene set enrichment analysis (GSEA) (Figure 5C).

Fatty acid synthesis promotes GBM growth, including GSC maintenance (26). SCD1 converts saturated to monounsaturated fatty acids, serving as a key regulator of lipid metabolism (27). Monounsaturated fatty acids (MUFAs) are essential for cancer cell survival (28). Lipidomics analysis showed that ATP7A depletion decreased the MUFA/saturated fatty acid (SFA) ratio while leaving the polyunsaturated fatty acid (PUFA)/SFA ratio unchanged (Figure 5D and Supplemental Table 4). ATP7A knockdown reduced SCD1, FADS1, SREBF1, and FASN protein levels (Figure 5E) and decreased fatty acid–related genes by qPCR (Figure 5F). *ATP7A* expression correlated with *SCD1* and *FADS1* in patients with IDH-WT GBM from CGGA (Supplemental Figure 7A). However, the heatmap of lipidomics measurements showed ATP7A depletion led to overall increased levels of all SFAs, MUFAs, and PUFAs (Supplemental Figure 7B and Supplemental Table 4). SFAs accumulated to cytotoxic levels, lowering MUFA/SFA ratios. SCD1 inhibition further reduces this ratio, while excess SFAs can convert to MUFAs or PUFAs, increasing their absolute levels. *SCD1* expression measured by qPCR was higher in GSCs compared with DGCs or NSCs (Supplemental Figure 7C). *SCD1* expression in patients with IDH-WT GBM from CGGA correlated with SOX2 and OLIG2 expression (Supplemental Figure 7D). To assess SCD1’s role in GSC maintenance, we inhibited it with A939572 (29), which reduced SOX2 and OLIG2 expression by Western blot and qPCR (Supplemental Figure 7, E and F).

The original description of cuproptosis noted a link to fatty acid biology (8), and copper regulates hepatic fatty acid synthesis. Thus, our findings indicate that ATP7A links circadian and copper metabolism while regulating fatty acid synthesis, suggesting that inhibiting fatty acid synthesis may enhance cuproptosis. Accordingly, combined treatment with the SCD1 inhibitor A939572 and the cuproptosis inducer ES produced a greater reduction in GSC growth (Figure 5G). To connect this process to the circadian clock regulation, BMAL1 knockdown in GSCs decreased expression of fatty acid–related genes (Figure 5H). Similarly, circadian inhibitors SR9009 and SHP656 decreased *SCD1*, *FADS1*, *SREBF1*, *FASN*, and *ACC1*, with minimal effect on *ACADS* (Supplemental Figure 7, G and H). Collectively, these findings suggest that circadian regulation of copper influences tumor cell behavior at least in part through fatty acid synthesis, revealing an additional therapeutic axis for intervention.

### Copper feeds back onto the clock through sequestosome 1/p62–mediated autophagy.

**Figure 6:**
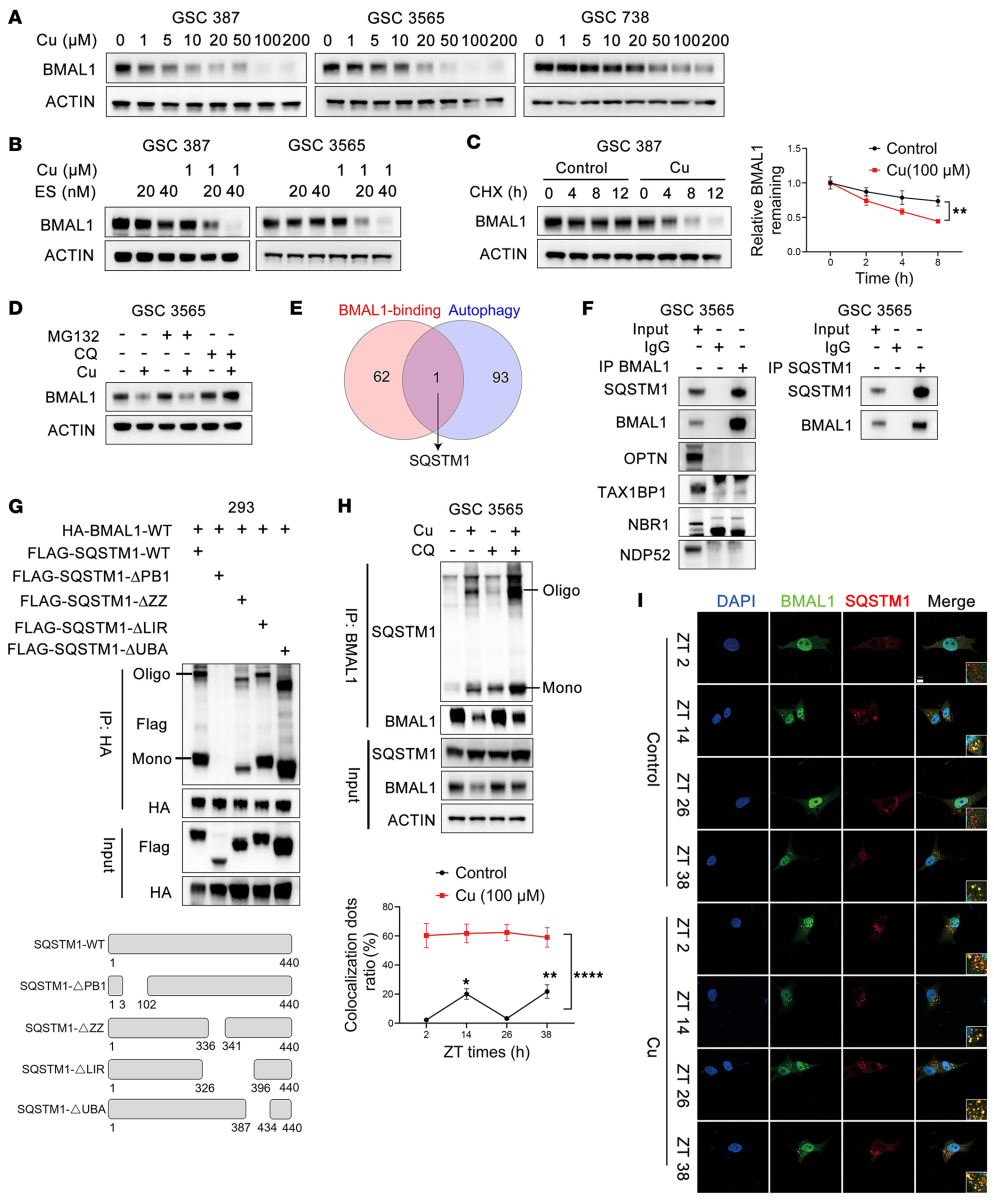
Copper feeds back onto the clock through SQSTM1-mediated autophagy. (**A** and **B**) Western blots showing BMAL1 expression in GSCs treated with increasing CuCl_2_ or ES ± 1 μM CuCl_2_. SQSTM1, sequestosome 1. (**C**) GSCs pretreated with CuCl_2_ (100 μM) or control for 24 hours, then exposed to CHX (100 μg/mL); BMAL1 stability assessed by Western blot. (**D**) BMAL1 levels in GSCs treated with MG132 (10 μM) or chloroquine (CQ; 10 μM) for 10 hours. (**E**) BMAL1-interacting proteins identified by mass spectrometry overlapping with GO:0061912 (selective autophagy). (**F**) Endogenous BMAL1 or p62 immunoprecipitated from GSC3565 and analyzed by immunoblotting; IgG served as control. (**G**) Co-IP of HA-BMAL1-WT and SQSTM1 fragments in HEK293T cells showing their interaction. (**H**) Endogenous BMAL1 immunoprecipitated from GSCs treated with indicated CuCl_2_ concentrations. (**I**) Immunofluorescence showing BMAL1–SQSTM1 colocalization at 12-hour intervals in synchronized GSCs ± CuCl_2_ (100 μM). Scale bar: 10 μm. *P < 0.05, **P < 0.01, ****P < 0.0001.

As BMAL1 regulates ATP7A, we tested whether copper affects the circadian clock. Increasing copper concentrations reduced BMAL1 expression in GSCs (Figure 6A). Similarly, ES decreased BMAL1 expression in the presence of copper (Figure 6B). Copper suppressed total protein levels and oscillations of BMAL1 in GSCs (Supplemental Figure 8A) but not *BMAL1* mRNA levels (Supplemental Figure 8B), suggesting that copper regulates BMAL1 at the posttranscriptional level. We treated GSCs with the translational inhibitor cycloheximide (CHX) following copper or control pretreatment and monitored BMAL1 protein levels over time, verifying that copper accelerates BMAL1 degradation (Figure 6C). Chloroquine elevates lysosomal pH, disrupting its acidic environment and blocking autophagosome–lysosome fusion, thereby inhibiting autophagic degradation. Chloroquine treatment prevented copper-induced BMAL1 degradation, whereas the proteasome inhibitor MG132 had no effect (Figure 6D), indicating copper-induced BMAL1 degradation involves the autophagy–lysosomal pathway.

We performed MS of BMAL1-binding proteins (Supplemental Tables 5 and 6), then prioritized among identified targets autophagy-related proteins (GOBP:SELECTIVE_AUTOPHAGY, GO0061912; Supplemental Table 7), demonstrating SQSTM1 as a top target (Figure 6E). IP confirmed that BMAL1 bound SQSTM1 but not other autophagy receptors (OPTN, TAX1BP1, NBR1, NDP52) (Figure 6F). To map the SQSTM1 domain interacting with BMAL1, FLAG-tagged SQSTM1 fragments were coexpressed with HA-BMAL1 in HEK293T cells for co-IP. Constructs lacking the PB1 domain (~80 amino acids mediating dimerization) failed to bind BMAL1 (Figure 6G). Oligomerization of SQSTM1 is essential for aggrephagy cargo assembly and autophagic degradation. Copper supplementation enhanced SQSTM1 oligomerization and its interaction with BMAL1, effects that were augmented by chloroquine treatment (Figure 6H and Supplemental Figure 8C). SQSTM1 oligomerization was markedly induced in response to cotreatment with copper and ES (Supplemental Figure 8D). Consistent with their interaction, BMAL1 and SQSTM1 colocalized in punctate cytoplasmic structures by immunofluorescence and exhibited circadian rhythmicity in synchronized GSCs (Figure 6I). Copper increased the number of BMAL1-P62/SQSTM1 colocalized puncta and abolished rhythmicity (Figure 6I). SQSTM1 or ATG7 knockdown blocked copper-induced BMAL1 degradation (Supplemental Figure 8, E and F). Under excess copper, SQSTM1 bound BMAL1 via its PB1 domain, inducing oligomerization and autophagic–lysosomal degradation, forming a negative feedback loop linking copper to circadian regulation. In DGCs, copper or ES treatment similarly reduced BMAL1 levels (Supplemental Figure 8, G and H), suggesting that exogenous excess copper induces BMAL1 degradation in tumor cells.

### Targeting the circadian clock augments efficacy of cuproptosis.

**Figure 7:**
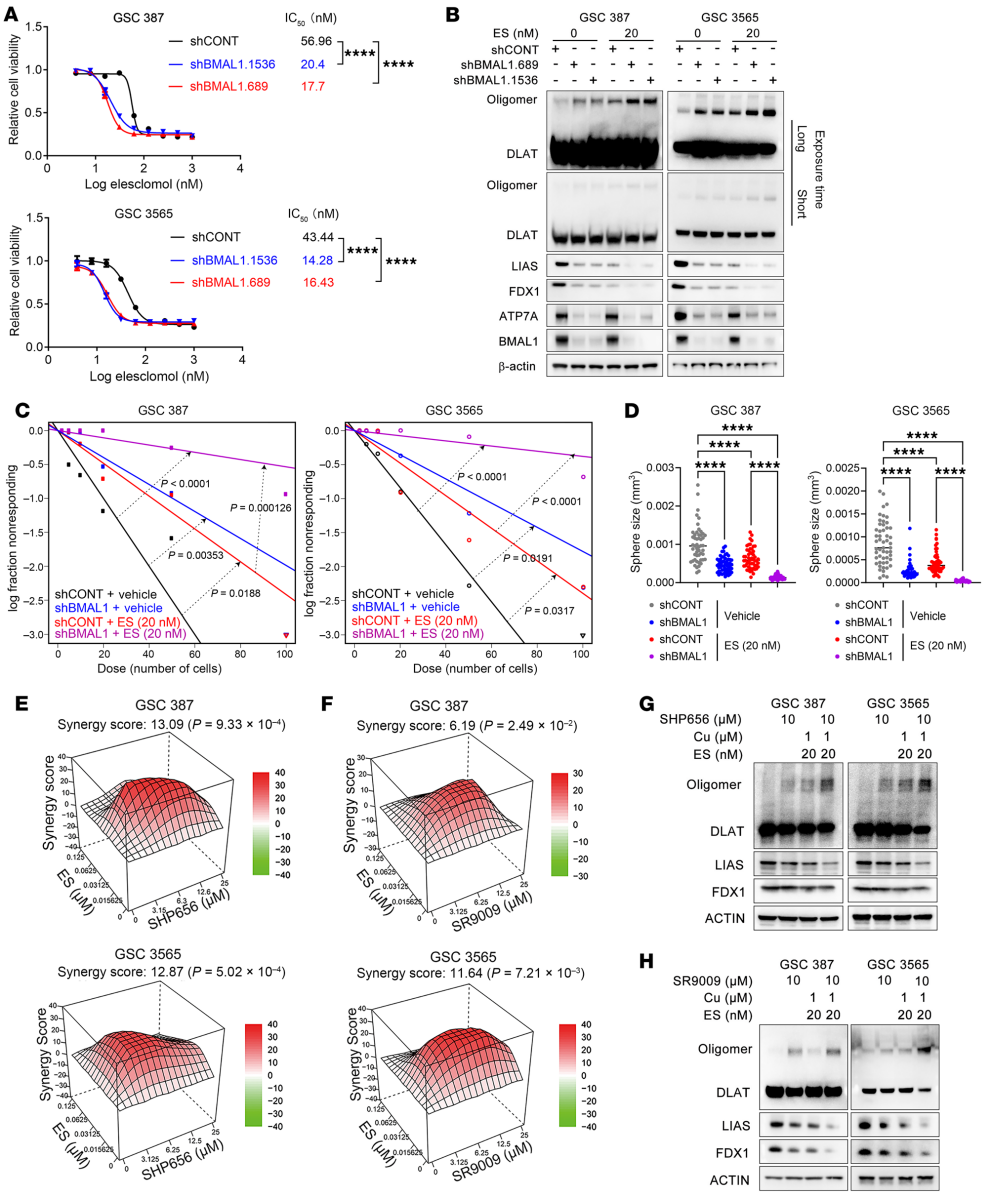
Targeting the circadian clock augments efficacy of cuproptosis. (**A**) Concentration–response curves of ES in GSCs with control or BMAL1 knockdown (shBMAL1.689, shBMAL1.1536) in media with 1 μM CuCl_2_. (**B**) Immunoblots showing (20 nM, 48 hours) ES–induced cuproptosis in control and BMAL1-knockdown GSCs. (**C**) Extreme limiting dilution and (**D**) sphere formation assays of control and BMAL1-knockdown GSCs treated with ES (20 nM, 1 μM CuCl_2_). *n* values as indicated. (**E** and **F**) Synergy plots (SynergyFinder) showing combined effects of SHP656 or SR9009 with ES in GSCs. (**G** and **H**) Western blots showing expression of DLAT, LIAS, and FDX1 in GSCs treated with indicated drugs. One-way ANOVA followed by multiple comparisons for **A** and **D**. Two-tailed likelihood ratio test for **C**. ****P < 0.0001.

To assess how circadian disruption influences cuproptosis sensitivity, we silenced BMAL1 and evaluated GSC and DGC responses. ATP7A overexpression rescued the enhanced cuproptosis sensitivity caused by BMAL1 knockdown in GSCs (Figure 7A). BMAL1 knockdown enhanced cuproptosis markers measured by Western blot in GSCs but not in DGCs (Figure 7B and Supplemental Figure 9A). BMAL1 depletion enhanced ES-induced inhibition of GSC self-renewal in limiting dilution and sphere assays (Figure 7, C and D, and Supplemental Figure 9B). Thus, genetically targeting the circadian clock sensitizes GSCs to cuproptosis.

Given the feedback loop between copper and the circadian clock, concurrent pharmacologic targeting of both pathways may enhance GSC suppression. Circadian inhibitors, including the CRY stabilizer SHP656 and the REV-ERB agonists SR9009 and SR9011, synergized with ES to augment anti-GSC efficacy (Figure 7, E and F, and Supplemental Figure 9C) without increasing toxicity in NSCs (Supplemental Figure 9D). Treatment with SHP656 or SR9009 enhanced cuproptosis markers on immunoblot, including increased DLAT oligomerization and reduced LIAS and FDX1 expression following ES treatment (Figure 7, G and H).

### Targeting circadian clock-ATP7A-cuproptosis in vivo.

**Figure 8:**
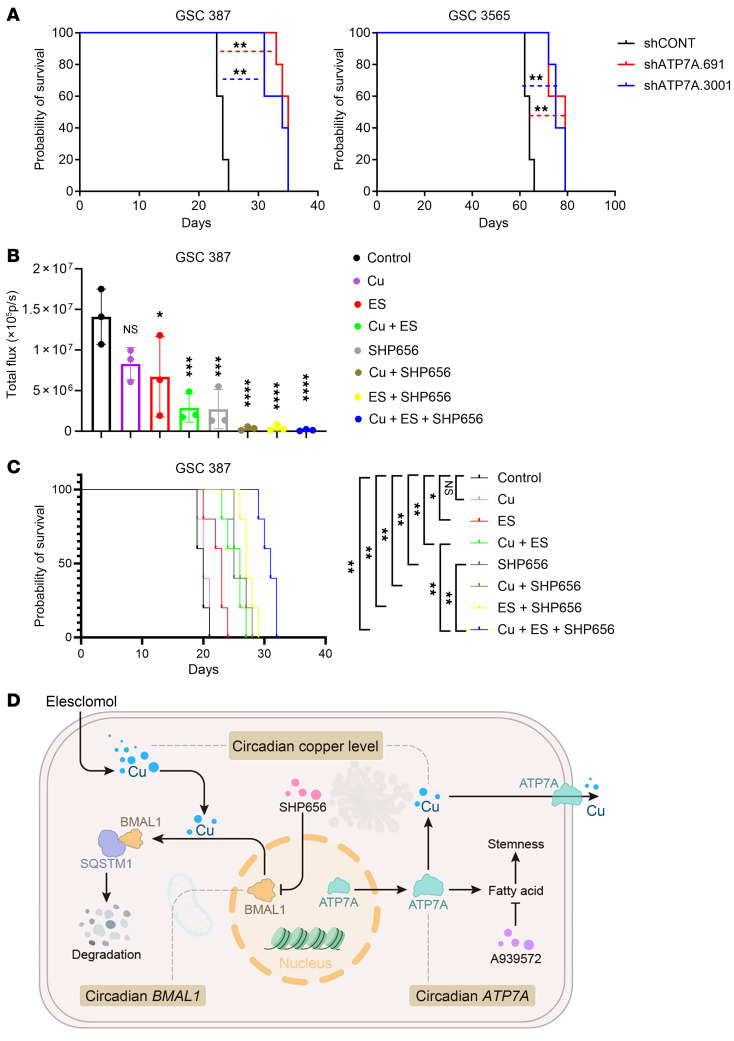
Targeting circadian clock-ATP7A-cuproptosis function in vivo. (**A**) Kaplan-Meier curves show survival of mice with GSC387 or GSC3565 tumors ± ATP7A knockdown (*n* = 5/group), analyzed by log-rank test. (**B** and **C**) Mice with intracranial GSC387 tumors received copper gluconate, ES, SHP656, or combination treatments. In vivo bioluminescence imaging was conducted, and the total flux was quantified (*n* = 3 biologically independent mice). p/s, photons/second. (**C**) The survival of the specified mice was illustrated via Kaplan-Meier curve analysis, with a sample size of *n* = 5 per group. (**D**) Schematic of this study. Log-rank tests for **A** and **C**. One-way ANOVA was performed for **B**. *P < 0.05, **P < 0.01, ***P < 0.001, ****P < 0.0001.

The gold standard for assessing cancer stem cell function is in vivo tumor initiation. We transduced luciferase-expressing GSCs with shCONT or shATP7A and implanted them intracranially into immunocompromised mice. ATP7A knockdown prolonged survival and reduced tumor burden compared with controls (Figure 8, A and B, and Supplemental Figure 10A). To evaluate ATP7A’s role in glioma stemness in vivo, intracranial limiting dilution assays (10,000–100 cells) showed that ATP7A knockdown extended survival, indicating reduced tumor initiation (Supplemental Figure 10B).

To test therapeutic potential, we evaluated the in vivo effects of the CRY stabilizer SHP656 alone or combined with copper gluconate, ES, or both. We previously reported the intracranial antitumor activity of SHP656 (23), and ES has shown monotherapy effects against GBM in vivo (30), supporting their potential for intracranial antitumor activity; however, further studies are necessary to definitively establish blood–brain barrier penetrability. Orthotopic GSC tumor growth was reduced by copper gluconate plus ES or SHP656 alone, and SHP656 further enhanced the efficacy of copper gluconate, ES, or their combination (Figure 8B and Supplemental Figure 10, C and D). Combined SHP656, ES, and copper gluconate treatment prolonged survival compared with single therapies (Figure 8C and Supplemental Figure 10E). Previous reports link GSC CLOCK/BMAL1 signaling to glioma immunosuppression (31–33). Using the CT2A syngeneic glioma model in C57BL/6 mice, combination therapy extended survival compared with single agents (Supplemental Figure 10F). Neither single-agent treatment nor combination therapy altered the proportion of CD206^+^ microglia (Supplemental Figure 10G). These results indicate that SHP656, copper, and ES act independently of microglia-mediated immunosuppression and that cotargeting the circadian clock and cuproptosis enhances therapeutic efficacy. Together, circadian control of intracellular copper supports GSC resistance to cuproptosis by coordinating BMAL1-dependent copper homeostasis, ATP7A-driven fatty acid desaturation, and copper-mediated BMAL1 degradation, revealing a targetable circadian-copper vulnerability in GBM (Figure 8D).

## Discussion

Metals are double-edged contributors to cellular physiology. Essential trace metals like iron, cobalt, copper, and zinc are required for homeostasis but harmful in excess. Elevated iron or copper triggers ferroptosis or cuproptosis, respectively. Although pharmacologic inducers of these pathways have been explored as cancer therapies, clinical efficacy remains limited. A trial in recurrent GBM comparing DSF + copper + chemotherapy versus chemotherapy alone showed no survival benefit (12). Here, we investigated mechanisms of GSC resistance to copper-induced cell death to identify strategies for overcoming this resistance.

To investigate the clinical failure of cuproptosis inducers, we examined their efficacy against GSCs, which are typically chemoresistant. GSCs exhibited relative resistance to cuproptosis, linked to circadian oscillations of intracellular copper, unlike iron — which is consistently elevated in GSCs because of high transferrin receptor and ferritin expression promoting proliferation (4). Metal analysis showed consistently elevated iron levels across tumor cells, whereas copper exhibited circadian fluctuations only in synchronized GSCs, suggesting that rhythmic copper regulation supports cancer stem cell maintenance.

As excess intracellular copper can induce oxidative stress and impair cellular function, copper homeostasis is tightly regulated (34). Our CRISPR loss-of-function screen identified the copper transporter ATP7A as essential for GSC survival under disrupted copper or circadian regulation. ATP7A dynamically relocates according to copper levels, moving to the plasma membrane under excess copper to export it and prevent toxicity (24). Under normal- or low-copper conditions, ATP7A localizes to the trans-Golgi network, supplying copper to the secretory pathway for enzyme activation. Copper-dependent enzymes, such as cytochrome *c* oxidase, superoxide dismutase, and lysyl oxidase (LOX), are key cancer modulators. Elevated ATP7A expression protects KRAS-mutant colorectal cancer cells from copper toxicity (35), whereas ATP7A silencing reduces LOX activity and inhibits lung cancer metastasis in mice (36). Fluctuations in copper availability may drive rhythmic activity of these enzymes, suggesting that cellular responses to copper depend on both its levels and its temporal dynamics.

Cuproplasia refers to the pro-proliferative effects of copper, which enhances tumor growth through pathways such as invasion, angiogenesis, and immune modulation. Conversely, cuproptosis occurs when excess copper induces nonapoptotic cell death (8). Although copper ionophores show antitumor activity, we found that GSCs are relatively resistant to cuproptosis despite comparable total copper levels, suggesting that this resistance arises from dynamic regulation of copper rather than its absolute amount.

The circadian clock regulates cellular and organismal adaptation to environmental cues (37). GBM entrains to the circadian circuit of the brain, modulating its growth through clock-controlled cues, like glucocorticoids (38). The core clock factor BMAL1 influences drug resistance in multiple cancers, enhancing paclitaxel sensitivity in tongue carcinoma (39). CRISPR screening identified *ATP7A* as a key regulator linking circadian control and cuproptosis resistance in GSCs. BMAL1 bound the *ATP7A* promoter in GSCs but not NSCs, at canonical E-box motifs (CACGTG). BMAL1 loss or pharmacologic inhibition reduced ATP7A expression and disrupted circadian rhythms of ATP7A and copper levels. Thus, BMAL1 likely regulates copper transporters, such as ATP7A, to maintain rhythmic copper homeostasis and prevent cuproptosis in GSCs.

A conserved feature across organisms is the generation of molecular circadian rhythms via negative feedback regulation (40). Although poorly defined in mammals, copper has been shown to regulate circadian components in plants, such as Arabidopsis (41). Acute copper exposure abolishes the rhythmicity of clock genes in *Danio rerio* (42). Although the autophagic degradation of BMAL1 has already been reported in the liver (43), the exact mechanism remains unclear. We found that copper feeds back on the circadian clock through SQSTM1-mediated lysosomal degradation, establishing reciprocal negative feedback between copper and circadian regulation. SQSTM1, a classical autophagy receptor, also functions in proteasomal turnover, metabolism, and apoptosis. SQSTM1 mediates aggrephagy, mitophagy, and lipophagy. BMAL1 stability is regulated by posttranslational modifications; its SUMOylation promotes ubiquitination and degradation (44). O-GlcNAcylation stabilizes BMAL1 and CLOCK by blocking phosphorylation-dependent ubiquitination and degradation (45). We identified SQSTM1 as a key mediator of copper-induced BMAL1 degradation. Copper promotes SQSTM1 oligomerization and binding to BMAL1, leading to autophagy–lysosomal degradation. This SQSTM1-mediated BMAL1 degradation exhibits rhythmic oscillation. Under lysosomal inhibition with chloroquine, SQSTM1–BMAL1 colocalization showed rhythmic patterns that were further enhanced by copper, suggesting a feedback mechanism through which copper modulates circadian rhythm to maintain homeostasis in the circadian–copper network.

Fatty acid metabolism drives growth and progression of cancers, including GBM (46). GSC-specific superenhancers drive PUFA synthesis, sustaining EGFR signaling and GSC growth (47). Lipid saturation, including transitioning between MUFAs and SFAs, is dynamically regulated in cellular metabolism (48). As SCD activity promotes GBM growth (49), SCD inhibitors have shown preclinical efficacy in neuro-oncology, but resistance arises via FOSB-mediated SCD activity (50). Here, we build on the connection between copper metabolism and fatty acid synthesis (8). Targeting ATP7A, a link between circadian regulation and cuproptosis, reduced *SCD1* expression and the MUFA/SFA ratio, indicating decreased fatty acid desaturation. While the mechanism remains unclear, several metabolic enzymes depend on copper as a cofactor. Combining a cuproptosis inducer with an SCD1 inhibitor enhanced GSC cytotoxicity, suggesting that fatty acids contribute to copper dependence. As ATP7A expression exhibits circadian oscillation in synchronized GSCs, fatty acid metabolism, particularly SCD1-mediated desaturation, may also be temporally regulated. Future studies will determine whether circadian fluctuations in ATP7A drive rhythmic changes in lipid metabolism and MUFA/SFA ratios, influencing membrane dynamics, signaling, and therapeutic sensitivity across circadian phases.

Although tumor cells are generally unsynchronized, circadian fluctuations in copper levels suggest that timing cuproptosis induction to coincide with peak copper levels may enhance efficacy. Copper’s role in cancer has driven the development of pharmacologic modulators — including chelators, ionophores, and copper complexes — as well as copper radioisotopes for tumor imaging. In vivo dosing of ES, SH656, and copper was guided by prior mouse pharmacokinetic and efficacy data; future studies will determine whether similar concentrations can be achieved safely in the human brain. Human translation will depend on factors such as blood–brain barrier permeability, metabolism, and systemic exposure, emphasizing the need for optimized delivery approaches like nanocarriers or convection-enhanced delivery. Additional preclinical work, including dose–response, brain distribution, and toxicity analyses, will guide clinical development. Copper modulators have shown tolerability but limited benefit when combined with chemotherapy such as temozolomide in GBM, and while copper ionophores exhibit strong antitumor activity by exploiting copper dependency, excessive copper remains toxic to normal cells (51). Several copper modulators, including ionophores, have been clinically evaluated for cancer and other diseases, showing manageable toxicity profiles (52). Precise dosing and targeted delivery may reduce off-target effects and optimize efficacy. Combining copper ionophores with circadian disruptors may offer greater therapeutic benefit. As therapy sensitivity varies with time of day, circadian scheduling of copper modulation and standard treatments could improve outcomes. Misalignment between drug timing and copper transporter expression may underlie prior clinical failures. Future studies profiling temporal dynamics of copper transporters like *ATP7A* could guide chronotherapy to maximize tumor susceptibility. Given copper’s roles in angiogenesis, invasion, and immune regulation, combining copper modulation with antiangiogenic therapies or immunotherapies may enhance efficacy.

## Methods

### Sex as a biological variable.

Our study used both female and male immunodeficient NSG (NOD.Cg-Prkdcscid Il2rgtm1Wjl/SzJ) mice (IMSR catalog JAX:005557, RRID: IMSR_JAX:005557, The Jackson Laboratory), and similar findings are reported for both sexes.

### Derivation and cultivation of GSCs and other cell models.

GBM tissues from consented patients at Duke University or University Hospitals Cleveland Medical Center (IRB 090401) were used to isolate and validate GSCs. ENStem-A are human embryonic stem cell–derived neural progenitors (MilliporeSigma). NSC11 are NSCs derived from human induced pluripotent stem cells (ALSTEM). hNP1s are NSCs derived from human induced pluripotent stem cells originating from the hESC WA09 line. Cell identity was confirmed by short tandem repeat profiling and mycoplasma PCR. GSCs/NSCs were cultured in Neurobasal medium (Gibco) with B27 minus vitamin A, pyruvate, GlutaMAX (Gibco), and 20 ng/mL EGF/basic FGF. DGCs were derived from GSCs in DMEM + 10% FBS for at least 7 days.

### Tumor xenografts.

All mouse experiments followed protocols approved by the University of Pittsburgh IACUC. For intracranial xenografts, 4- to 6-week-old NSG or C57BL/6J mice were implanted with 10^5^ patient-derived GSCs into the right cortex (3.5 mm depth). Animals were housed under veterinary supervision and euthanized upon neurological decline. Brains were fixed in 4% paraformaldehyde, paraffin-embedded, and analyzed by H&E staining. Survival was assessed using GraphPad Prism (log-rank test). For luciferase-labeled GSCs, mice received d-luciferin (50 mg/kg, i.p.; Promega) and were imaged under isoflurane anesthesia with an IVIS system (PerkinElmer). For in vivo studies, mice were similarly implanted intracranially. After 7 days, the mice were subjected to treatment with vehicle, ES (25 mg/kg, intragastric administration [i.g.], once every other day) (30), copper gluconate (0.15 mg/kg, i.g., once every day) (53), or SHP656 (10 mg/kg, i.g., twice a day) (14) until reaching specified endpoints.

### Flow cytometry.

Tumor-bearing brains were microdissected, and tumors were dissociated into single cells using Collagenase D (2 mg/mL; Roche) for 30 minutes at 37°C. RBCs were removed with lysis buffer (Thermo Fisher Scientific) for 3 minutes and quenched with medium. Cells were stained with fluorochrome-conjugated antibodies against CX3CR1, CD11b, CD45, and CD206 (BioLegend) for 30 minutes at 4°C. Data were collected on an Attune flow cytometer and analyzed with FlowJo (BD Biosciences). Microglia were defined as CD45^lo^CD11b^+^CX3CR1^+^ cells.

### CRISPR knockout library.

A total of 636 sgRNA oligonucleotides targeting 58 genes involved in copper regulation (Supplemental Table 1) were synthesized by VectorBuilder (Lib230221-1474xzd). GSC3565 cells stably expressing Cas9 (lentiCas9-Blast, Addgene #52962) were transduced with the pooled lentiviral library at MOI 0.3 (>1,000× coverage, 2 replicates). After 2 days, puromycin selection was applied. On day 5, cells were collected as the day 1 baseline. Remaining cells were divided into 3 groups, SHP656 (8.5 μM), ES (35 μM) + CuCl_2_ (1 μM), or untreated control, and cultured 14 days. Comparison of day 14 versus day 1 controls identified fitness genes; SHP656-treated versus untreated identified circadian drug resistance genes; and ES-treated versus untreated identified cuproptosis resistance genes. Genomic DNA was extracted (QIAGEN), PCR-amplified (primers in Supplemental Table 8), and sequenced (Illumina PE150, CD Genomics). Data were processed and analyzed using MAGeCK on Galaxy (RRID:SCR_006281), with *P* < 0.05 defining significant hits (Supplemental Tables 2 and 3).

### Plasmids and cloning.

shRNAs used were shBMAL1 (TRCN0000019097, TRCN0000019096), shATP7A (TRCN0000043173, TRCN0000043177, TRCN0000418612), shSQSTM1 (TRCN0000007237), and shATG7 (TRCN0000007584). A nontargeting shRNA (Sigma-Aldrich, SCH002) served as control. ATP7A- and BMAL1-overexpression plasmids were from VectorBuilder (VB220919-1171bzh, VB221011-1324hfh). FLAG-SQSTM1-WT and FLAG-SQSTM1 fragment plasmids that lacked specific domain were constructed according to Figure 5H.

### Retroviral packaging and infection.

For stable gene modulation, lentiviruses were produced by cotransfecting HEK293T cells (ATCC) with transfer plasmid (3 μg), psPAX2 (6.75 μg), pMD2.G (2.25 μg), and PEI (24 μL). Viral supernatants were collected at 48 hours and 72 hours, concentrated (Lenti-X, Takara), and used to transduce GSCs with 10 μg/mL polybrene for 24 hours. Infected cells were selected with 2 μg/mL puromycin for 2 days, and infection efficiency was verified by qPCR or immunoblotting.

### Cell viability measurements.

Cell viability was measured using the cell counting kit-8 (CCK-8) assay (APEXBIO, K1018) in 96-well plates seeded with 3,000 cells/well on days 0, 2, 4, and 6 per the manufacturer’s instructions.

### Single-cell RNA-Seq analysis.

Single-cell RNA-Seq data from NCBI GEO (GSE174554) were reanalyzed using Seurat. Uniform manifold approximation and projection was generated after standard preprocessing, and tumor cells were classified as stem-like or differentiated glioma via AUCell using a validated GSC gene signature (54). GOBP module scores were calculated and normalized (from 0 to 1) using Seurat functions in R.

### Drug sensitivity and synergy testing.

Cells (3,000/well) were seeded in Matrigel-coated, 96-well plates and treated with 2-fold serial drug dilutions. After 48 hours’ incubation (37°C, 5% CO_2_), CCK-8 reagent was added for 1 hour at room temperature, and luminescence was measured. Dose–response curves were generated in GraphPad Prism (RRID:SCR_002798), and drug synergy was analyzed using the R package SynergyFinder.

### EdU incorporation assay.

The Click-iT EdU assay (Thermo Fisher Scientific) was performed per manufacturer instructions. Cells were incubated with 10 μM EdU for 2 hours, and EdU-positive fractions were quantified relative to DAPI-stained nuclei using ImageJ (NIH).

### Western blotting.

Cells were lysed in RIPA buffer with protease inhibitors on ice for 15 minutes and centrifuged at 12,000*g* for 10 minutes at 4°C. Protein concentrations were measured by BCA assay. Equal protein amounts were mixed with 4× lithium dodecyl sulfate buffer, boiled for 5 minutes, and used for NuPAGE or stored at –80°C. PVDF membranes were blocked with 5% milk in TBS-Tween for 1 hour and incubated overnight with primary antibodies at 4°C. Antibodies used in this study are detailed in Supplemental Table 9. Membranes were incubated with HRP-conjugated secondary antibodies, developed using Immobilon ECL Ultra substrate (MilliporeSigma), and imaged with a Bio-Rad ChemiDoc MP system.

### Co-IP.

Cells were lysed in IP buffer (Thermo Fisher Scientific, 87788) with protease inhibitors, and cleared lysates were incubated overnight at 4°C with target or control IgG antibodies. Pierce Protein A/G Magnetic Beads (88802, Thermo Fisher Scientific) were added for 2 hours at 4°C, washed 4 times with IP buffer, and then boiled and analyzed by immunoblotting.

### RNA extraction and quantitative RT-PCR.

Total RNA was extracted using TRIzol (Sigma-Aldrich) per manufacturer instructions. cDNA was synthesized with the High-Capacity Reverse Transcription Kit (Thermo Fisher Scientific, 4368814), and qPCR was performed using SYBR Green Master Mix (Thermo Fisher Scientific, 4309155) on a CFX Connect Real-Time PCR System (Bio-Rad). β-Actin served as control; primer sequences are in Supplemental Table 8.

### ChIP-qPCR.

Cells were cross-linked with 1% formaldehyde for 10 minutes and quenched with 0.125 M glycine. Nuclei were lysed and sonicated to yield 200–500 bp chromatin fragments, which were incubated overnight at 4°C with anti-BMAL1 (CST 14020S) or control IgG. Immunocomplexes were captured with Protein A/G magnetic beads, washed, eluted, and reverse cross-linked. The purified DNA was analyzed by PCR.

### Immunofluorescence.

Cells grown on Matrigel-coated coverslips were fixed with 4% paraformaldehyde for 15 minutes, permeabilized with 0.1% Triton X-100 for 10 minutes, and blocked in serum for 1 hour. Cells were then incubated with primary antibodies overnight at 4°C, followed by secondary antibodies. Nuclei were counterstained with DAPI, and slides were mounted with ProLong Diamond Antifade Mountant (Thermo Fisher Scientific). All antibodies used in this study are detailed in Supplemental Table 9.

### Copper live-cell imaging.

Live-cell copper imaging was performed using BioTracker Green Copper Live Cell Dye (SCT041, MilliporeSigma) according to the manufacturer’s instructions. Briefly, cells cultured on Matrigel-coated dishes were incubated with 5 μM dye for 2 hours, rinsed twice with observation buffer, and imaged using a Leica SP8 microscope.

### Protein stability assays.

Cells were treated with CHX (100 μg/mL; Sigma-Aldrich), chloroquine (10 μM; Selleck, S4157), or MG132 (10 μM; MedChemExpress, HY-13259) for the indicated times before Western blotting.

### RNA-Seq.

For RNA-Seq following ATP7A knockdown, total RNA was extracted from GSC387, GSC3565, and GSC738 cells transduced with shCONT or shATP7A using TRIzol (Invitrogen) and Direct-zol RNA Miniprep Kits (Zymo Research). Purified RNA was eluted in RNase-free water and subjected to paired-end 150 bp sequencing. Raw reads were trimmed with Trim Galore and aligned to the human genome (hg38) using HISAT2. Samtools was used for sorting, indexing, and file conversion, and gene quantification and differential expression analysis were performed with FeatureCounts and DESeq2 (paired design). Differentially expressed genes (fold-change > 1, *P* < 0.01) were analyzed for pathway enrichment (KEGG, Reactome) and GSEA using Omicshare tools. RNA-Seq data are available in GEO (GSE278671).

### ICP-MS.

Metal quantification was performed by Sarah Jantzi at the Plasma Chemistry Laboratory, University of Georgia. Samples were digested in polytetrafluoroethylene vessels with 0.5 mL trace metal–grade nitric acid and 0.5 mL hydrogen peroxide (1 hour each at 95°C), diluted to 2% HNO_3_, and analyzed by ICP-MS (Thermo X-Series 2) using an indium internal standard in kinetic energy discrimination mode with 8% H_2_/He to reduce interference.

### Lipidomics.

Lipidomics analysis was performed by the West Coast Metabolomics Center, UC Davis Genome Center. Frozen cell pellets were processed using the Matyash extraction method (MTBE/MeOH/H_2_O). The organic phase was dried, resuspended in 9:1 methanol/toluene containing 50 ng/mL CUDA, and centrifuged (14,000*g*). Aliquots were prepared for positive- and negative-mode analyses on an Agilent 1290 Infinity LC system coupled to an Agilent 6546 QTOF mass spectrometer. Samples were separated on an Acquity Premier BEH C18 column (1.7 μm, 2.1 × 50 mm; Waters) using a 5.5-minute gradient (15%–99% B) at 0.8 mL/min. Positive-mode scans covered *m/z* 120–1,200 Da; negative-mode scans, *m/z* 60–1,200 Da. Mass resolutions were 10,000 (electrospray ionization^+^) and 30,000 (electrospray ionization^–^). Lipidomics data are provided in Supplemental Table 4.

### GSC dataset interrogation and transcription regulator analysis.

Single-sample GSEA of GOBP pathways was performed using the GSVA package (ssgsea method) in R. Circadian rhythm (GO:0007623) and copper homeostasis (GO:0006878) signatures from the GO database were used to infer pathway activity. Ten cuproptosis-related genes (8) (*FDX1*, *LIAS*, *LIPT1*, *DLD*, *DLAT*, *PDHA1*, *PDHB*, *MTF1*, *GLS*, and *CDKN2A*) were used to construct a cuproptosis signature. RNA-Seq data of 44 GSCs and 9 NSCs were from GSE119834 (22) and were analyzed using the limma package. ChIP-Seq data of BMAL1 from GSCs and NSCs were from GSE134972 (14). ChIP-Seq data of H3K27ac were from GSE54047 (55) and GSE119755 (22). Integrative Genomics Viewer (RRID:SCR_011793) was used for peak visualization.

### MS.

MS was performed by the Proteomics Resource Center at The Rockefeller University. Nuclear BMAL1-interacting proteins were immunoprecipitated from GSC3565 cells and eluted by partial on-bead digestion with 300 ng trypsin (Promega) in 50 mM ammonium bicarbonate for 3 hours at room temperature. Supernatants were reduced with 10 mM DTT, alkylated with 100 mM iodoacetamide for 1 hour in the dark, and further digested overnight with 500 ng trypsin and 500 ng Lys-C (Wako) in 50 mM ammonium bicarbonate. Digestion was stopped with trifluoroacetic acid. Peptides underwent reversed-phase-based micro-solid-phase extraction (56). Triplicates of 10 μL were injected and analyzed by nano–liquid chromatography-tandem mass spectrometry (Fusion LUMOS coupled to an Easy-nLC 1200, Thermo Fisher Scientific). Mass spectrometers were mass-calibrated weekly and operated with lock mass (57). MS and MS/MS spectra were acquired at resolutions of 120,000 and 30,000 (@200 Thomson), respectively, with automatic gain control targets of 4 × 10^5^ (MS) and 5 × 10^4^ (MS/MS). Peptides were separated on a 12 cm × 100 μm packed emitter column (NIKKYO TECHNOS) using a 70-minute gradient from 2% to 35% solvent B (A: 0.1% formic acid; B: 80% acetonitrile/0.1% formic acid). Peptide counts are listed in Supplemental Table 4. Proteins with IgG/IP-BMAL peptide ratios less than 0.3 were defined as BMAL1-binding proteins (Supplemental Table 5). GOBP:SELECTIVE_AUTOPHAGY, GO0061912 (Supplemental Table 6), were considered autophagy-related proteins.

### Public human glioma datasets.

Public glioma datasets from GlioVis were used to analyze mRNA expression, correlations (*ATP7A*, *BMAL1*, *SOX2*, *OLIG2*, *SCD1*), and ATP7A survival in the CGGA cohort.

### Statistics.

A significance threshold of *P* < 0.05 was used. Rhythmicity was analyzed using the RTK (JTK_CYCLE–based) algorithm. Statistical tests, including 2-tailed unpaired t test, 1-way or 2-way ANOVA followed by Tukey’s multiple-comparison correction, Pearson’s correlation, likelihood-ratio test, and log-rank test, were performed using GraphPad Prism (RRID:SCR_002798). Data are presented as mean ± SEM unless otherwise noted.

### Study approval.

All mouse experiments were approved by the University of Pittsburgh IACUC.

### Data availability.

The data that support the findings of this study are available within the article and its supplement. Values for all data points in graphs are reported in the Supporting Data Values file. The RNA-Seq and ChIP-Seq data are deposited in the NCBI’s GEO database (GSE278671).

## Author contributions

FY performed conceptualization, data curation, formal analysis, investigation, methodology, validation, visualization, writing — original draft, writing — review and editing. XW performed data curation, formal analysis, investigation, methodology, validation, visualization. HY performed data curation, formal analysis, investigation, methodology, validation, visualization. DW performed data curation, formal analysis, investigation, methodology, validation, visualization. TH performed investigation, validation. PZ performed investigation, validation. HM performed investigation, validation. WW performed investigation, validation. ST performed investigation, validation. PC performed investigation, validation. KM performed investigation, validation. MTG performed investigation, validation. LZ performed visualization. KGA supervised. SAK supervised. QW performed investigation, project administration, resources, validation. JNR performed conceptualization, data curation, formal analysis, funding acquisition, methodology, project administration, resources, supervision, visualization, writing — original draft, and writing — review and editing.

## Funding support

This work is the result of NIH funding, in whole or in part, and is subject to the NIH Public Access Policy. Through acceptance of this federal funding, the NIH has been given a right to make the work publicly available in PubMed Central.

- NIH grants (CA197718, CA238662, CA268634, NS136424, NS103434, and NS134724 to JNR).
- Defense Health Agency (HT9425-23-1-0689 to JNR).
- American Cancer Society Lisa Dean Moseley Foundation Cancer Stem Cell Consortium (to JNR).

## Supplementary Material

Supplemental data

Unedited blot and gel images

Supplemental tables 1-9

Supporting data values

## Version 1 01/02/2026

Electronic publication

## Version 2 02/24/2026

affiliations for Linjie Zhao updated
