# Supplementary material for: Glioblastoma stem cells resist cuproptosis with circadian variation of copper levels
Source: J Clin Invest. 2026 Jan 2;136(1):e192599. doi: 10.1172/JCI192599 (PMC12721906; doi:10.1172/JCI192599)
Supplement: Supplemental data [file jci-136-192599-s009.pdf]

**Supplemental Figure 1.**

(A) Cell viability after elesclomol treatment with or without 1 μM of the indicated metals. (B) Western blots of DLAT, LIAS, and FDX1 in GSCs treated with elesclomol ± 1 μM $CuCl_2$; band intensities quantified by ImageJ and normalized to β-actin. One-way ANOVA with multiple comparisons. (C) Representative images of GSC sphere formation under indicated elesclomol concentrations (1 μM $CuCl_2$). Scale bar: 100 μm. (D) Quantification of Figure 1D; relative intensities normalized to β-actin, analyzed by one-way ANOVA. ****p<0.0001; ***p<0.001; **p<0.01; *p<0.05; ns, not significant.

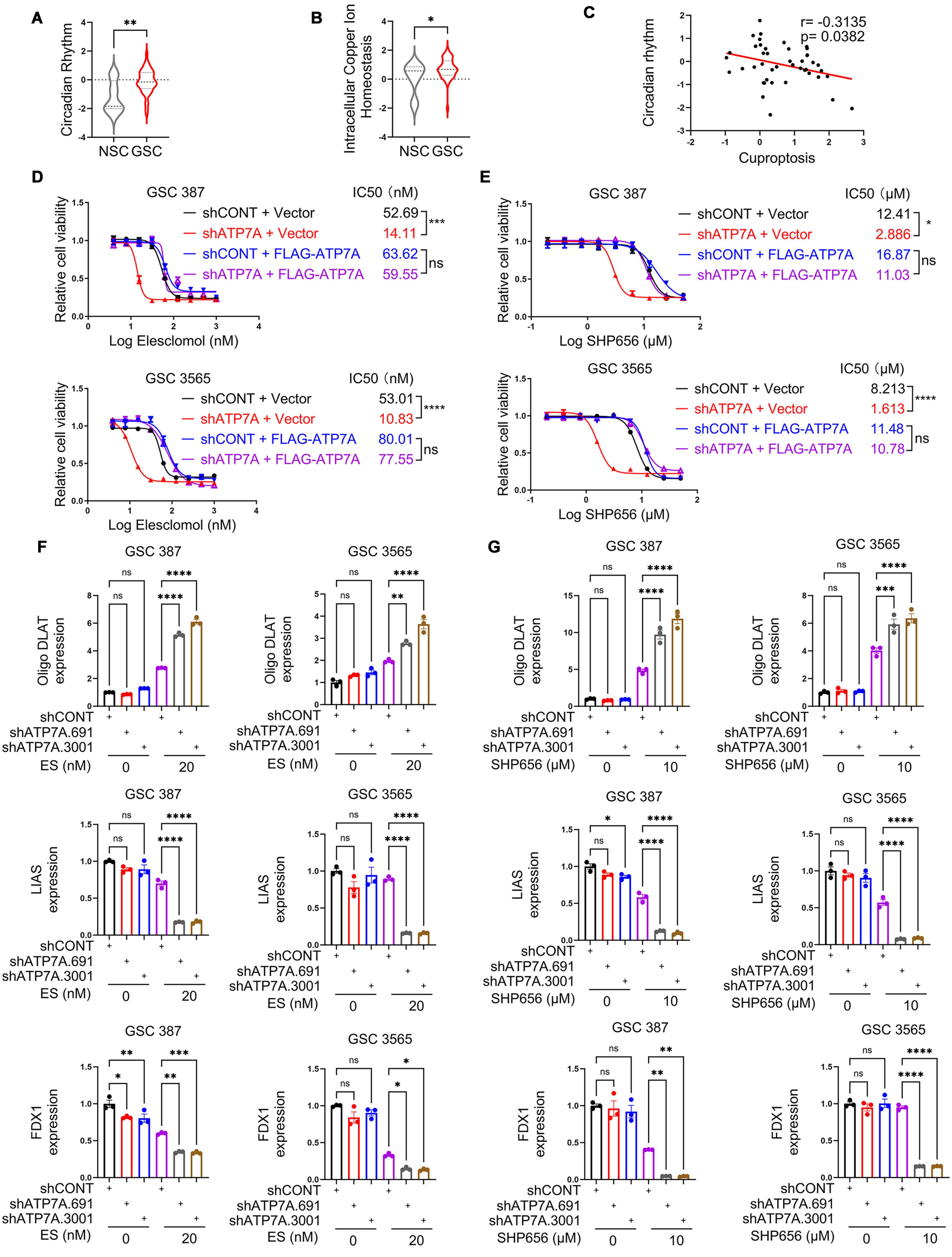

A
Circadian Rhythm
NSC GSC
B
Intracellular Copper Ion Homeostasis
NSC GSC
C
r= -0.3135
p= 0.0382
Circadian rhythm
Cuproptosis
D
GSC 387
IC50 (nM)
shCONT + Vector 52.69
shATP7A + Vector 14.11
shCONT + FLAG-ATP7A 63.62
shATP7A + FLAG-ATP7A 59.55
Relative cell viability
Log Elesclomol (nM)
GSC 3565
IC50 (nM)
shCONT + Vector 53.01
shATP7A + Vector 10.83
shCONT + FLAG-ATP7A 80.01
shATP7A + FLAG-ATP7A 77.55
E
GSC 387
IC50 (μM)
shCONT + Vector 12.41
shATP7A + Vector 2.886
shCONT + FLAG-ATP7A 16.87
shATP7A + FLAG-ATP7A 11.03
Log SHP656 (μM)
GSC 3565
IC50 (μM)
shCONT + Vector 8.213
shATP7A + Vector 1.613
shCONT + FLAG-ATP7A 11.48
shATP7A + FLAG-ATP7A 10.78
F
G
GSC 387
GSC 3565
Oligo DLAT expression
LIAS expression
FDX1 expression
shCONT
shATP7A.691
shATP7A.3001
ES (nM) 0 20
SHP656 (μM) 0 10

**Supplemental Figure 2.**

(A and B) ssGSEA of circadian rhythm (GO:0007623) and copper homeostasis (GO:0006878) gene sets in 44 GSCs and 10 NSCs (GSE119834). Unpaired t-test used for comparison. (C) Correlation between circadian rhythm and cuproptosis signatures (10 genes: FDX1, LIAS, LIPT1, DLD, DLAT, PDHA1, PDHB, MTF1, GLS, CDKN2A); Pearson correlation with linear regression. (D and E) $IC_{50}$ and dose–response curves of elesclomol (ES) and SHP656 in GSC387 and GSC3565 expressing shATP7A (UTR-targeting) ± shRNA-resistant FLAG-ATP7A or vector control (1 μM $CuCl_2$, 48 h). (F and G) Quantification of Figures 3F and G; band intensities normalized to β-actin via ImageJ. One-way ANOVA followed by multiple comparisons for D-G. ****p<0.0001; ***p<0.001; **p<0.01; *p<0.05; ns, not significant.

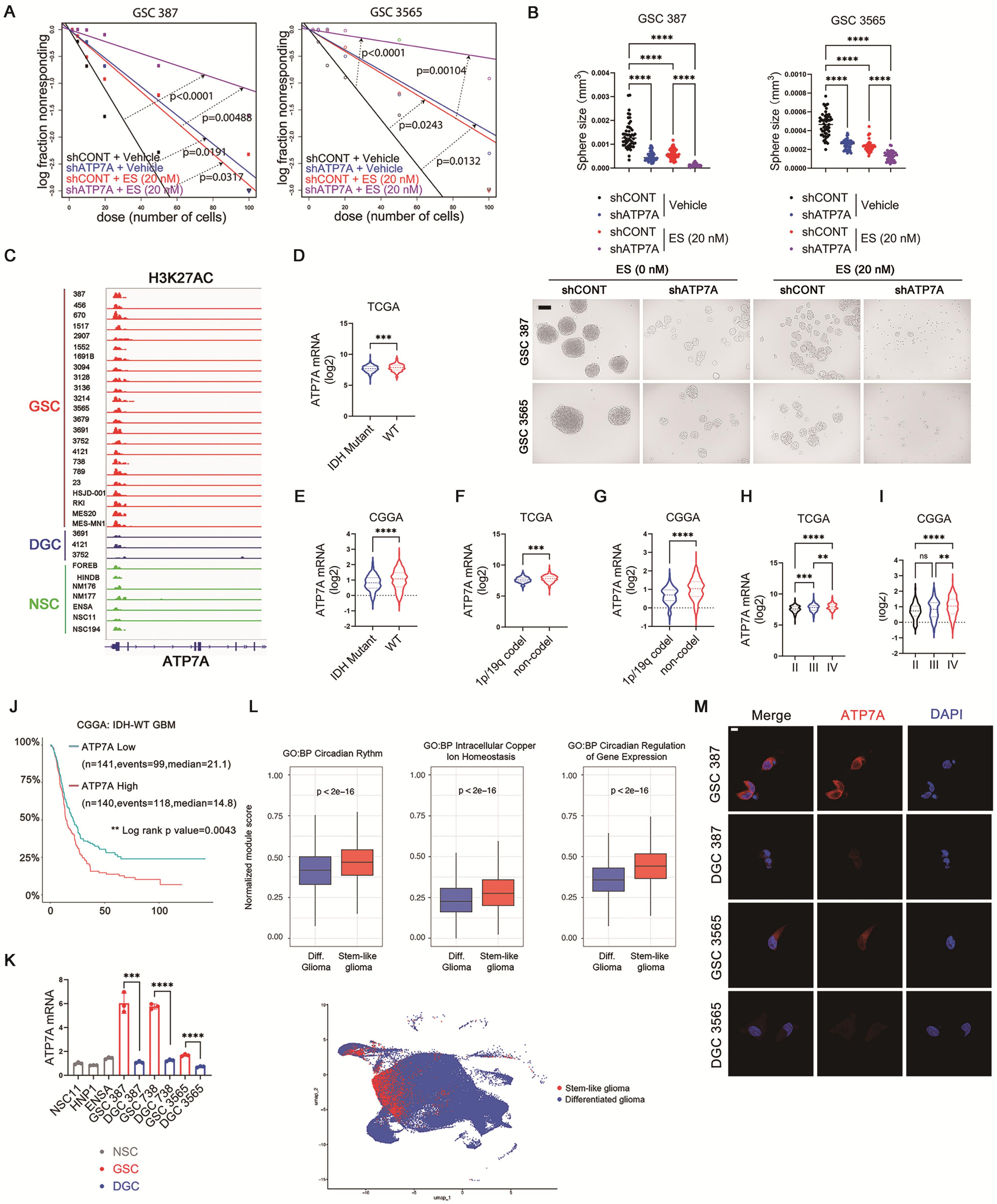
A
GSC 387
GSC 3565
log fraction nonresponding
dose (number of cells)
shCONT + Vehicle
shATP7A + Vehicle
shCONT + ES (20 nM)
shATP7A + ES (20 nM)
p<0.0001
p=0.00488
p=0.0191
p=0.0317
p=0.00104
p=0.0243
p=0.0132
B
Sphere size (mm3)
shCONT
shATP7A
Vehicle
ES (20 nM)
ES (0 nM)
C
H3K27AC
GSC
DGC
NSC
ATP7A
D
TCGA
ATP7A mRNA (log2)
IDH Mutant
WT
E
CGGA
F
1p/19q codel
non-codel
G
H
I
II
III
IV
ns
J
CGGA: IDH-WT GBM
ATP7A Low
(n=141,events=99,median=21.1)
ATP7A High
(n=140,events=118,median=14.8)
** Log rank p value=0.0043
K
ATP7A mRNA
NSC11
HNP1
ENSA
GSC 387
DGC 387
GSC 738
DGC 738
GSC 3565
DGC 3565
NSC
GSC
DGC
L
GO:BP Circadian Rythm
GO:BP Intracellular Copper Ion Homeostasis
GO:BP Circadian Regulation of Gene Expression
Normalized module score
p < 2e−16
Diff. Glioma
Stem-like glioma
Stem-like glioma
Differentiated glioma
umap_1
umap_2
M
Merge
ATP7A
DAPI
GSC 387
DGC 387
GSC 3565
DGC 3565

**Supplemental Figure 3.**

(A) Extreme limiting dilution and (B) sphere formation assays of control and ATP7A-knockdown GSCs treated with elesclomol (ES, 0–20 nM, 1 µM $CuCl_2$). Representative images shown; scale bar: 100 µm. (C) H3K27ac signal at the ATP7A locus across 23 GSCs, 3 DGCs, and 7 normal brain (NB) cells. (D–I) ATP7A mRNA expression in glioma subgroups from TCGA and CGGA: IDH status, 1p/19q codeletion, and WHO grades II–IV. (J) Kaplan–Meier survival of IDH-WT GBM patients (CGGA) stratified by median ATP7A expression. (K) qPCR of ATP7A in GSCs, DGCs, and NSCs (normalized to NSC11 = 1; mean ± SEM, n = 3). (L) Single-cell dataset (GSE174554) showing normalized module scores for circadian (GO:0007623, GO:0032922) and copper homeostasis (GO:0006878) programs in stem-like vs. differentiated glioma. (M) Immunofluorescence showing ATP7A localization in GSCs and DGCs. Scale bar: 10 µm. Two-tailed likelihood-ratio test for A. Two-tailed unpaired t test for D-G. One-way ANOVA followed by multiple comparison for B, H, I and K. Log-rank analysis for J. ****$p<0.0001$; ***$p<0.001$; **$p<0.01$; *$p<0.05$; ns, not significant.

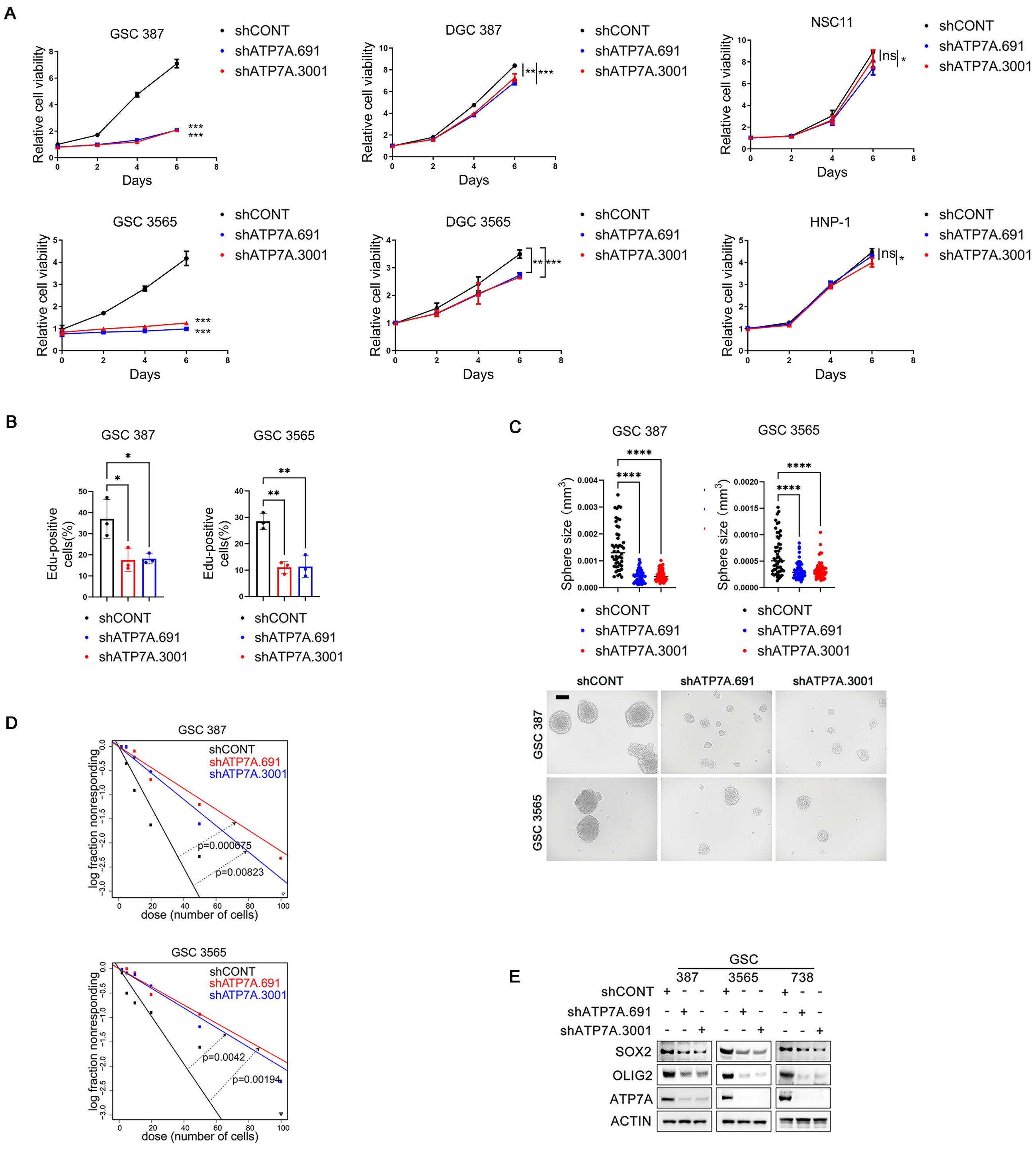


**Supplemental Figure 4**

(A) CCK-8 assay measuring viability of GSCs, DGCs, and NSCs over 6 days after ATP7A knockdown (shATP7A.691, shATP7A.3001; n=3). (B) EdU incorporation in control and ATP7A-knockdown GSCs (n=3). (C) Sphere formation and (D) extreme limiting dilution assays of GSCs with control or ATP7A knockdown. Scale bar: 100 µm. (E) Western blots showing SOX2 and OLIG2 expression after ATP7A knockdown.

One-way ANOVA followed by multiple comparisons for B and C. Two-way ANOVA followed by multiple comparisons for A. Two-tailed likelihood-ratio test for D

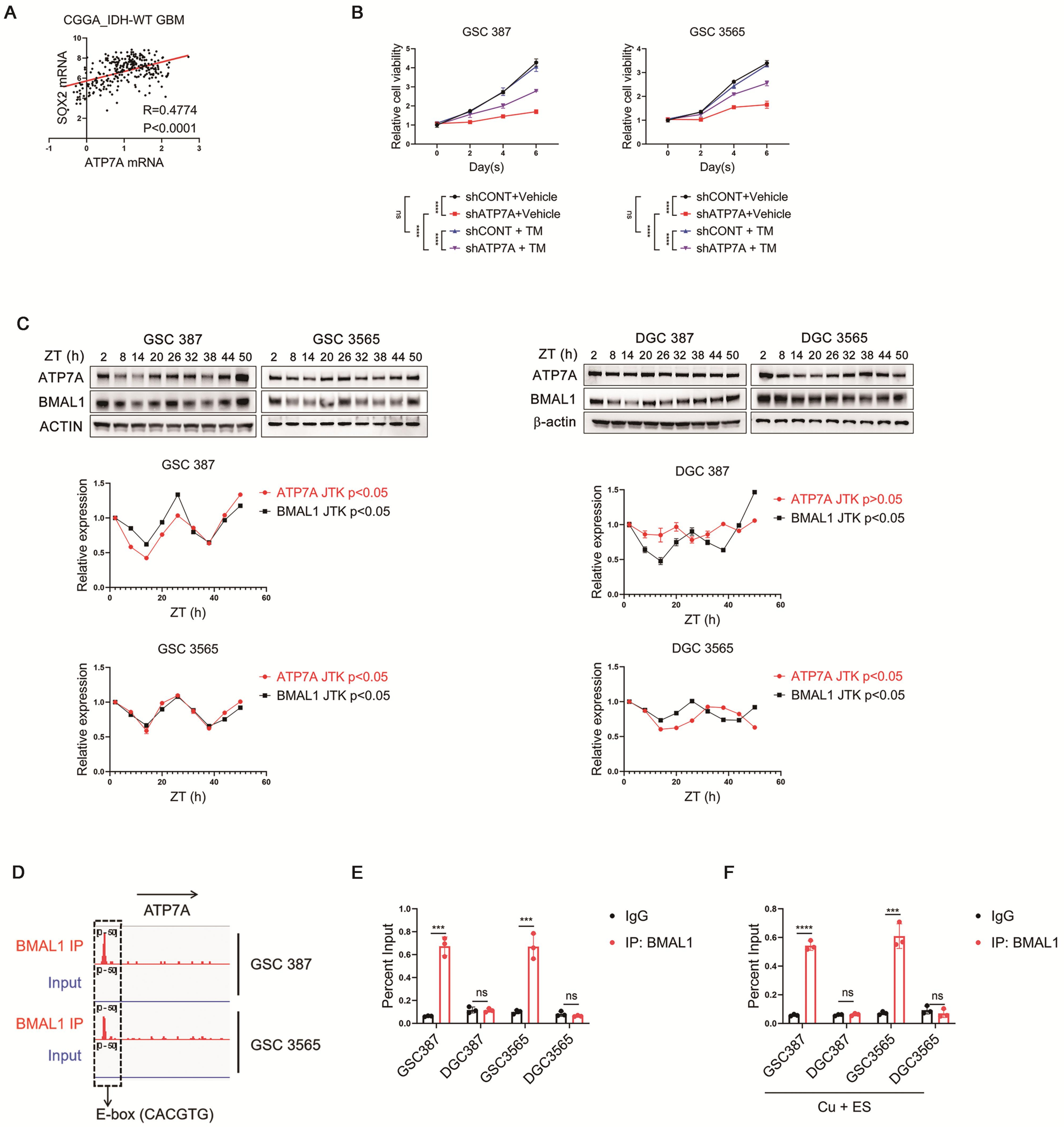


**Supplemental Figure 5**

(A) Pearson correlation between ATP7A and SOX2 expression in IDH-WT GBM (CGGA, n=287); red line indicates linear regression. (B) CCK-8 assay of GSC viability over 6 days after ATP7A knockdown ± TM treatment (n=3). (C) Time-course Western blots of ATP7A and BMAL1 at 6-h intervals in synchronized GSCs and DGCs (n=3). (D) UCSC genome tracks showing BMAL1 ChIP-seq enrichment at the ATP7A locus in GSCs. (E and F) BMAL1 ChIP-qPCR confirming ATP7A promoter binding in GSCs and DGCs (n=3). Rhythmicity analysis using the RTK algorithm in C. $P<0.05$ indicates significant rhythmicity. Two-way ANOVA followed by multiple comparisons for B. T-test for E and F. Pearson correlation analysis for A. ****$p<0.0001$; ***$p<0.001$; **$p<0.01$; *$p<0.05$; ns, not significant.

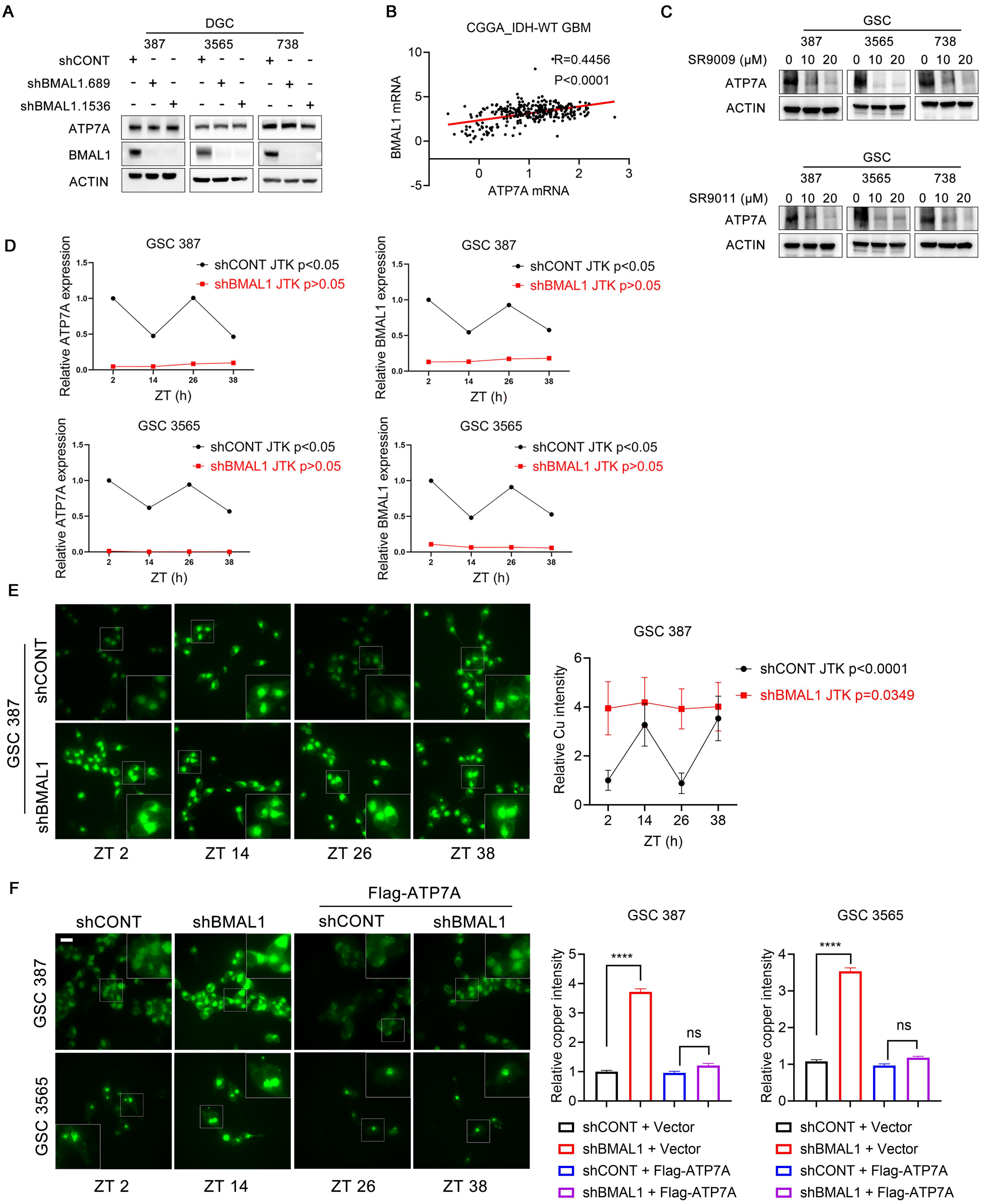


**Supplemental Figure 6.**

(A) Western blot showing ATP7A expression in DGCs after BMAL1 knockdown (shBMAL1.689, shBMAL1.1536). (B) Correlation between ATP7A and BMAL1 mRNA expression in IDH-WT GBM (CGGA, n=287; GlioVis). (C) Western blot of ATP7A in GSCs treated with circadian inhibitors SR9009 or SR9011. (D) Quantification of Figure 4F. (E) Live-cell copper imaging of synchronized GSC387-shCONT and shBMAL1 cells at 12 h intervals (100 nM dexamethasone). Scale bar: 20 μm. (F) Copper imaging in GSCs with control or BMAL1 knockdown ± ATP7A overexpression (Flag-ATP7A). Scale bar: 20 μm. Pearson correlation analysis for B. Rhythmicity analysis using the RTK algorithm in D and E. $P<0.05$ indicates significant rhythmicity. One-way ANOVA followed by multiple comparison for F.

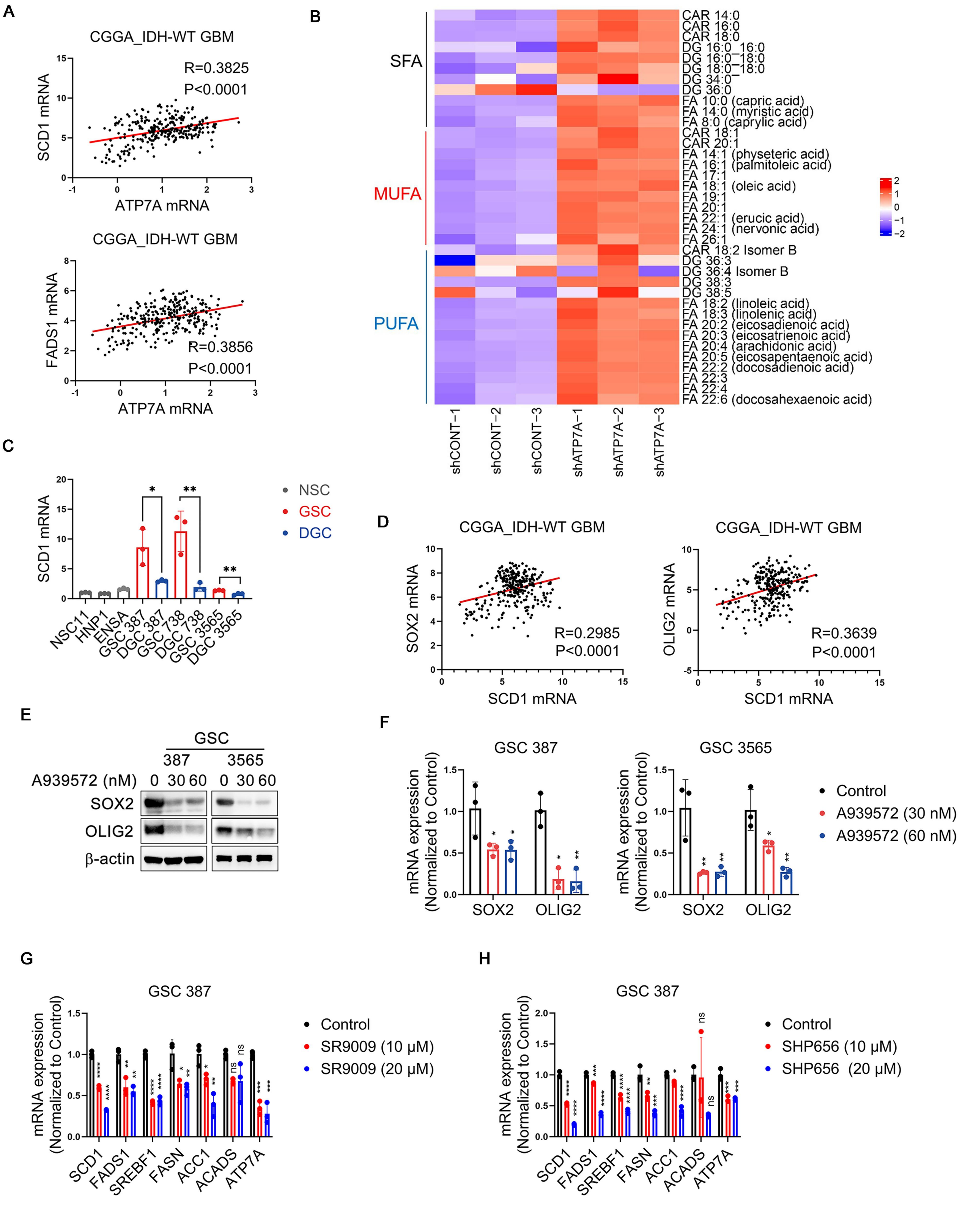

A
CGGA_IDH-WT GBM
R=0.3825
P<0.0001
SCD1 mRNA
ATP7A mRNA
CGGA_IDH-WT GBM
R=0.3856
P<0.0001
FADS1 mRNA
ATP7A mRNA
B
SFA
MUFA
PUFA
CAR 14:0
CAR 16:0
CAR 18:0
DG 16:0_16:0
DG 16:0_18:0
DG 18:0_18:0
DG 34:0
DG 36:0
FA 10:0 (capric acid)
FA 14:0 (myristic acid)
FA 8:0 (caprylic acid)
CAR 18:1
CAR 20:1
FA 14:1 (physeteric acid)
FA 16:1 (palmitoleic acid)
FA 17:1
FA 18:1 (oleic acid)
FA 19:1
FA 20:1
FA 22:1 (erucic acid)
FA 24:1 (nervonic acid)
FA 26:1
CAR 18:2 Isomer B
DG 36:3
DG 36:4 Isomer B
DG 38:3
DG 38:5
FA 18:2 (linoleic acid)
FA 18:3 (linolenic acid)
FA 20:2 (eicosadienoic acid)
FA 20:3 (eicosatrienoic acid)
FA 20:4 (arachidonic acid)
FA 20:5 (eicosapentaenoic acid)
FA 22:2 (docosadienoic acid)
FA 22:3
FA 22:4
FA 22:6 (docosahexaenoic acid)
shCONT−1
shCONT−2
shCONT−3
shATP7A−1
shATP7A−2
shATP7A−3
C
SCD1 mRNA
NSC
GSC
DGC
NSC11
HNP1
ENSA
GSC 387
DGC 387
GSC 738
DGC 738
GSC 3565
DGC 3565
D
CGGA_IDH-WT GBM
SOX2 mRNA
SCD1 mRNA
R=0.2985
P<0.0001
CGGA_IDH-WT GBM
OLIG2 mRNA
SCD1 mRNA
R=0.3639
P<0.0001
E
GSC
387
3565
A939572 (nM)
0 30 60 0 30 60
SOX2
OLIG2
β-actin
F
GSC 387
GSC 3565
mRNA expression (Normalized to Control)
SOX2
OLIG2
Control
A939572 (30 nM)
A939572 (60 nM)
G
GSC 387
mRNA expression (Normalized to Control)
Control
SR9009 (10 μM)
SR9009 (20 μM)
SCD1
FADS1
SREBF1
FASN
ACC1
ACADS
ATP7A
H
GSC 387
mRNA expression (Normalized to Control)
Control
SHP656 (10 μM)
SHP656 (20 μM)
SCD1
FADS1
SREBF1
FASN
ACC1
ACADS
ATP7A

**Supplemental Figure 7.**

(A) Pearson correlation of ATP7A, SCD1, and FADS1 expression in IDH-WT GBM (CGGA, n=287); red lines indicate linear regression (R-values shown). (B) Lipidomic heatmap of SFA, MUFA, and PUFA in GSC3565 with control or ATP7A knockdown. (C) qPCR of SCD1 in GSCs, DGCs, and NSCs (normalized to NSC11 = 1; n=3). (D) Correlation of SCD1 with SOX2 and OLIG2 expression in IDH-WT GBM (CGGA, n=287; GlioVis). (E and F) Western blot and qPCR showing SOX2 and OLIG2 expression in GSCs treated with A939572. (G and H) qPCR of fatty acid–related genes (SCD1, FADS1, SREBF1, FASN, ACC1, ACADS) and ATP7A in GSCs treated with SR9009 (G) or SHP656 (H) (n=3).

Data are mean ± SEM. One-way ANOVA for C, F–H; Pearson correlation for A, D. ****P<0.0001, ***P<0.001, **P<0.01, *P<0.05, ns not significant.

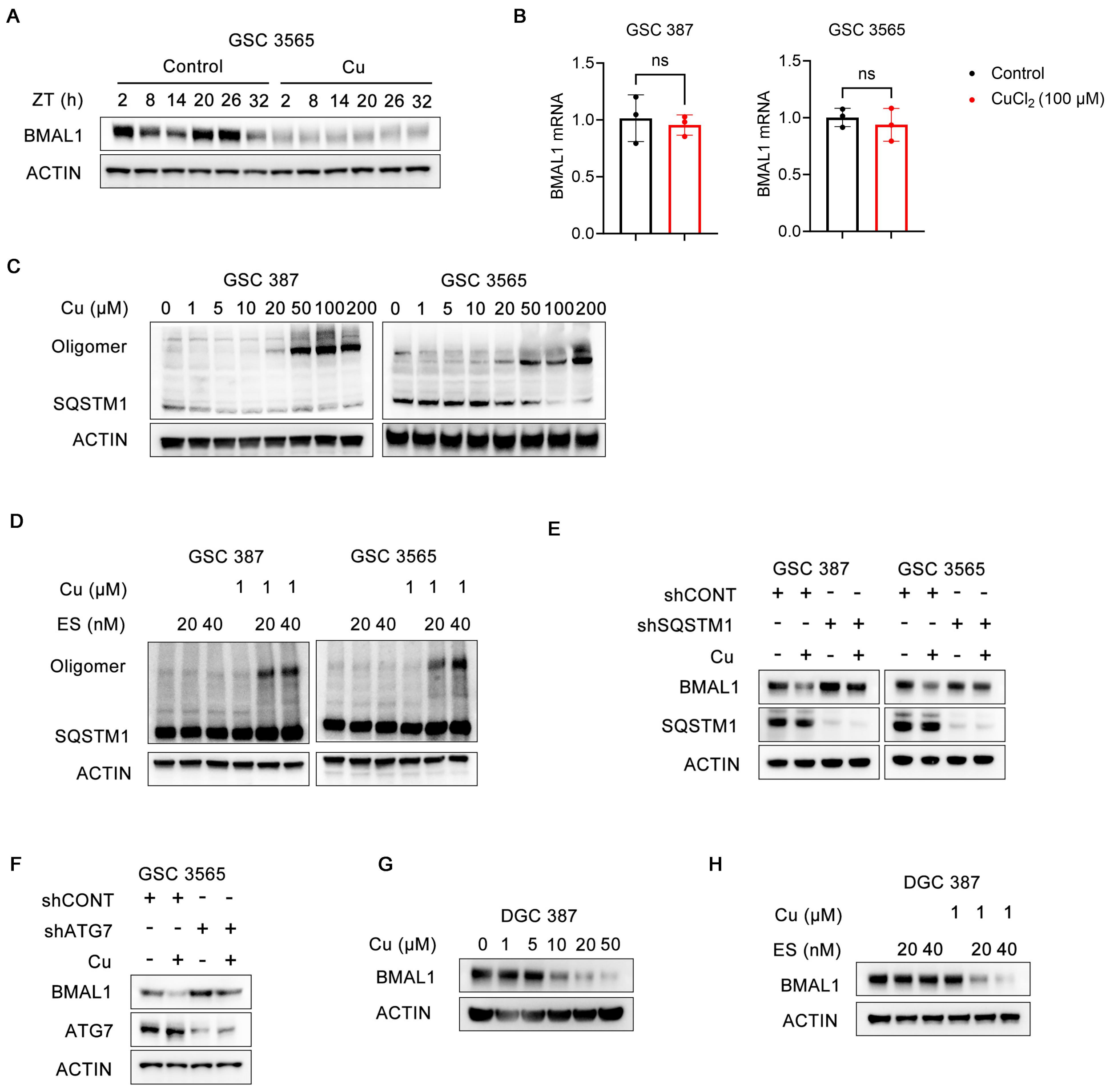
A
GSC 3565
Control
Cu
ZT (h)
2 8 14 20 26 32 2 8 14 20 26 32
BMAL1
ACTIN
B
GSC 387
GSC 3565
ns
ns
BMAL1 mRNA
1.5
1.0
0.5
0.0
Control
CuCl2 (100 μM)
C
GSC 387
GSC 3565
Cu (μM)
0 1 5 10 20 50 100 200 0 1 5 10 20 50 100 200
Oligomer
SQSTM1
ACTIN
D
GSC 387
GSC 3565
Cu (μM)
1 1 1 1 1 1
ES (nM)
20 40 20 40 20 40 20 40
Oligomer
SQSTM1
ACTIN
E
GSC 387
GSC 3565
shCONT
+ + - - + + - -
shSQSTM1
- - + + - - + +
Cu
- + - + - + - +
BMAL1
SQSTM1
ACTIN
F
GSC 3565
shCONT
+ + - -
shATG7
- - + +
Cu
- + - +
BMAL1
ATG7
ACTIN
G
DGC 387
Cu (μM)
0 1 5 10 20 50
BMAL1
ACTIN
H
DGC 387
Cu (μM)
1 1 1
ES (nM)
20 40 20 40
BMAL1
ACTIN

**Supplemental Figure 8.** (A) Western blot of BMAL1 at 6-h intervals in synchronized GSCs ± 100 μM $CuCl_2$. (B) qPCR of GSCs treated with 100 μM $CuCl_2$ for 48 h. (C–D) Western blots showing SQSTM1 and its oligomers in GSCs treated with $CuCl_2$ or elesclomol ± 1 μM $CuCl_2$. (E and F) Western blots of GSCs with SQSTM1 (shP62) or ATG7 knockdown ± 100 μM $CuCl_2$. (G and H) BMAL1 expression in DGCs treated with $CuCl_2$ or elesclomol ± 1 μM $CuCl_2$. Data are mean ± SEM. T test was conducted for B. ns not significant.

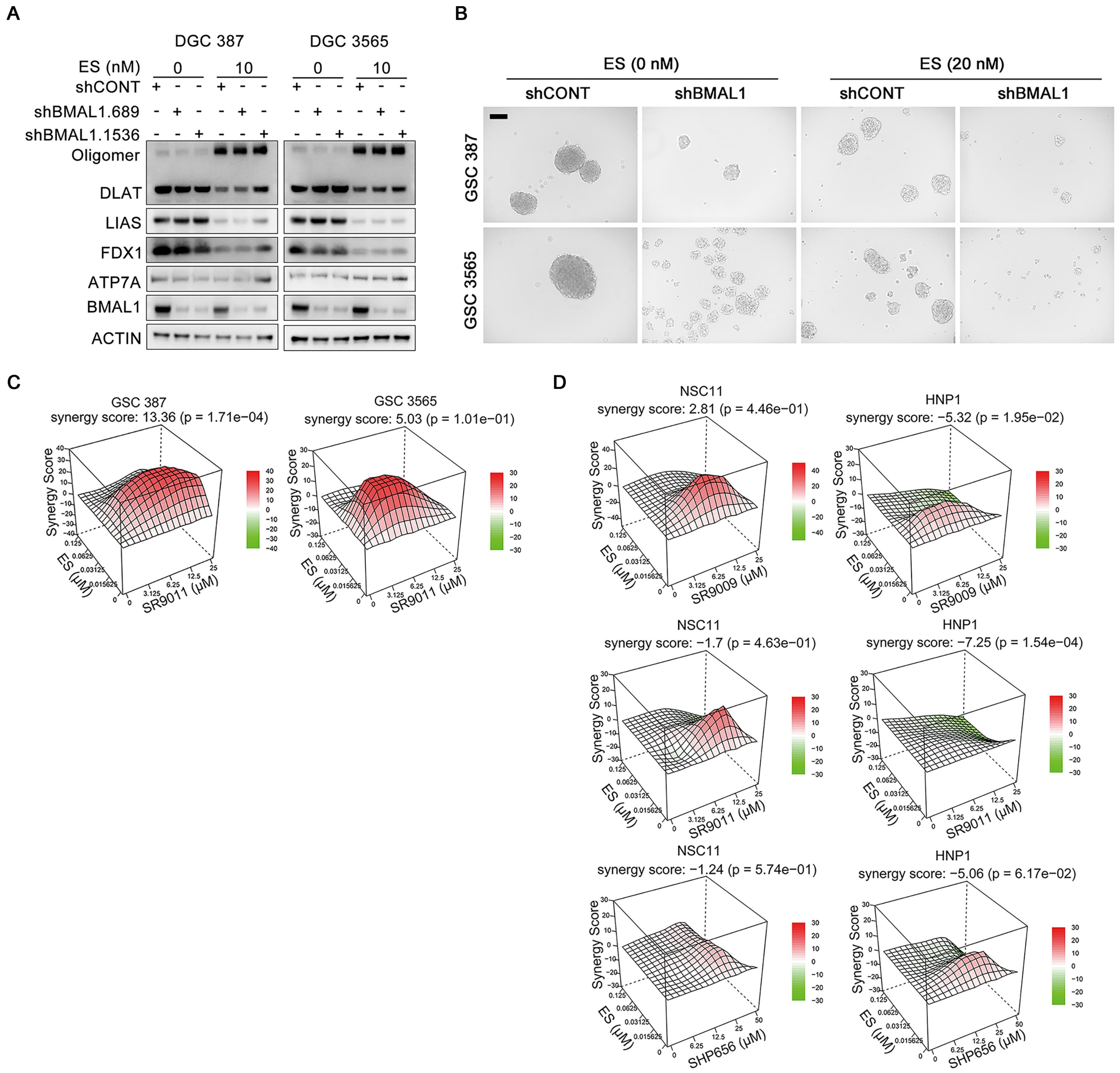
A
DGC 387
DGC 3565
ES (nM)
0
10
shCONT
shBMAL1.689
shBMAL1.1536
Oligomer
DLAT
LIAS
FDX1
ATP7A
BMAL1
ACTIN
B
ES (0 nM)
ES (20 nM)
shCONT
shBMAL1
GSC 387
GSC 3565
C
GSC 387
synergy score: 13.36 (p = 1.71e−04)
GSC 3565
synergy score: 5.03 (p = 1.01e−01)
Synergy Score
ES (μM)
SR9011 (μM)
D
NSC11
synergy score: 2.81 (p = 4.46e−01)
HNP1
synergy score: −5.32 (p = 1.95e−02)
SR9009 (μM)
NSC11
synergy score: −1.7 (p = 4.63e−01)
HNP1
synergy score: −7.25 (p = 1.54e−04)
SR9011 (μM)
NSC11
synergy score: −1.24 (p = 5.74e−01)
HNP1
synergy score: −5.06 (p = 6.17e−02)
SHP656 (μM)

**Supplemental Figure 9.**

(A) Immunoblots showing ES (20 nM, 48 h)–induced cuproptosis in DGCs with control or BMAL1 knockdown (shBMAL1.689, shBMAL1.1536; 1 μM $CuCl_2$). (B) Representative sphere formation images from Figure 7D. Scale bar: 100 μm. (C and D) SynergyFinder plots showing combined effects of ES with SR9011 in GSCs (C) and with SHP656, SR9009, or SR9011 in NSCs (D).

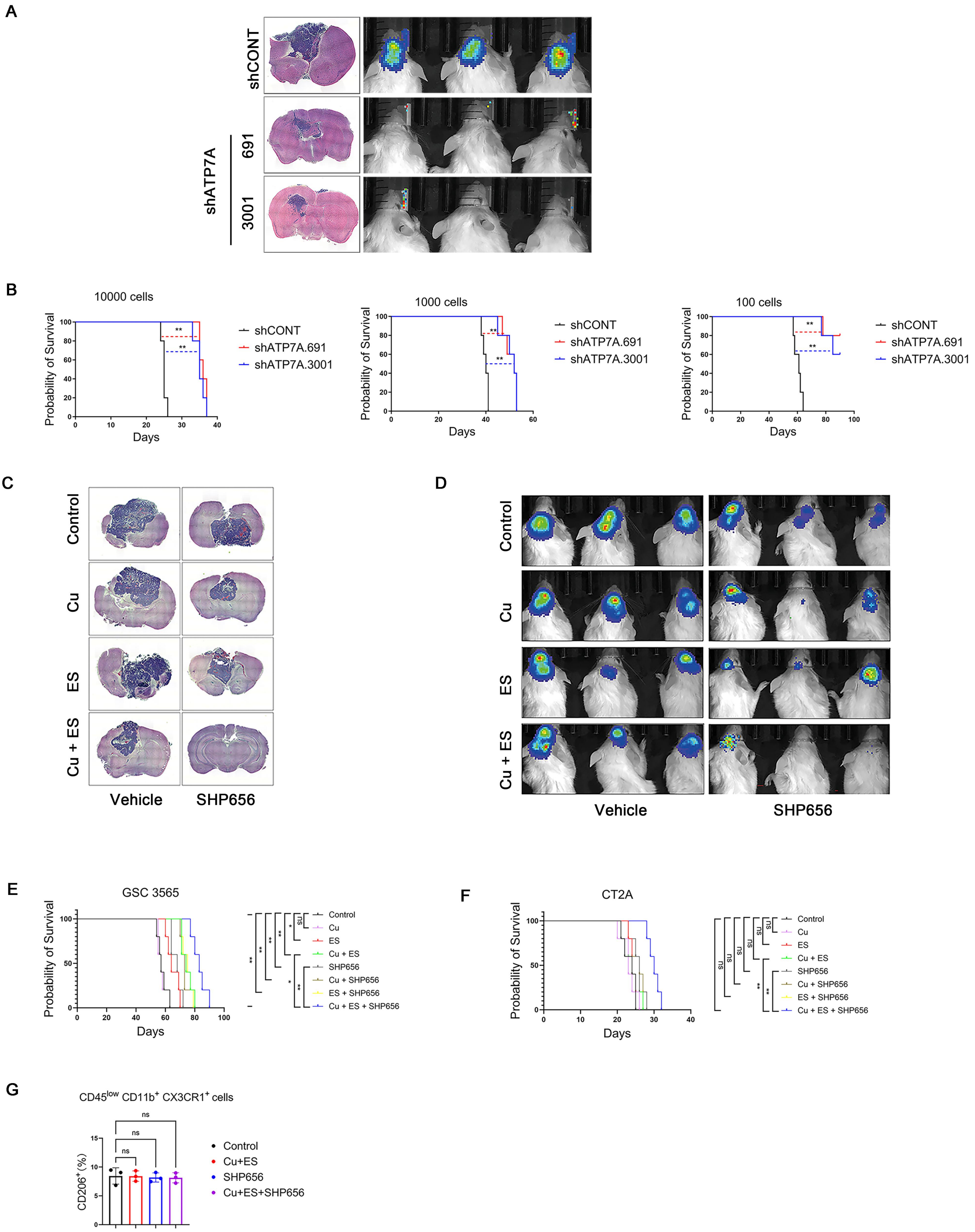
A
shCONT
shATP7A
691
3001
B
10000 cells
1000 cells
100 cells
Probability of Survival
Days
shCONT
shATP7A.691
shATP7A.3001
C
Control
Cu
ES
Cu + ES
Vehicle
SHP656
D
Control
Cu
ES
Cu + ES
Vehicle
SHP656
E
GSC 3565
Probability of Survival
Days
Control
Cu
ES
Cu + ES
SHP656
Cu + SHP656
ES + SHP656
Cu + ES + SHP656
F
CT2A
Probability of Survival
Days
Control
Cu
ES
Cu + ES
SHP656
Cu + SHP656
ES + SHP656
Cu + ES + SHP656
G
CD45low CD11b+ CX3CR1+ cells
CD206+ (%)
ns
Control
Cu+ES
SHP656
Cu+ES+SHP656

**Supplemental Figure 10.**

(A) Bioluminescence imaging and total flux quantification (n=5) of mice bearing GSC3565 tumors ± ATP7A knockdown. Representative H&E-stained brain sections shown; scale bar: 1 mm. (B) Kaplan–Meier survival of mice implanted with $10^4$, $10^3$, or $10^2$ GSC387 cells expressing control or ATP7A shRNAs (n=5/group). (C and D) Intracranial GSC387 tumors treated with copper gluconate, elesclomol, SHP656, or combination; H&E staining (C) and bioluminescence imaging (D). (E) Kaplan–Meier survival of GSC3565 tumor–bearing mice receiving indicated treatments (n=5/group). (F) Kaplan–Meier survival of CT2A-bearing immunocompetent mice treated as above (n=5/group). (G) Flow cytometry of $CD206^+$ cells among CD45^low$CD11b^+CX3CR^+$ populations. Log-rank test was performed for B, E, and F. One-way ANOVA for G. **p<0.01; *p<0.05; ns, not significant.

## SUPPLEMENTAL TABLES

Supplemental Table 1. sgRNA in crispr library

Supplemental Table 2. sgRNA counts

Supplemental Table 3. MAGeCK test

Supplemental Table 4. Lipdomic data

Supplemental Table 5. Peptides in MS

Supplemental Table 6. MS for BMAL1-binding proteins

Supplemental Table 7. GOBP:SELECTIVE_AUTOPHAGY, GO0061912

Supplemental Table 8. Primers

Supplemental Table 9. Reagents and antibodies
