# Supplementary material for: Glioblastoma stem cells resist cuproptosis with circadian variation of copper levels
Source: J Clin Invest. 2026 Jan 2;136(1):e192599. doi: 10.1172/JCI192599 (PMC12721906; doi:10.1172/JCI192599)
Supplement: Unedited blot and gel images [file jci-136-192599-s010.pdf]

Figure 1D

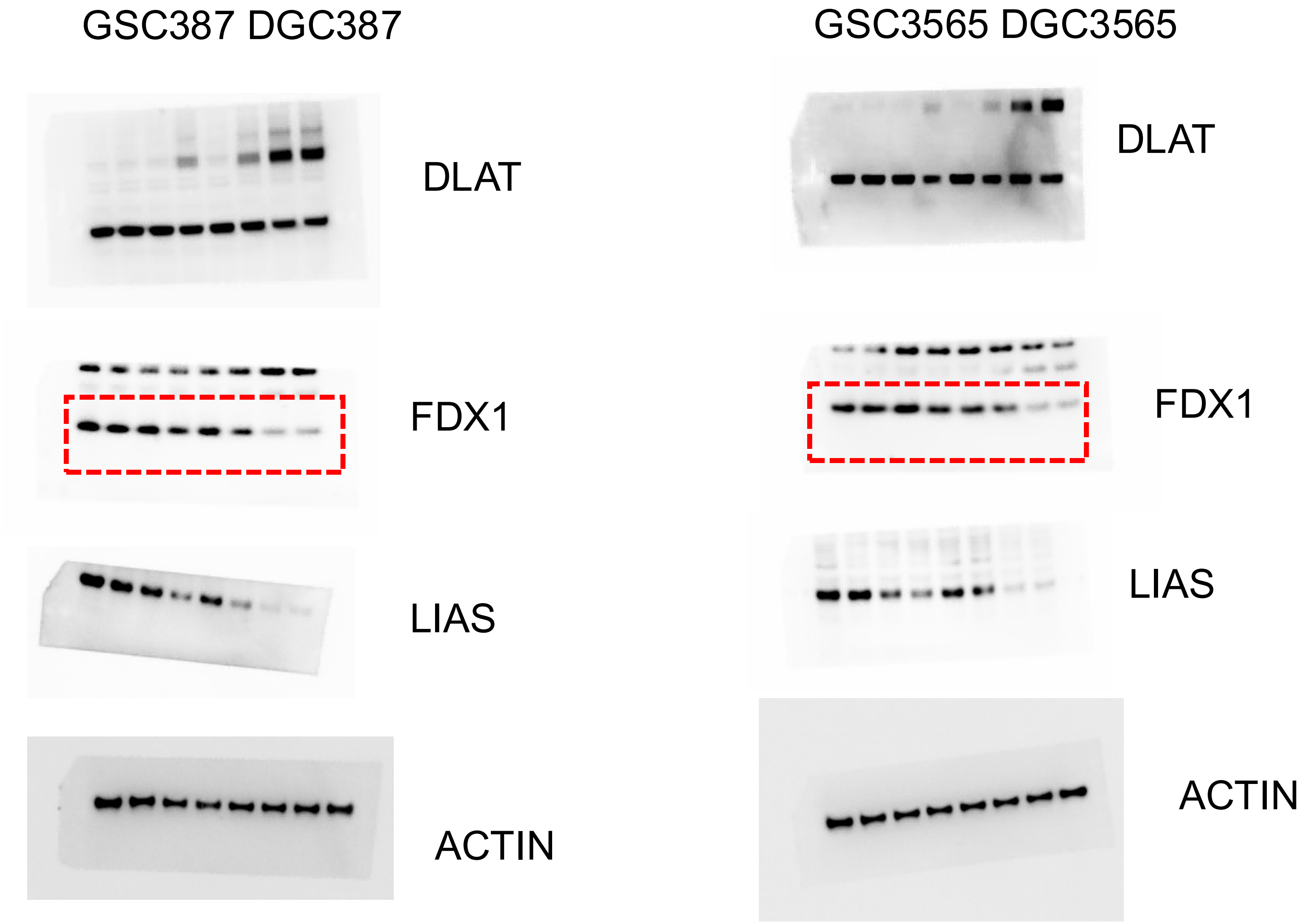
GSC387 DGC387
DLAT
FDX1
LIAS
ACTIN
GSC3565 DGC3565
DLAT
FDX1
LIAS
ACTIN

Figure 3F

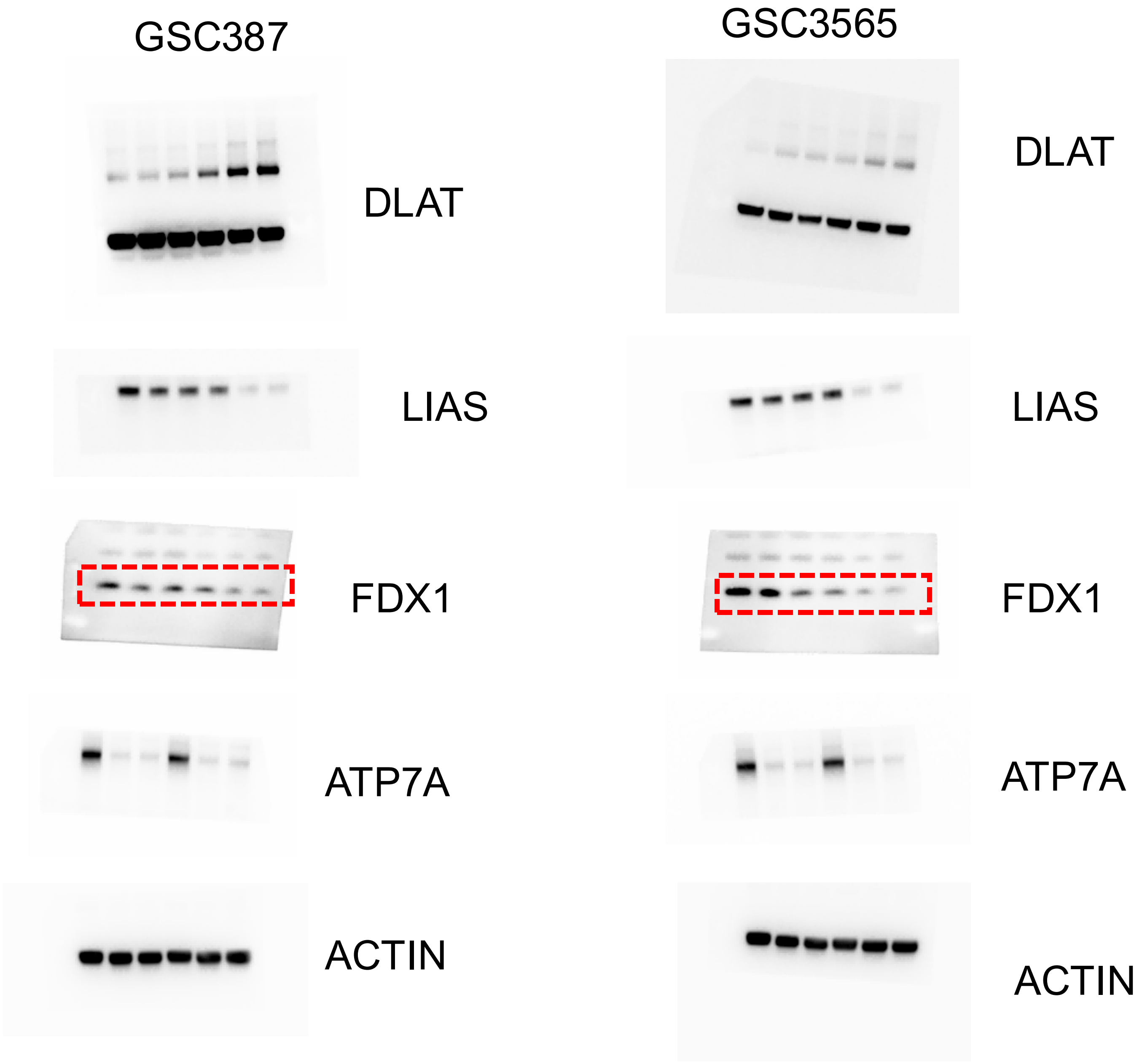

Figure 3G

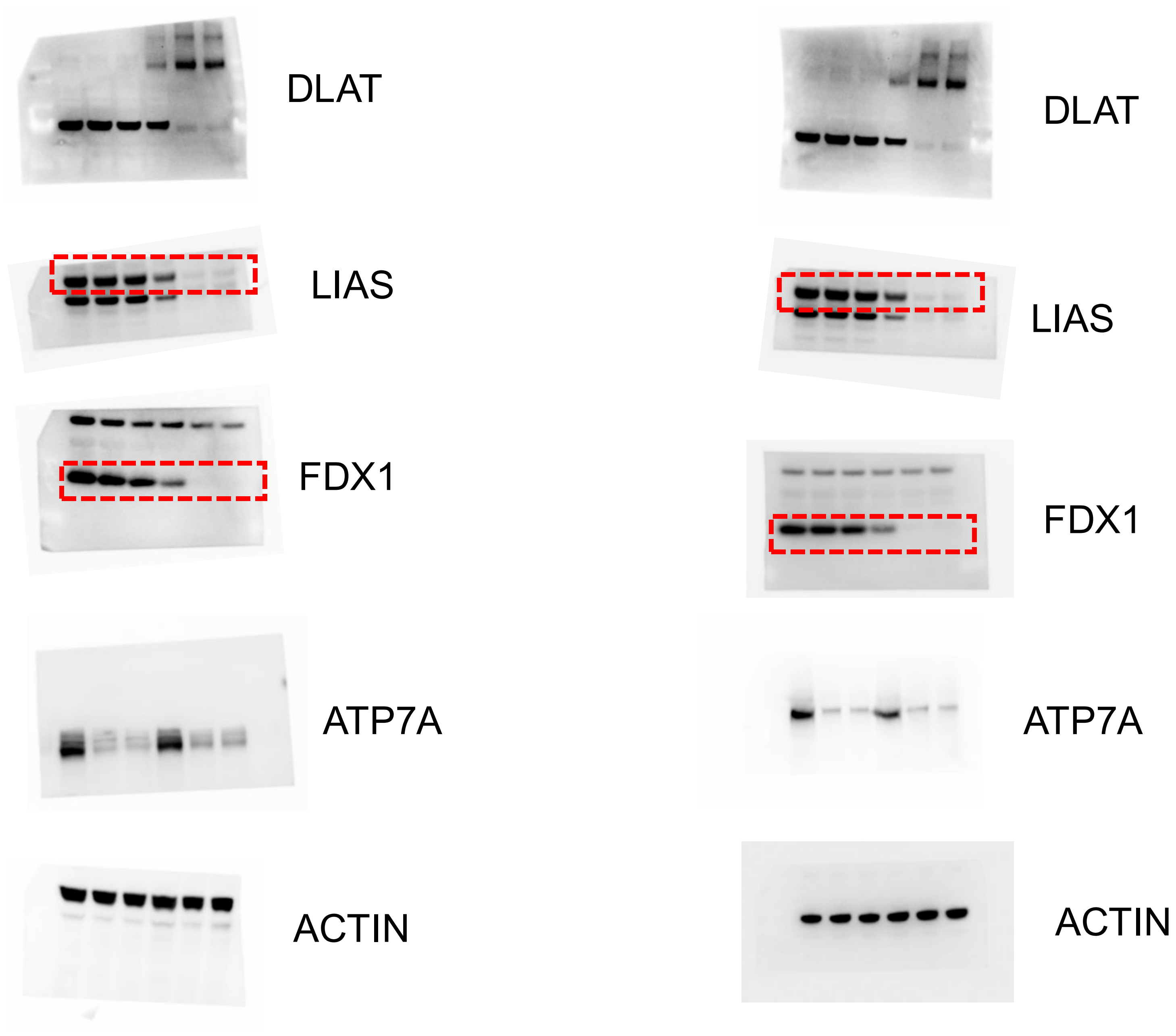

Figure 4D

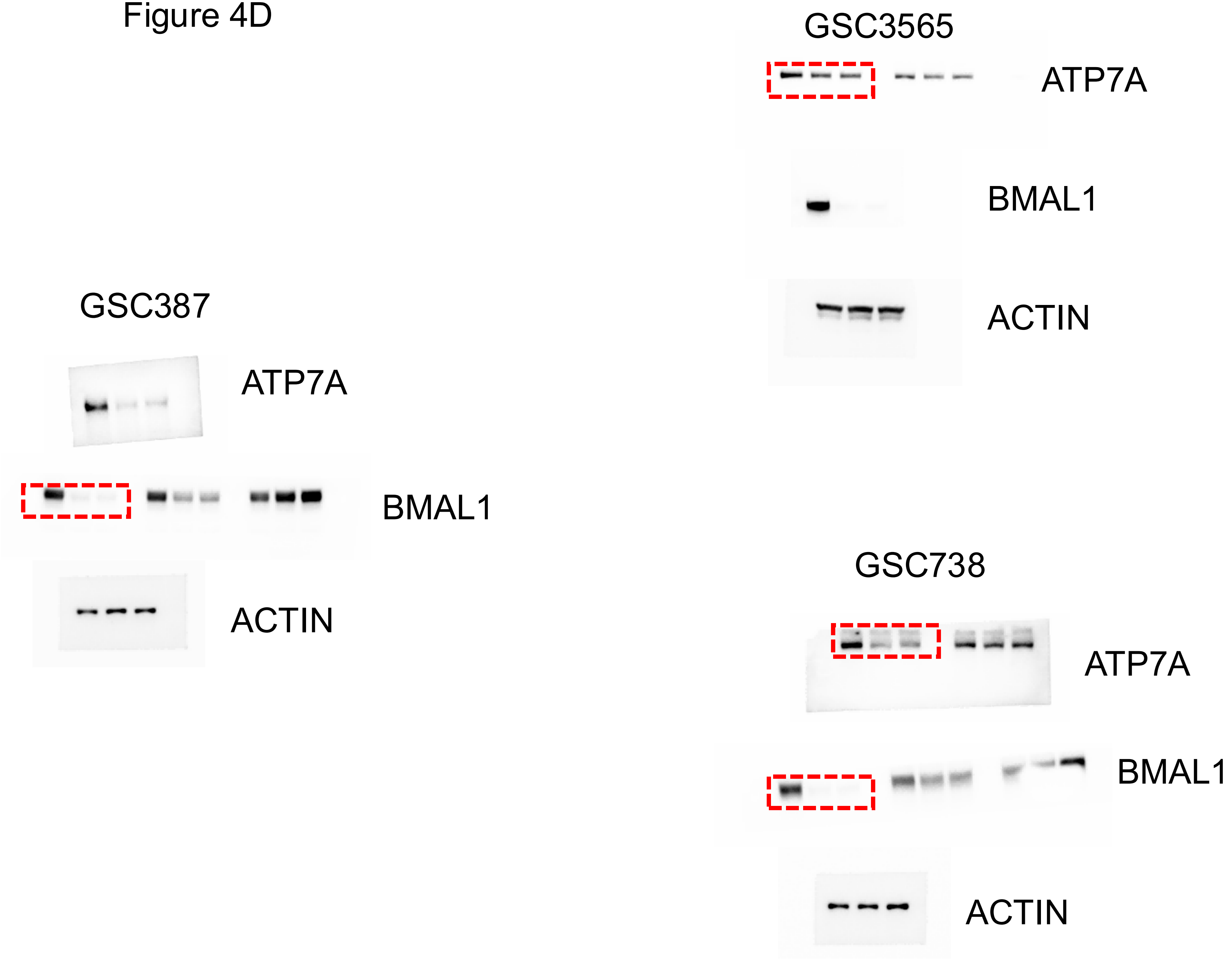
GSC3565
ATP7A
BMAL1
ACTIN
GSC387
ATP7A
BMAL1
ACTIN
GSC738
ATP7A
BMAL1
ACTIN

Figure 4E

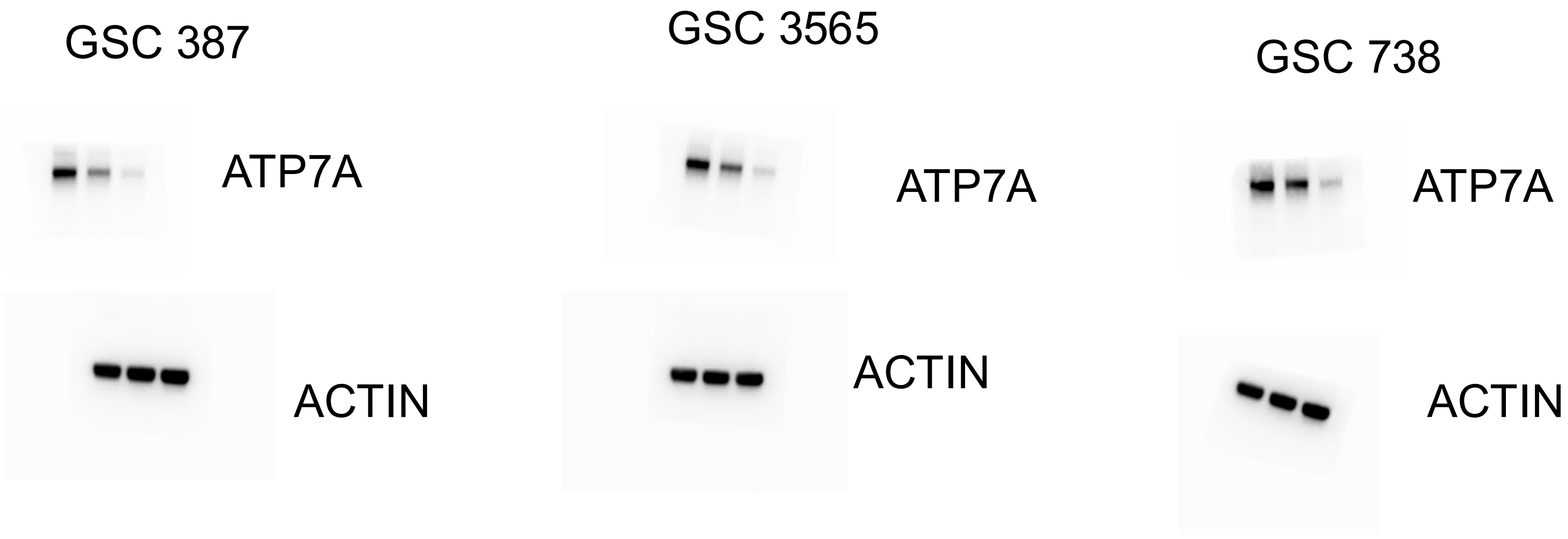

Figure 4F

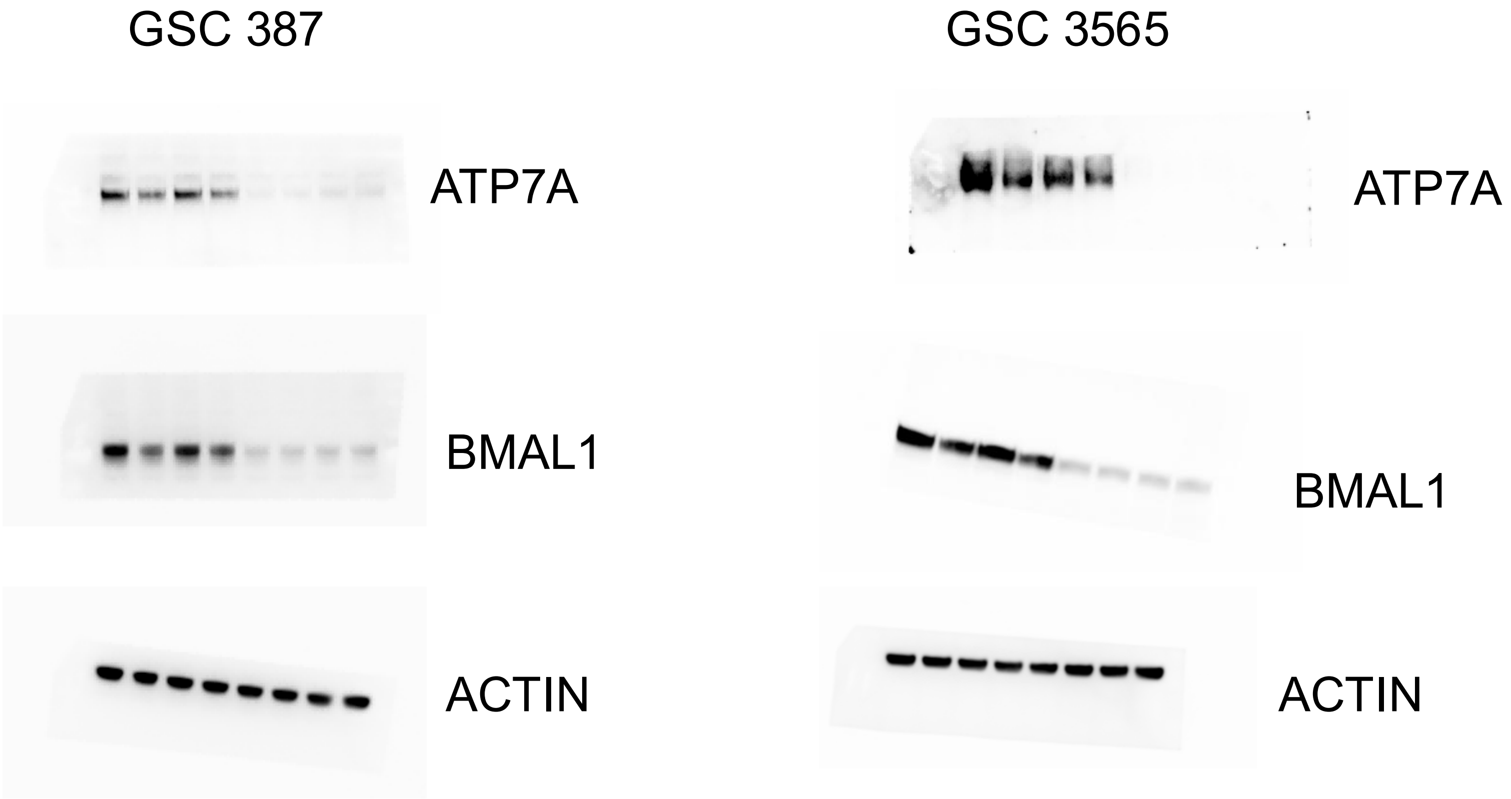

Figure 5E

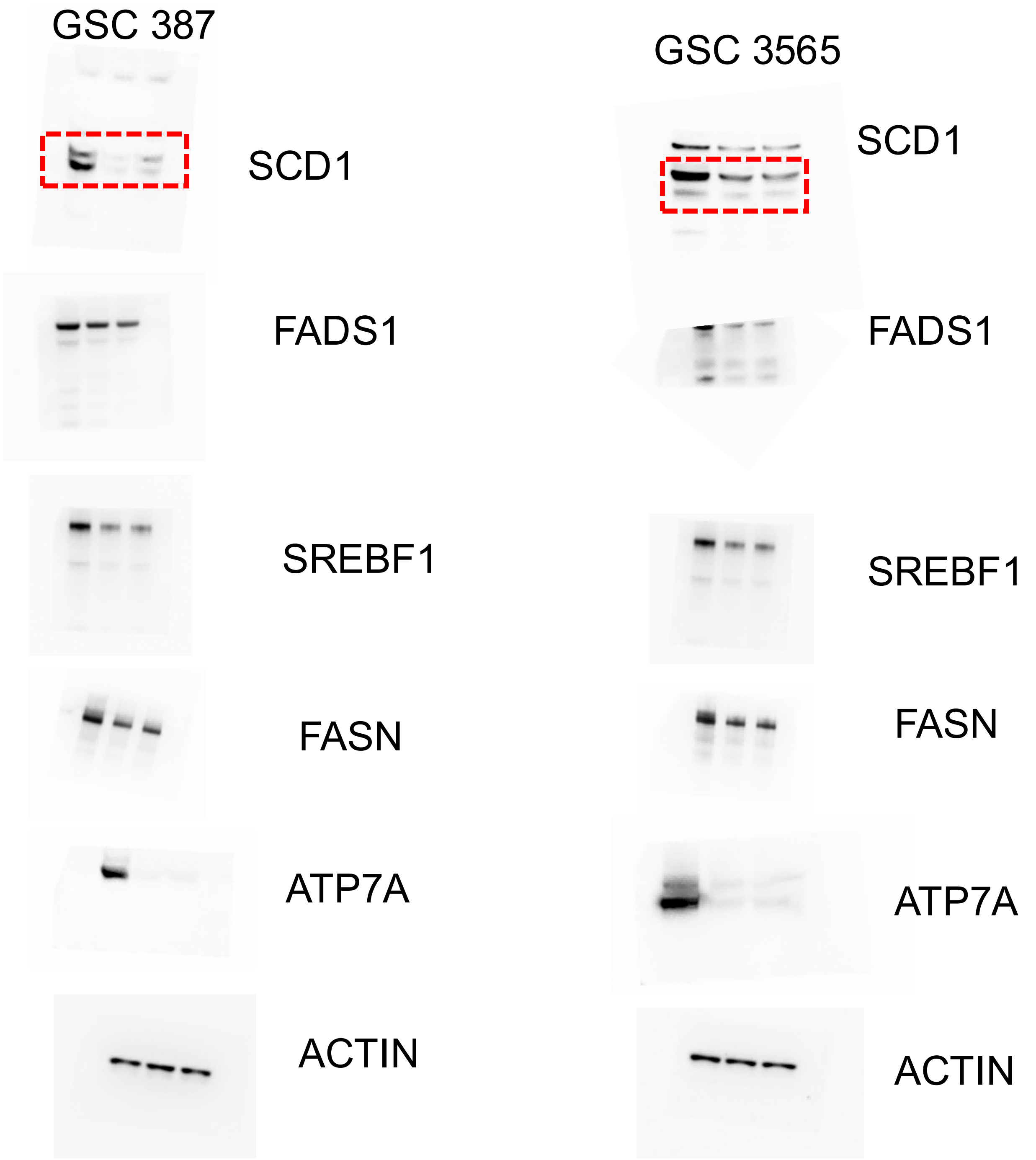

GSC 387
GSC 3565
SCD1
FADS1
SREBF1
FASN
ATP7A
ACTIN
SCD1
FADS1
SREBF1
FASN
ATP7A
ACTIN

Figure 6A

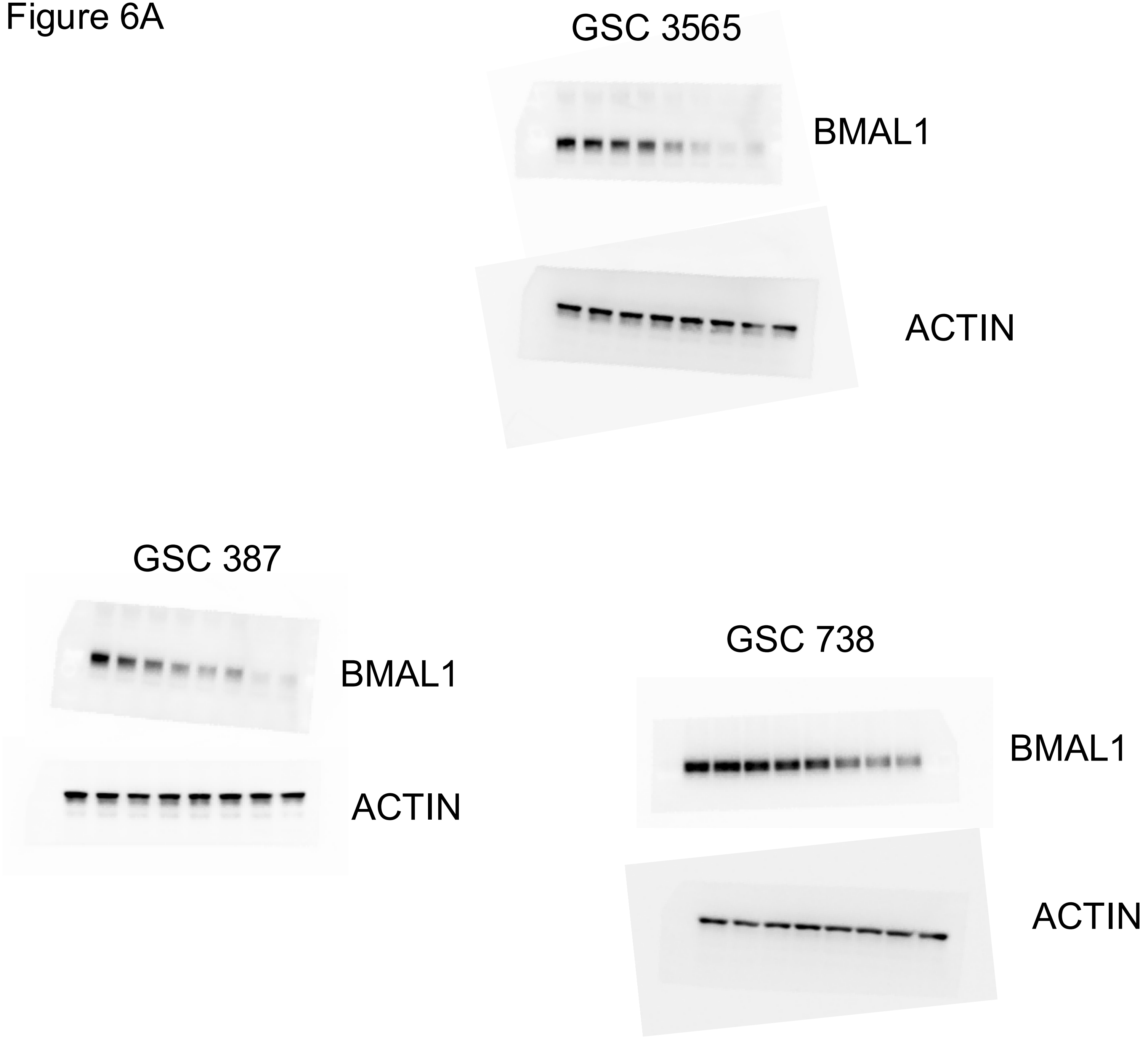

Figure 6B

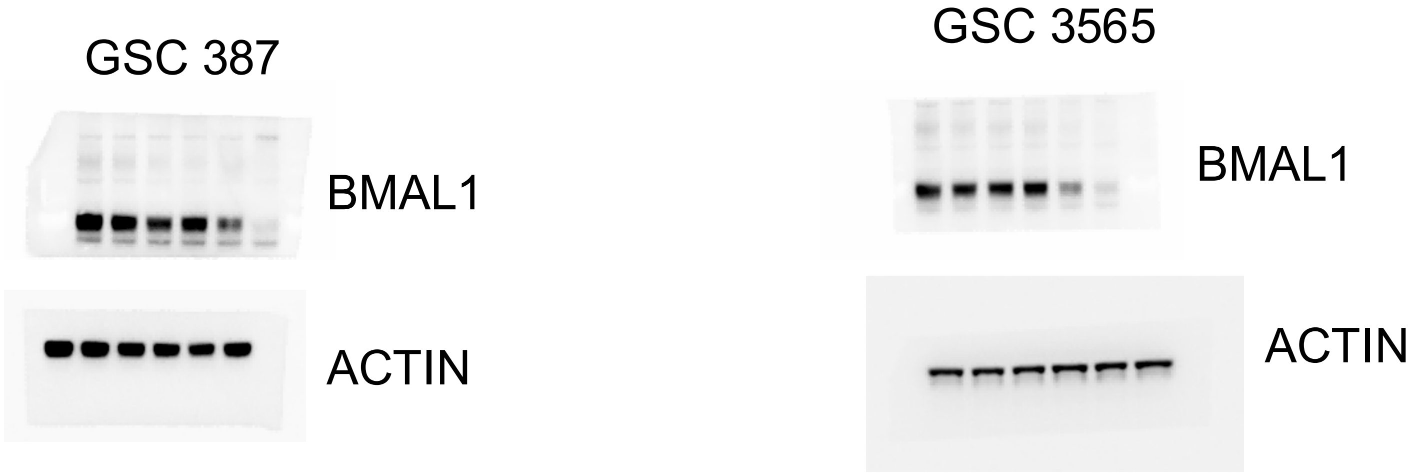

Figure 6C

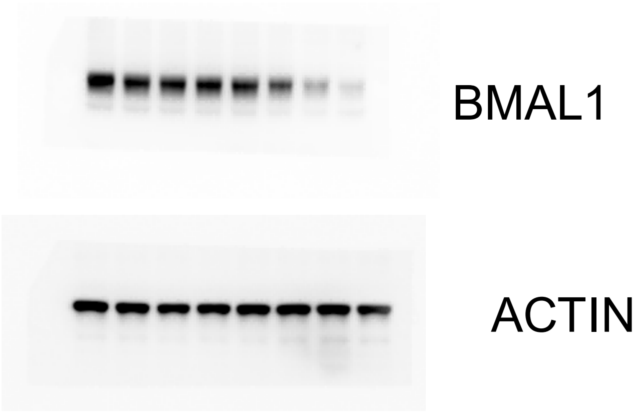

Figure 6D

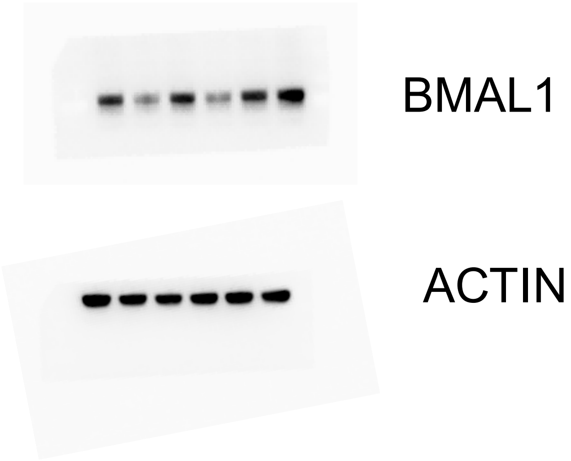

Figure 6F left part

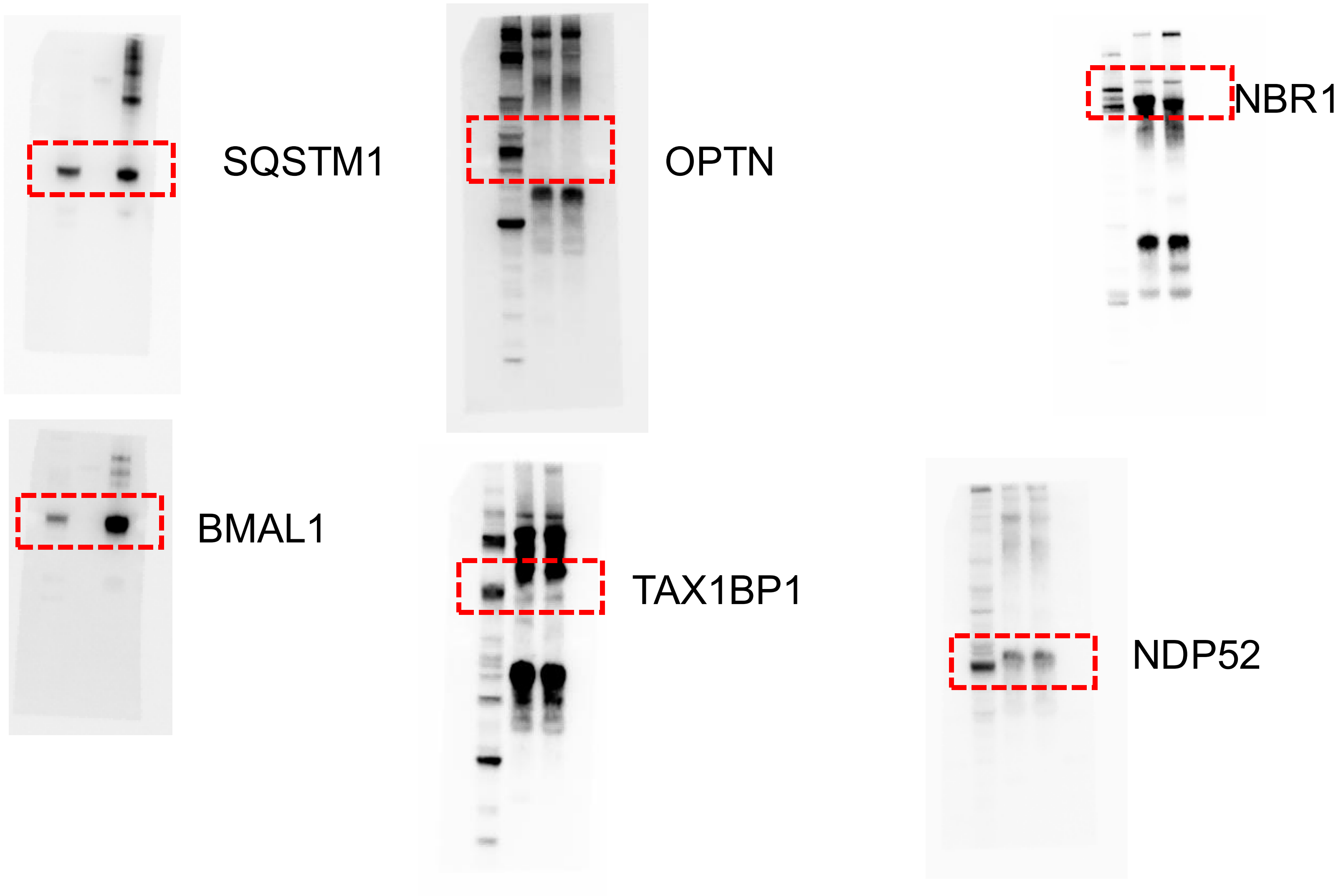

Figure 6F right part

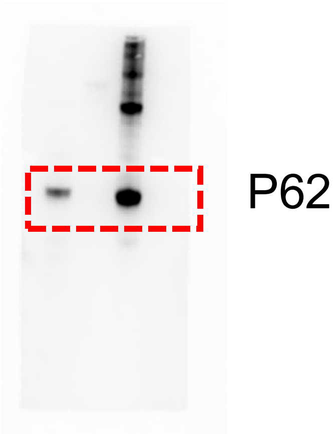

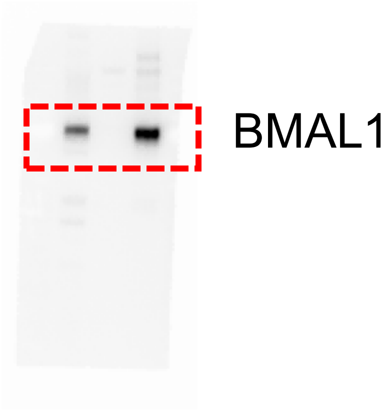

Figure 6G

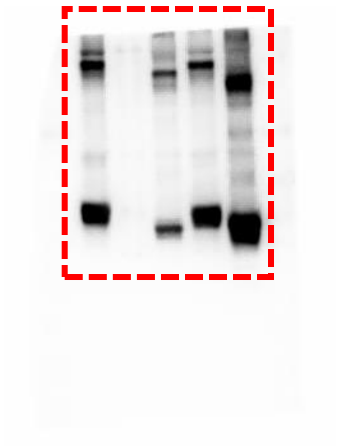

IP:HA
IB: Flag

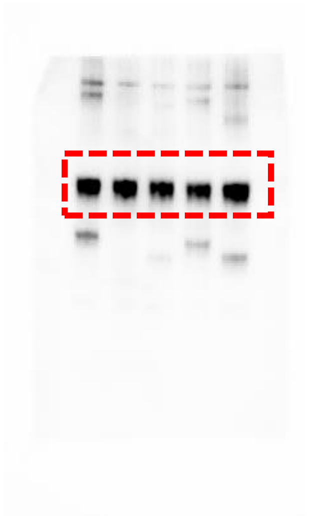

IP:HA
IB: HA

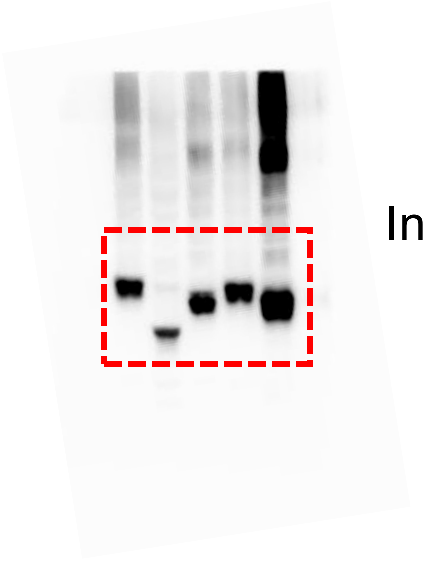

Input Flag

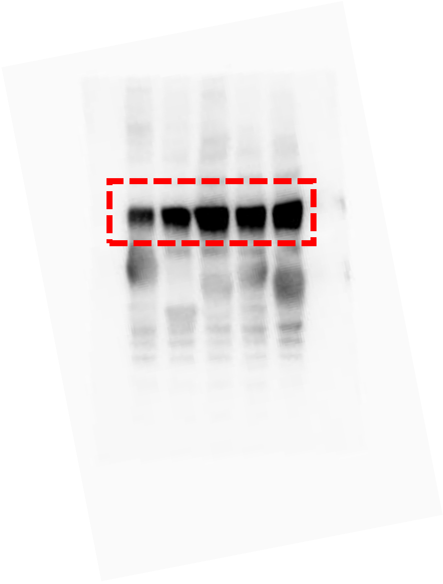

Input HA

Figure 6H

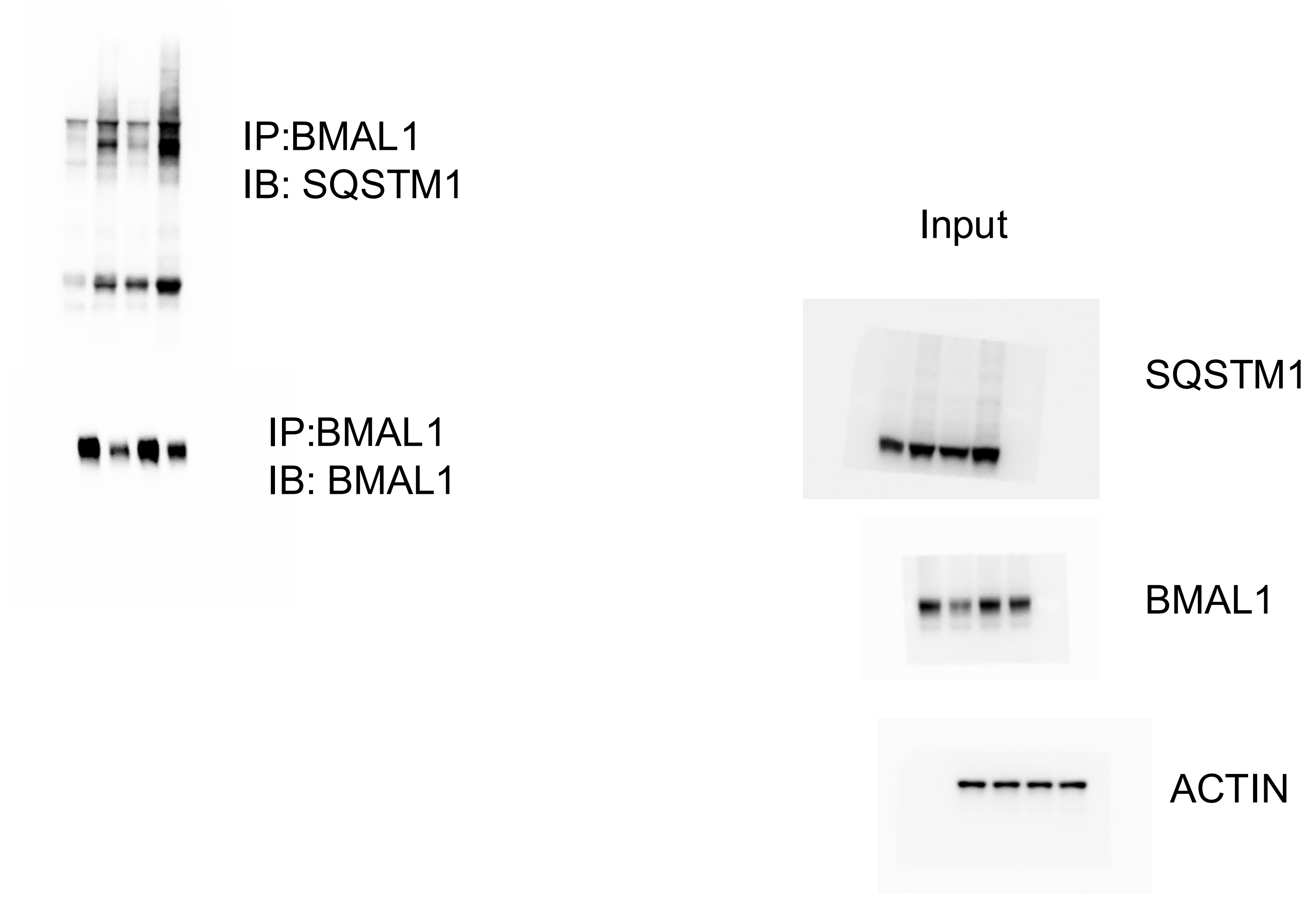
IP:BMAL1
IB: SQSTM1
IP:BMAL1
IB: BMAL1
Input
SQSTM1
BMAL1
ACTIN

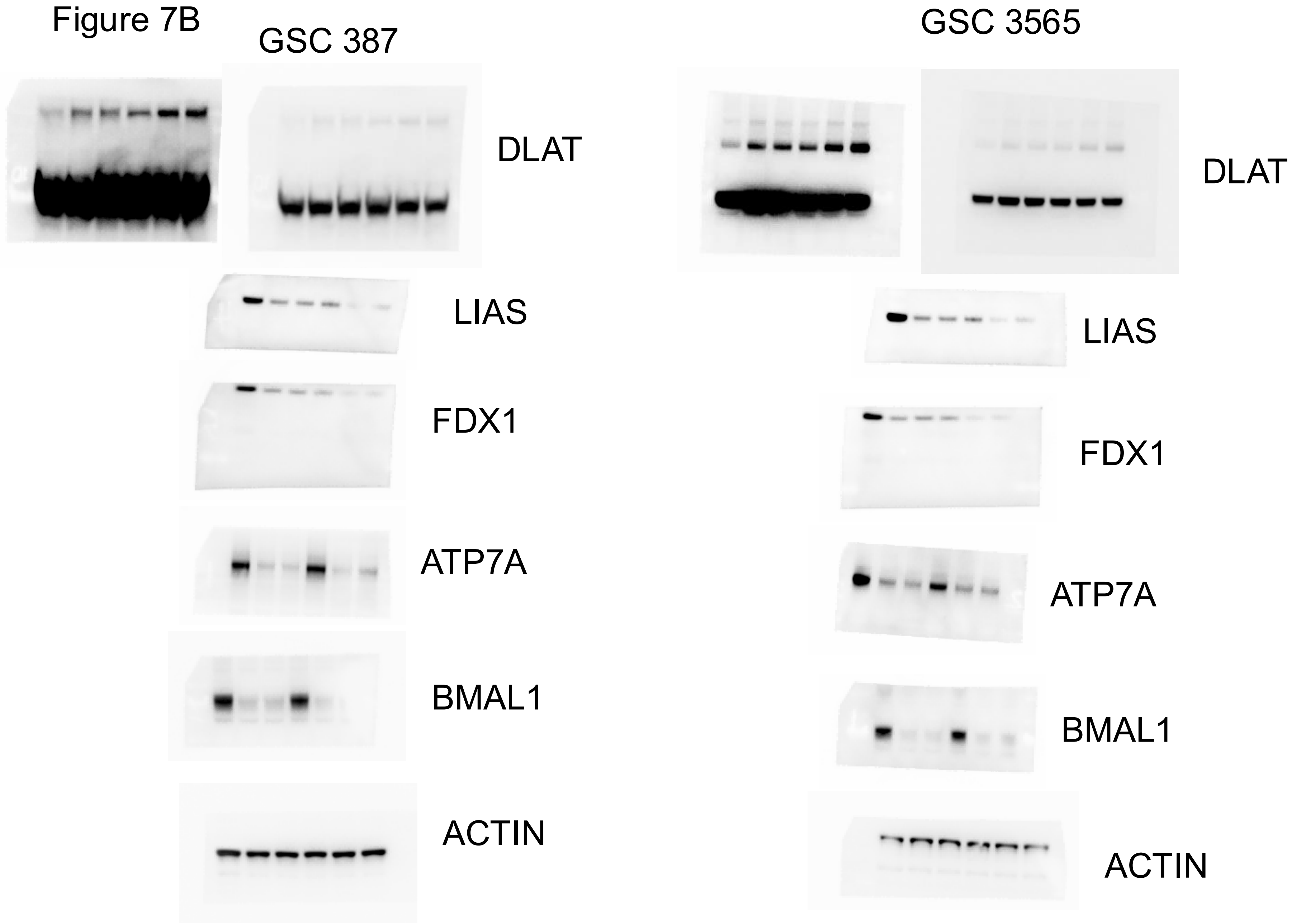
Figure 7B
GSC 387
GSC 3565
DLAT
LIAS
FDX1
ATP7A
BMAL1
ACTIN
DLAT
LIAS
FDX1
ATP7A
BMAL1
ACTIN

Figure 7G

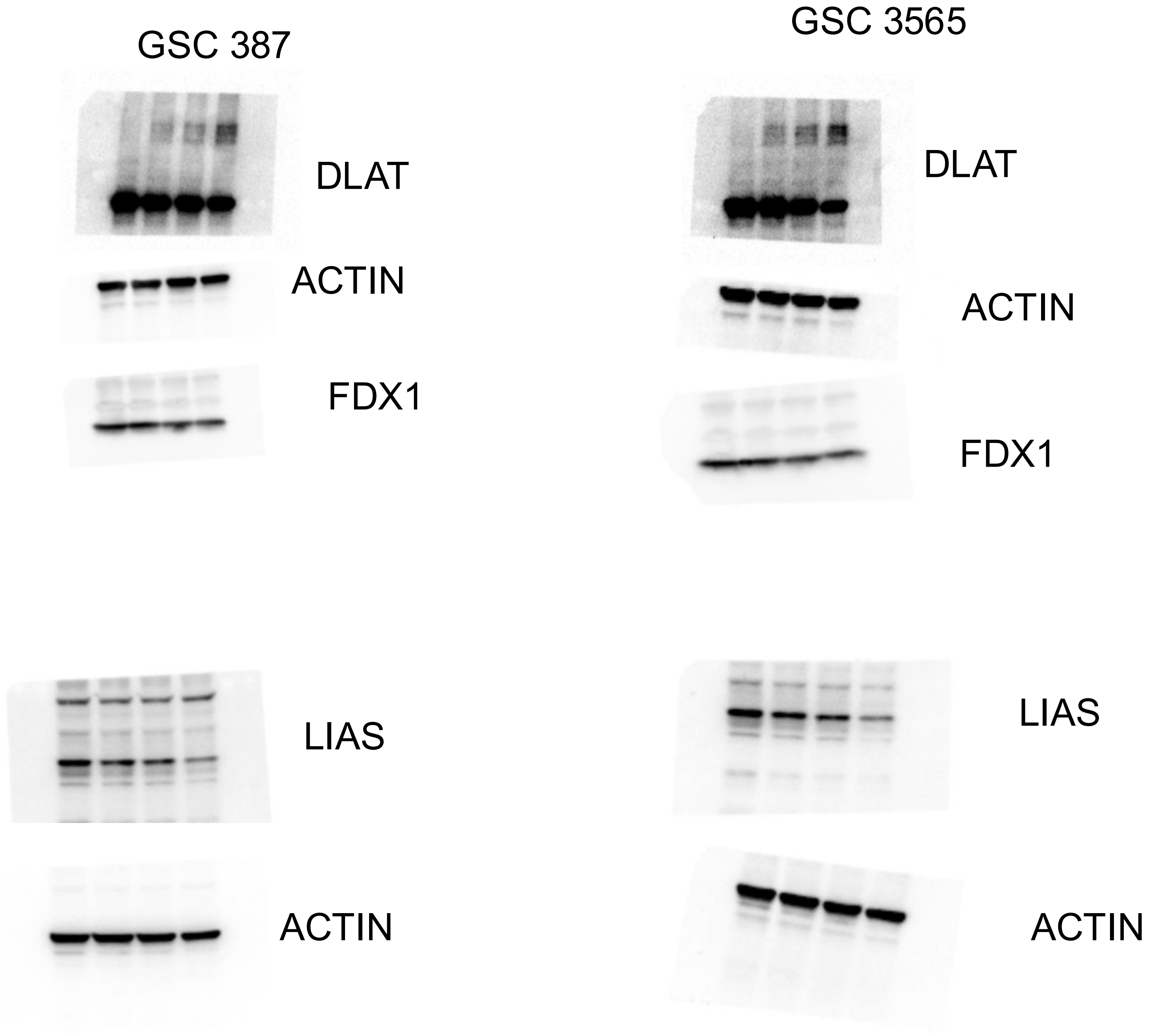
GSC 387
GSC 3565
DLAT
ACTIN
FDX1
DLAT
ACTIN
FDX1
LIAS
ACTIN
LIAS
ACTIN

Figure 7H

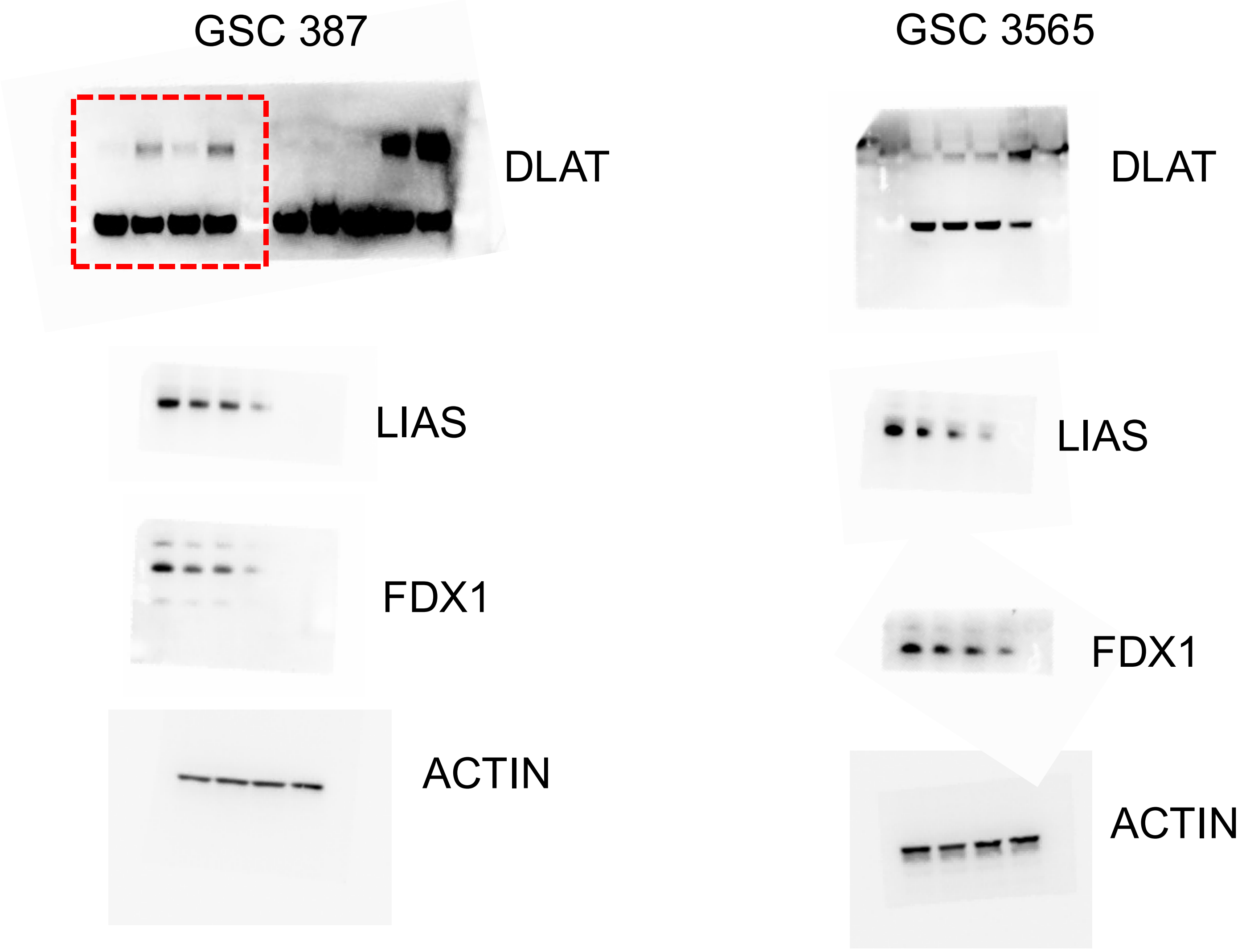

Figure S1B

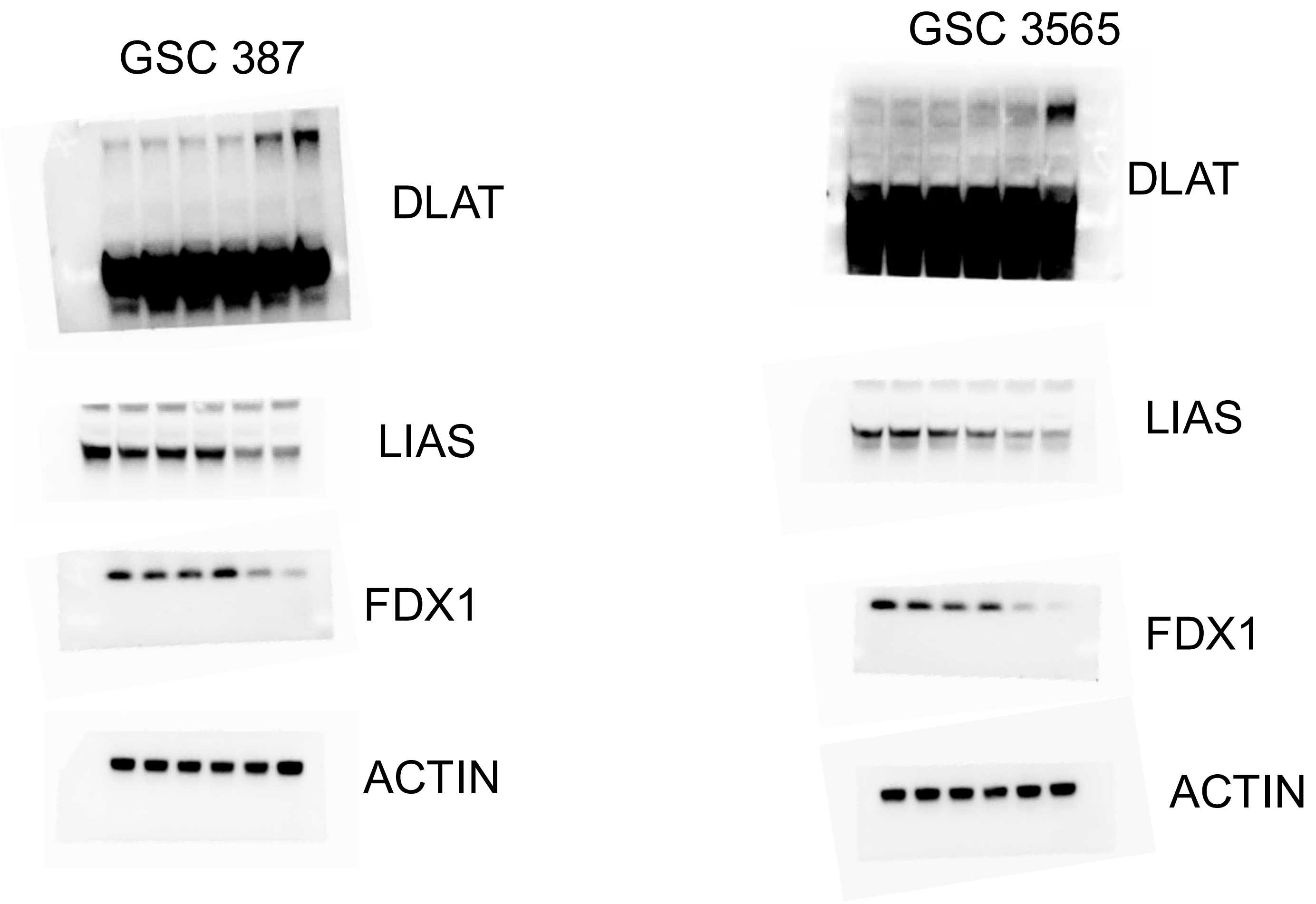

Figure S4E

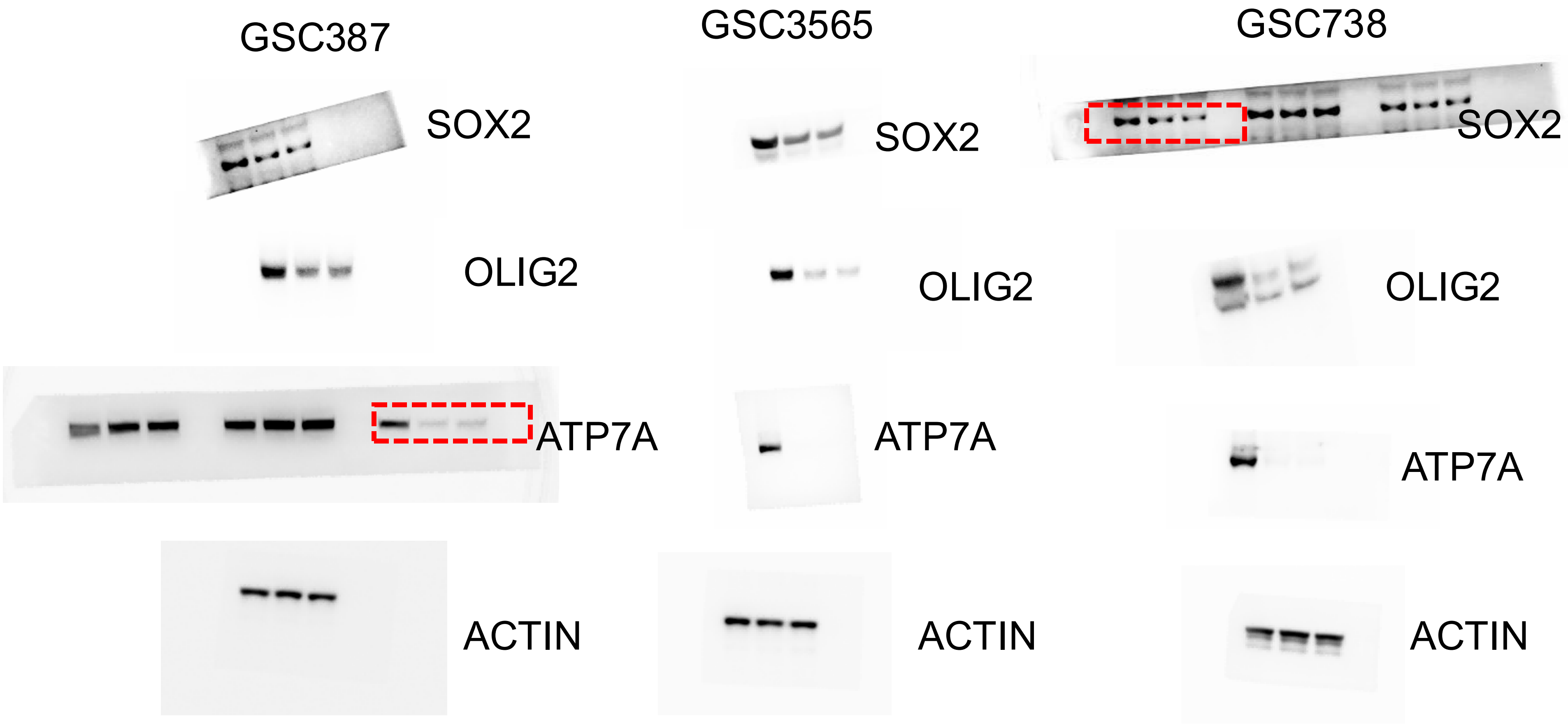
GSC387
GSC3565
GSC738
SOX2
SOX2
SOX2
OLIG2
OLIG2
OLIG2
ATP7A
ATP7A
ATP7A
ACTIN
ACTIN
ACTIN

Figure S5C

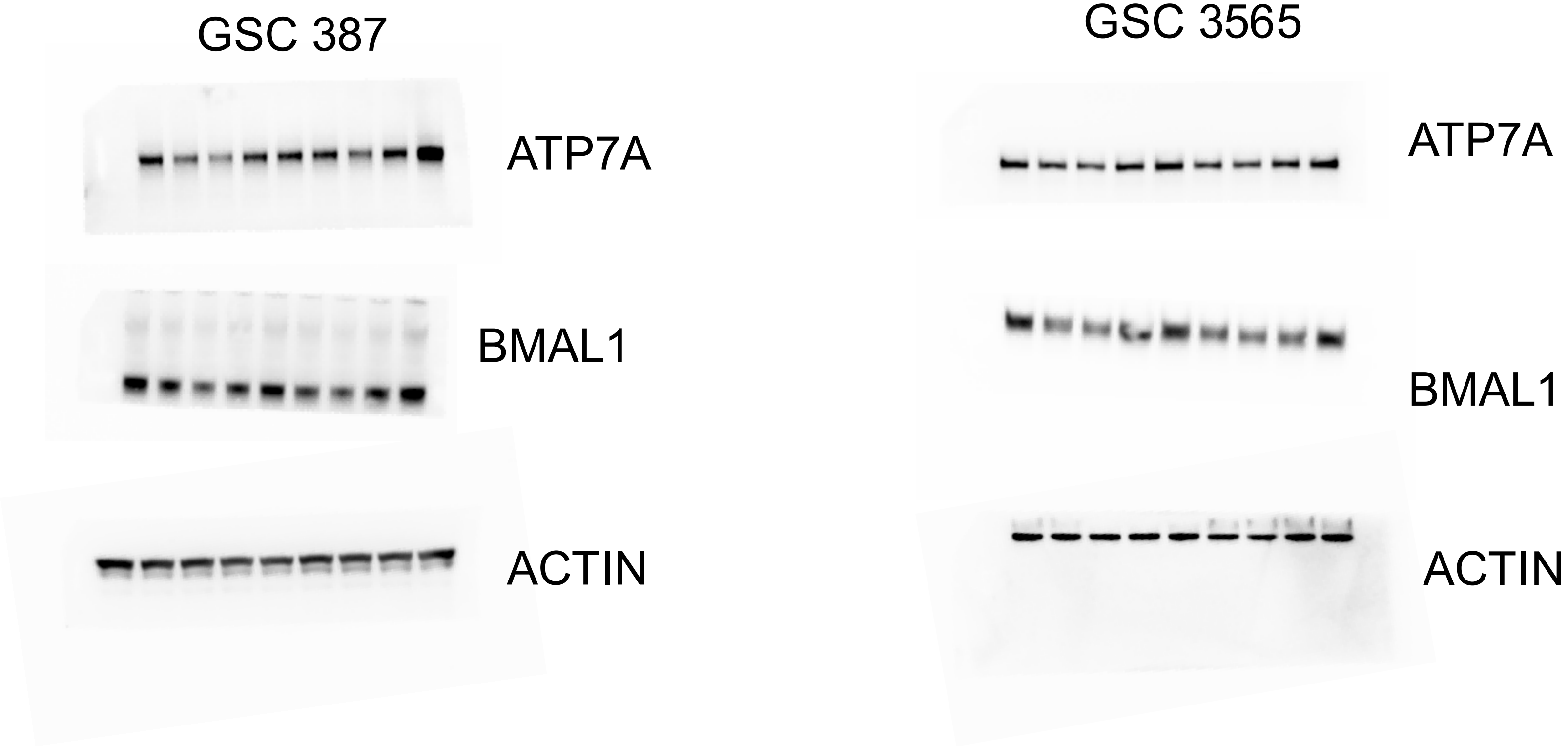

Figure S5C

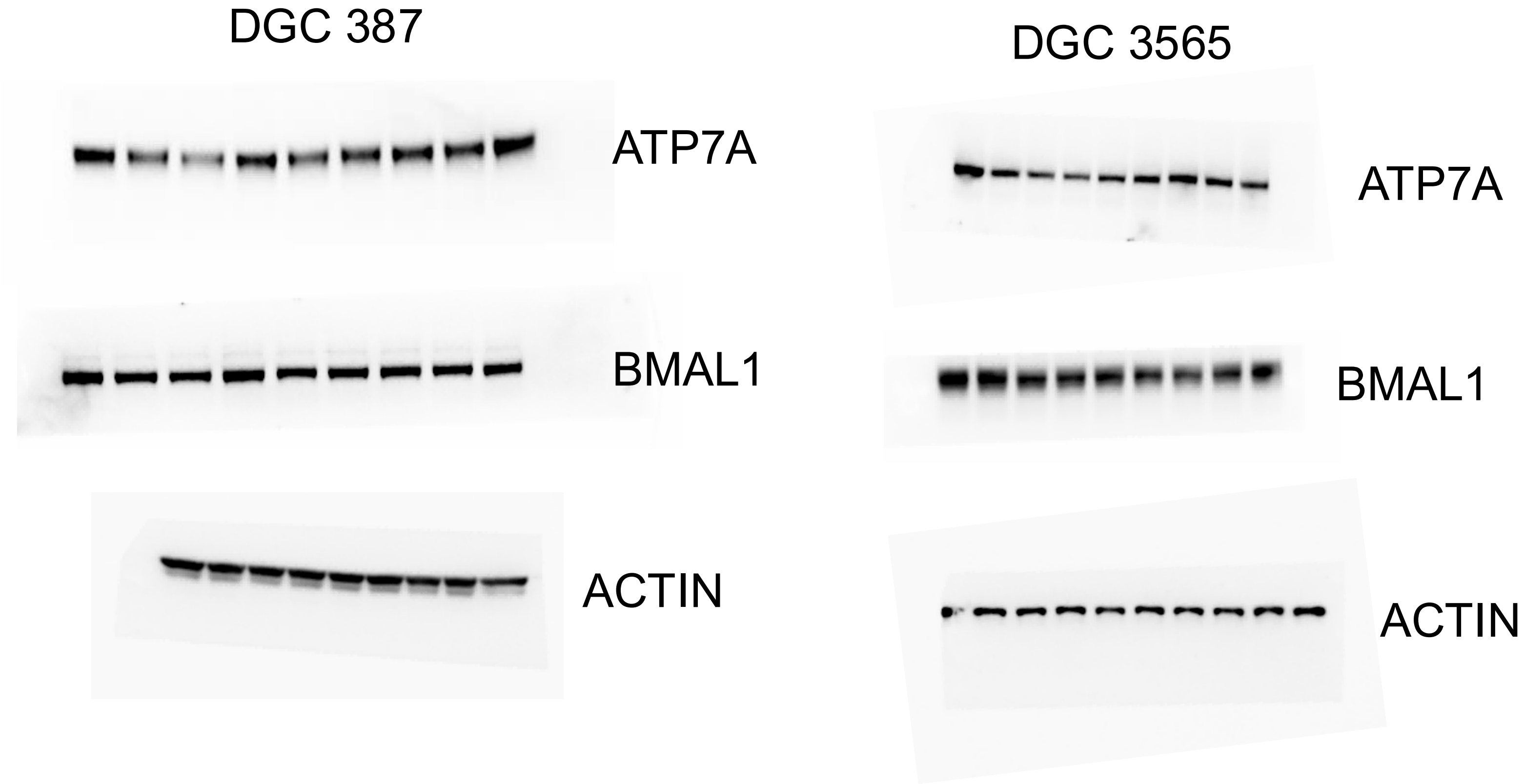

Figure S6A

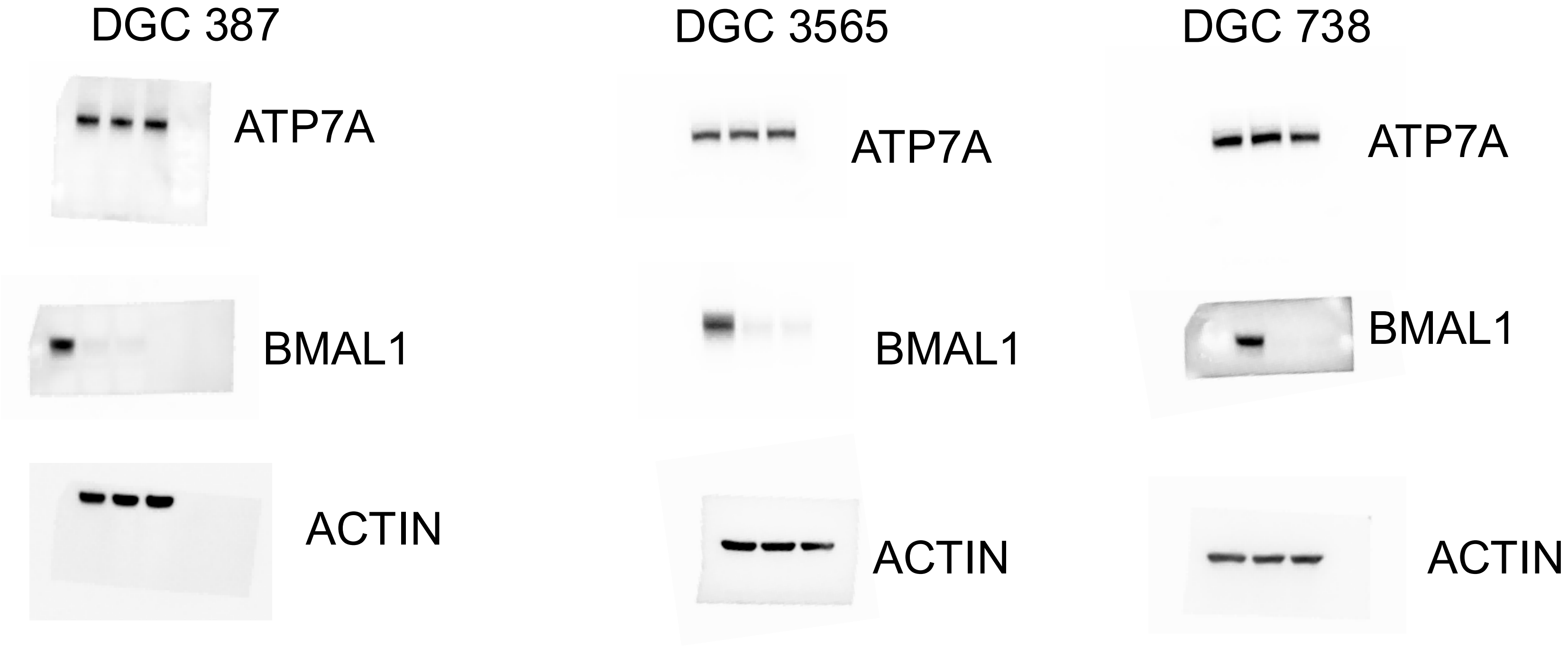
DGC 387
ATP7A
BMAL1
ACTIN
DGC 3565
ATP7A
BMAL1
ACTIN
DGC 738
ATP7A
BMAL1
ACTIN

Figure S6C

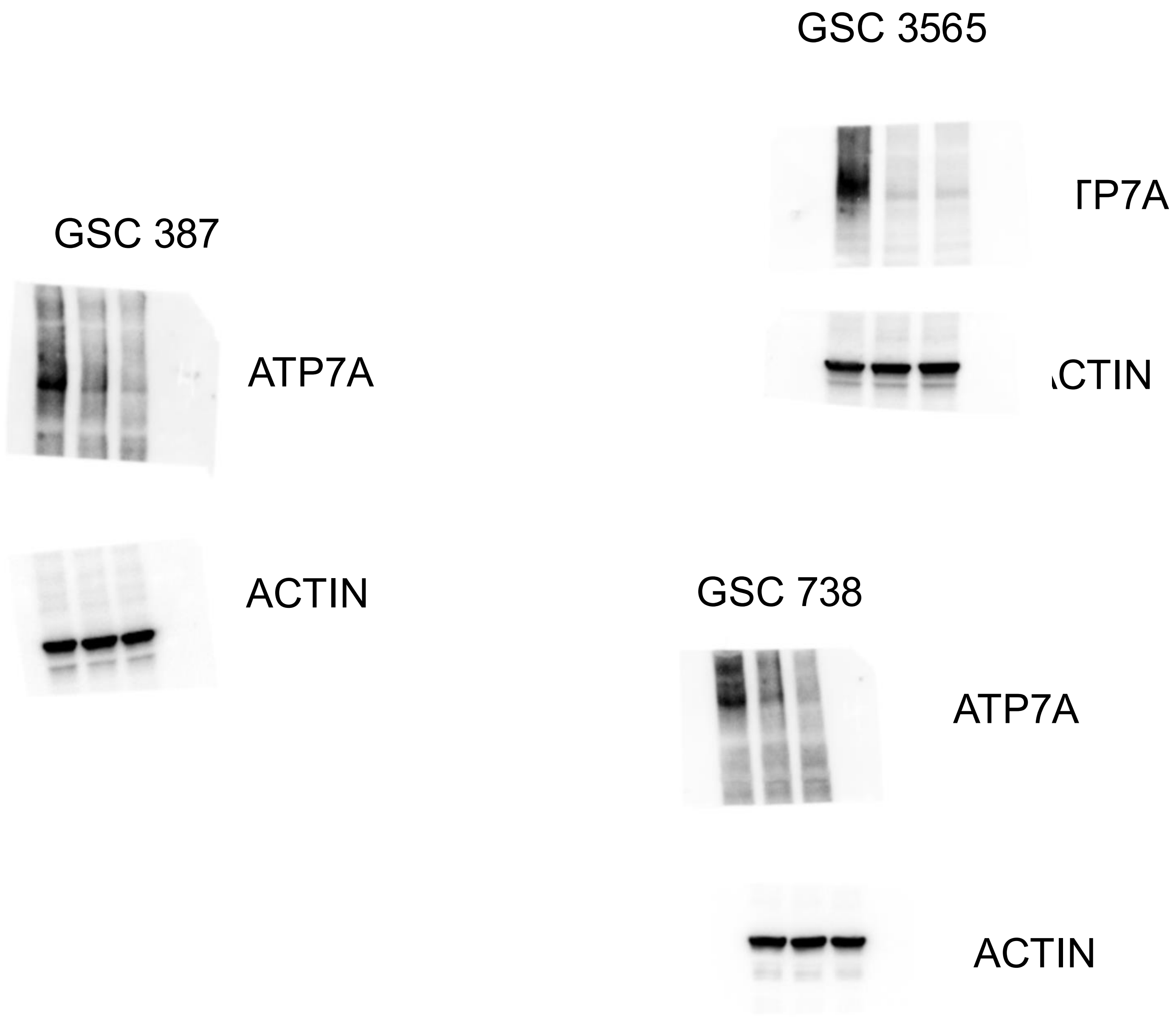

Figure S6C

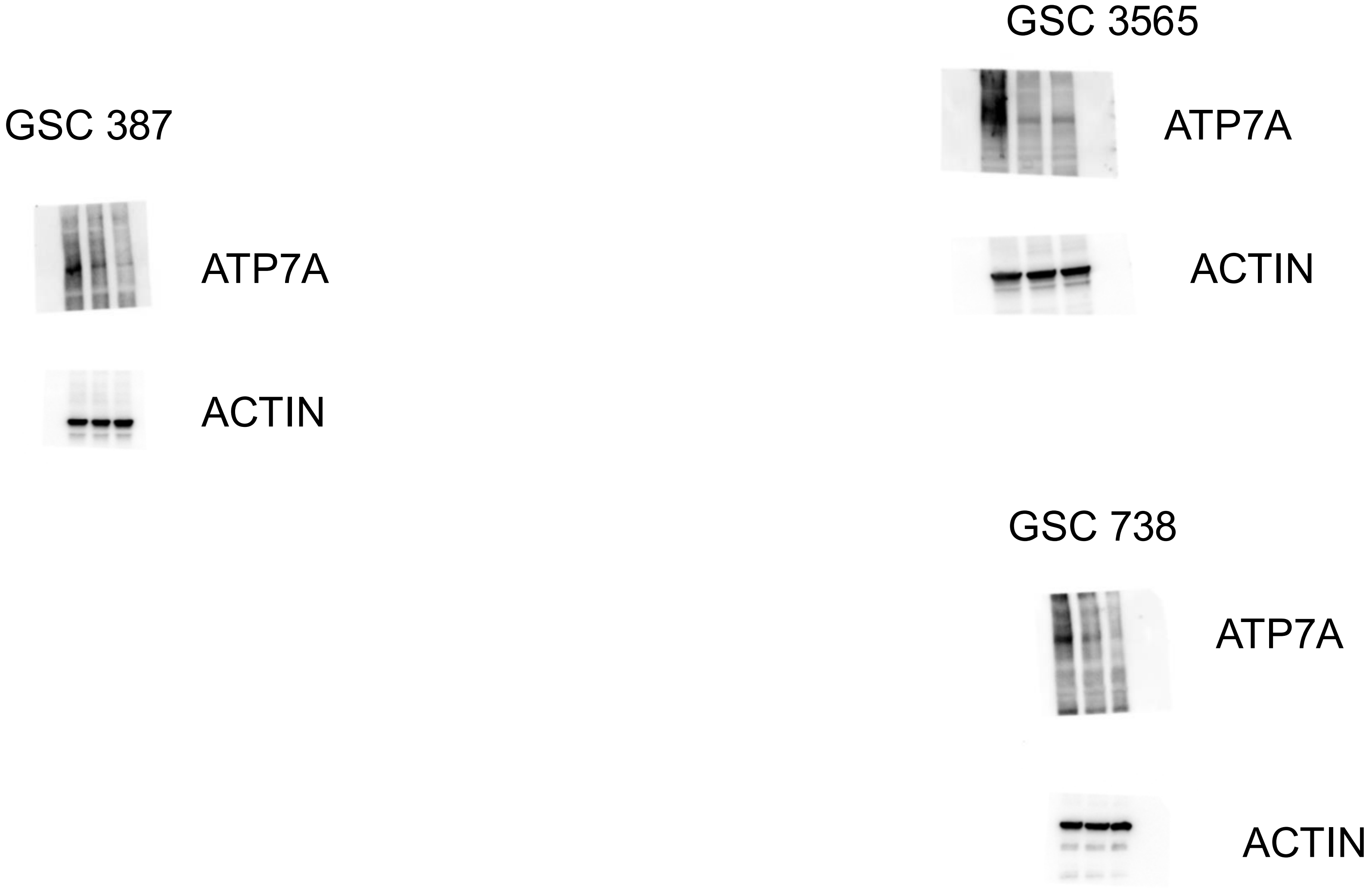

Figure S7E

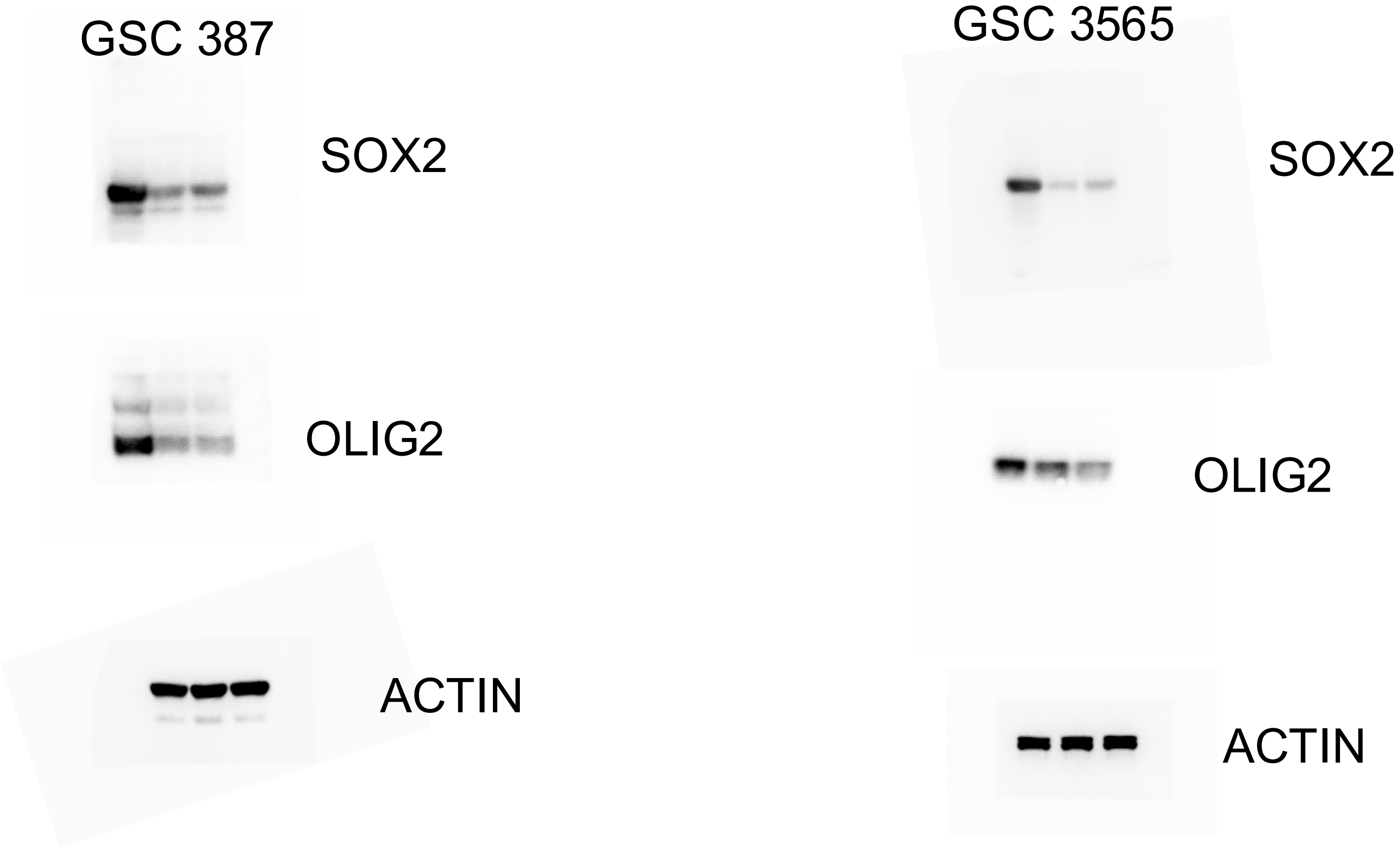

Figure S8A

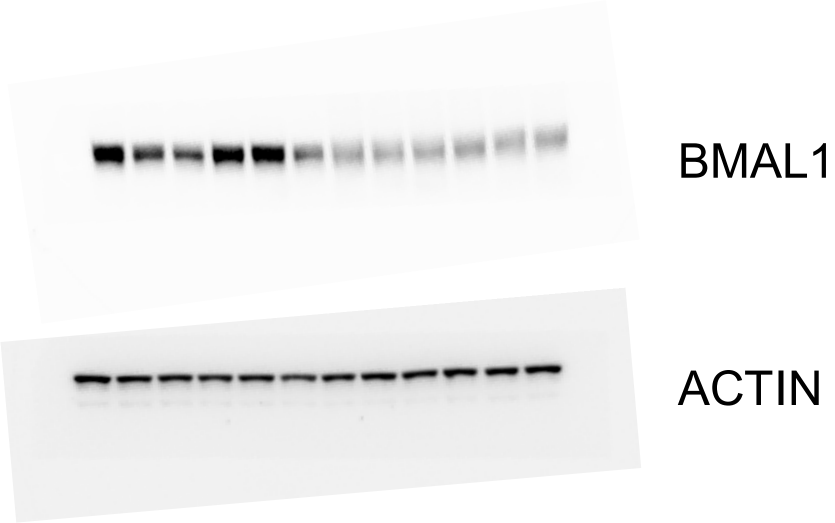

Figure S8C

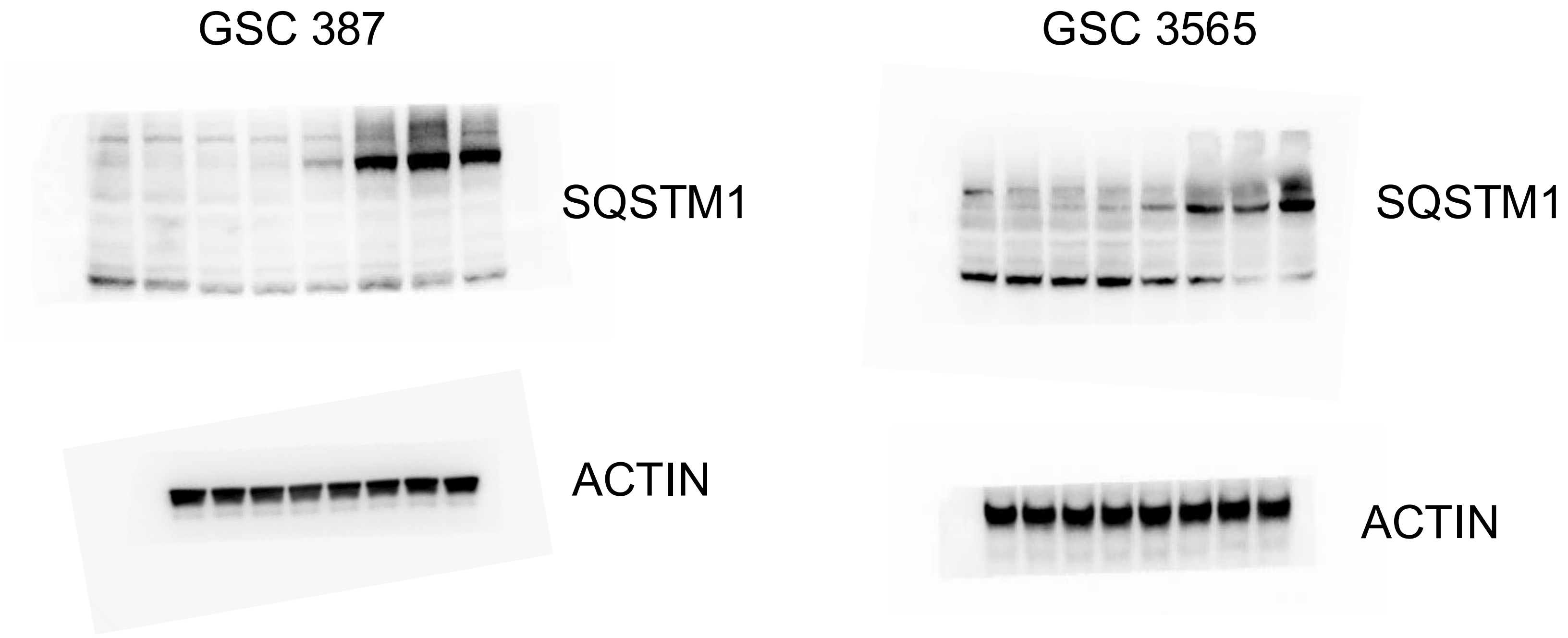

Figure S8D

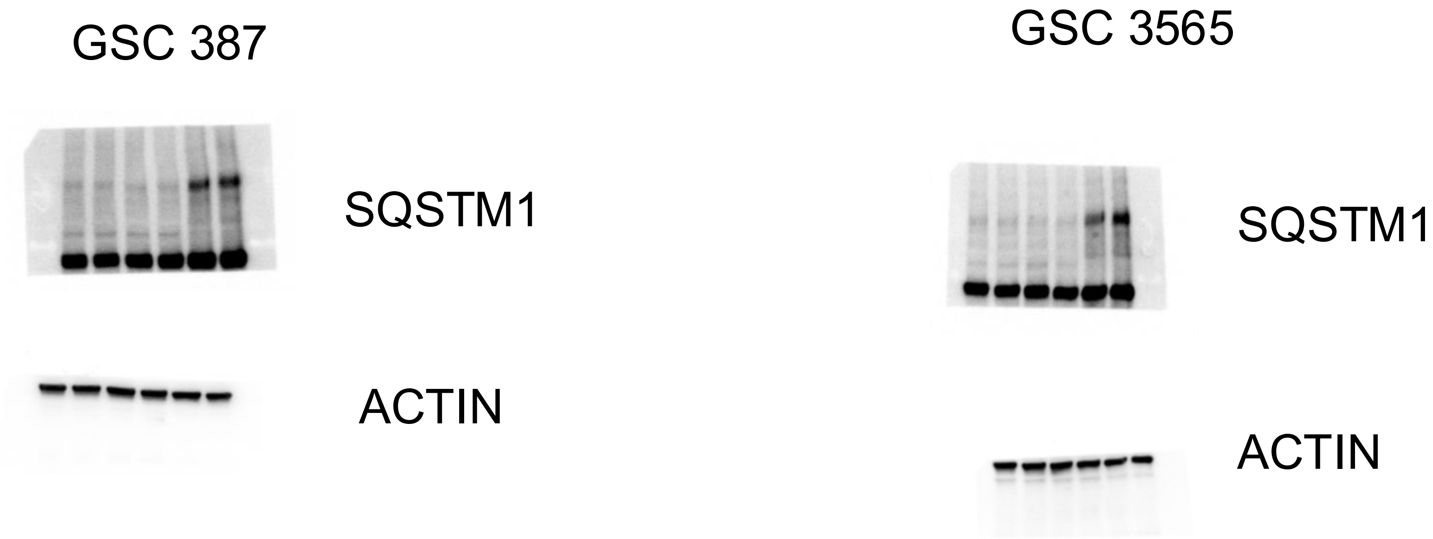

Figure S8E

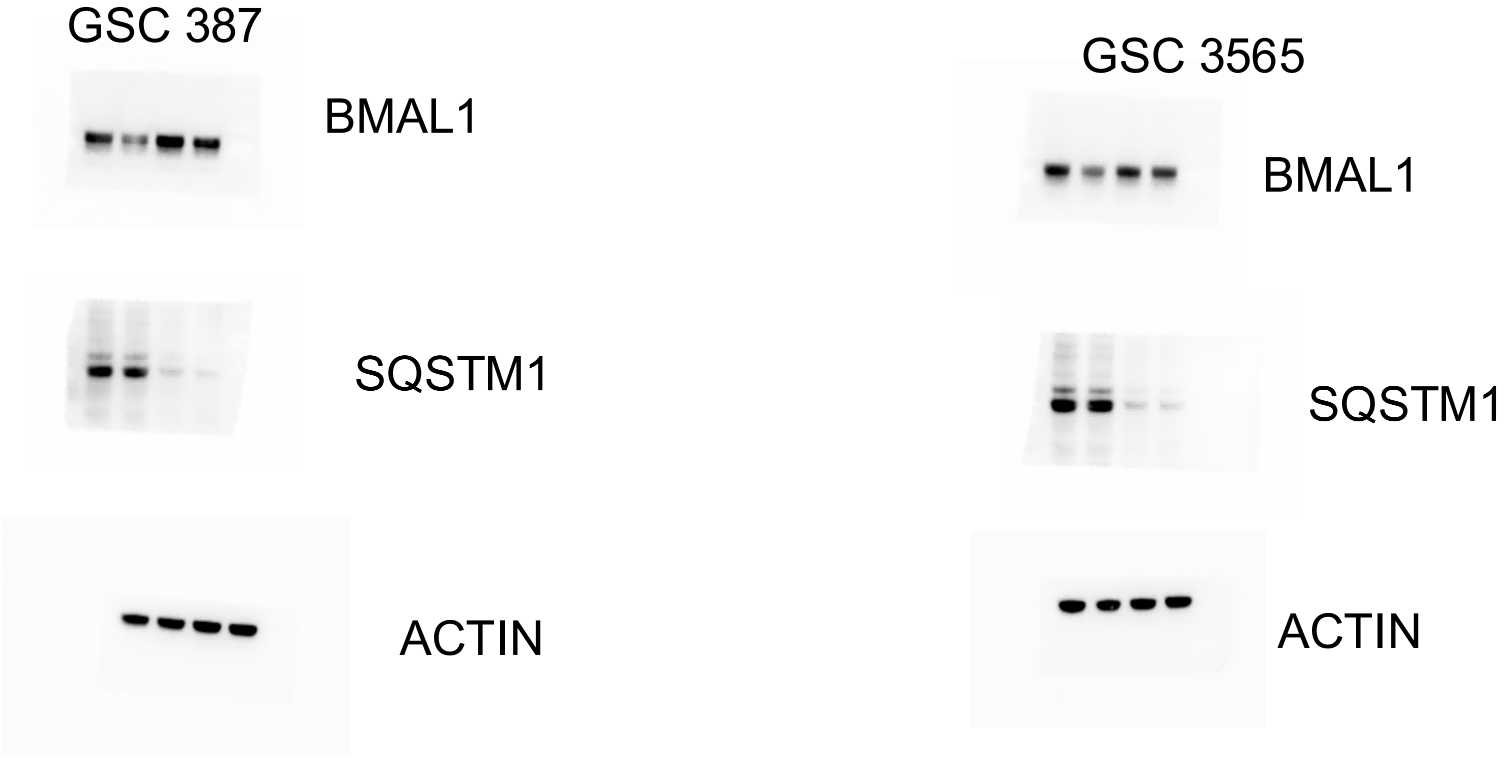

Figure S8F

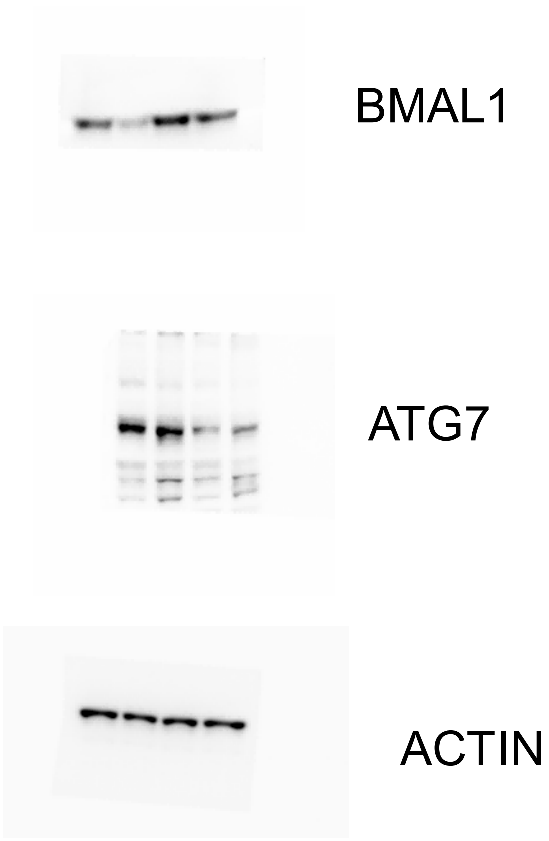

Figure S8G

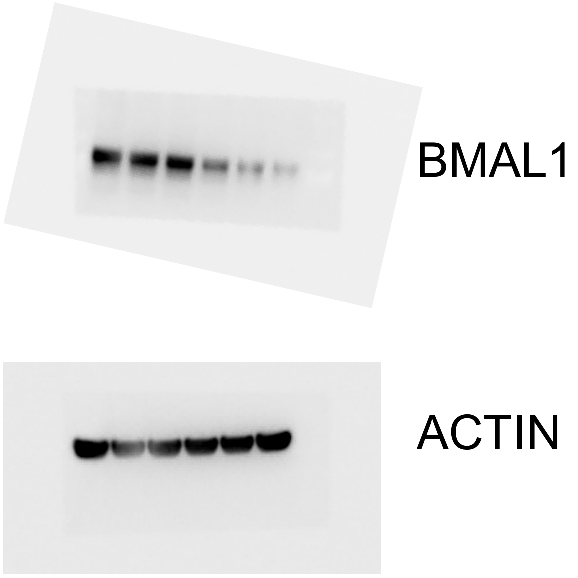

Figure S8H

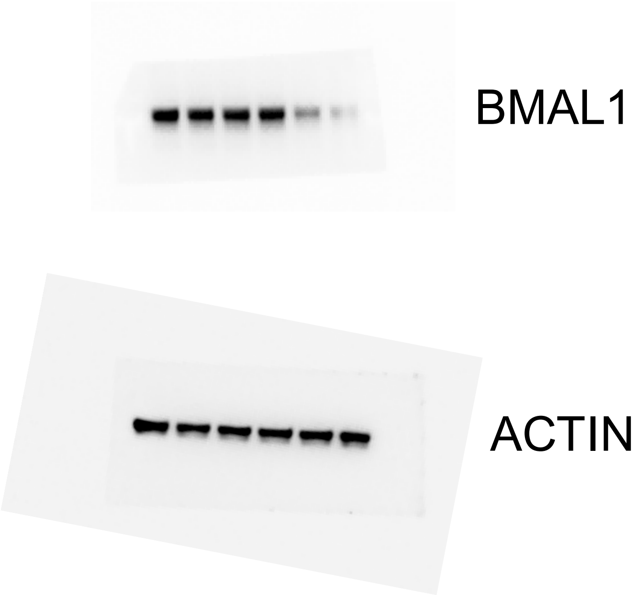

Figure S9A

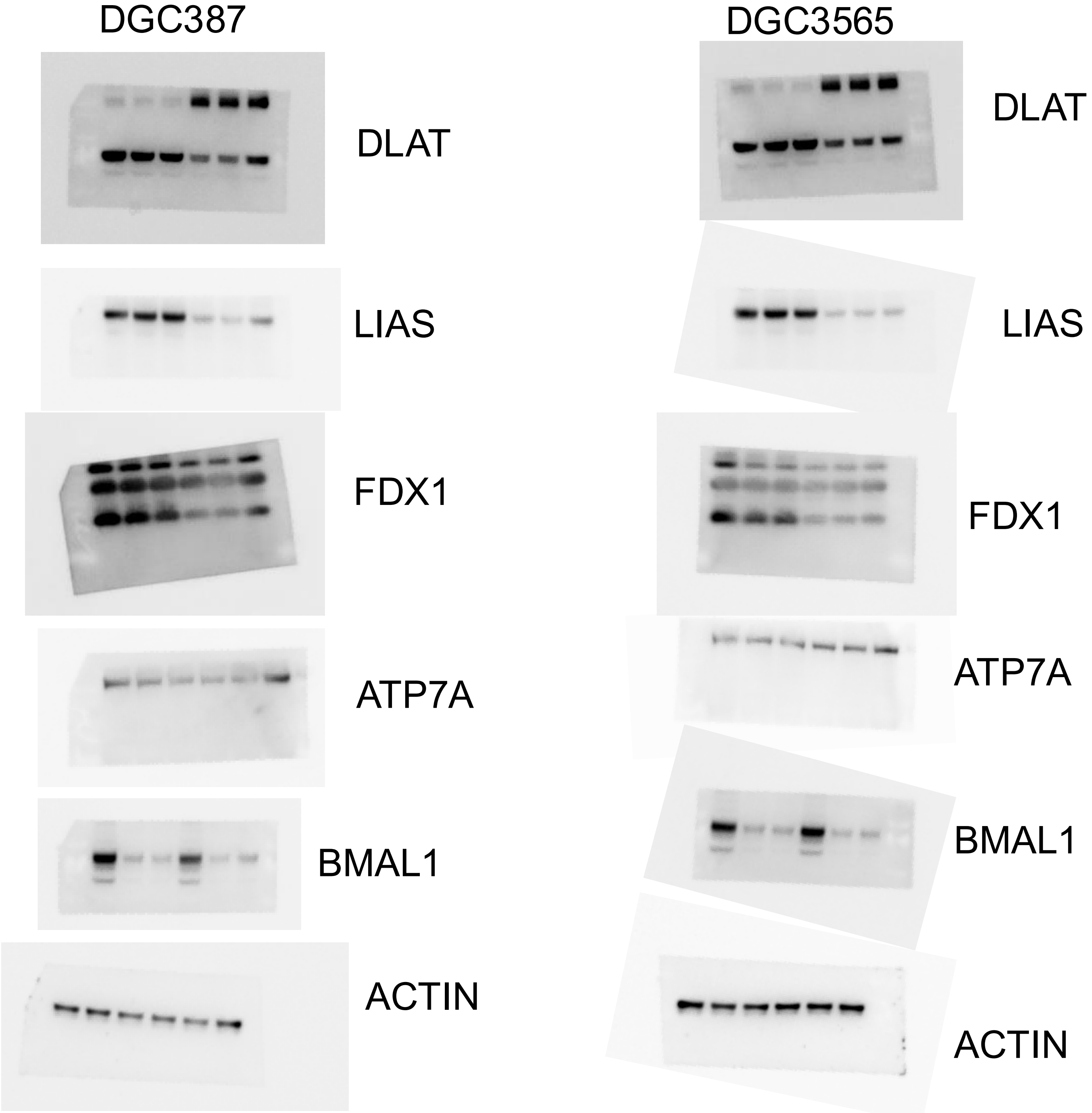
DGC387
DGC3565
DLAT
LIAS
FDX1
ATP7A
BMAL1
ACTIN
DLAT
LIAS
FDX1
ATP7A
BMAL1
ACTIN
